# Self-supervised learning for characterising histomorphological diversity and spatial RNA expression prediction across 23 human tissue types

**DOI:** 10.1038/s41467-024-50317-w

**Published:** 2024-07-13

**Authors:** Francesco Cisternino, Sara Ometto, Soumick Chatterjee, Edoardo Giacopuzzi, Adam P. Levine, Craig A. Glastonbury

**Affiliations:** 1 Human Technopole, Viale Rita Levi-Montalcini 1, 20157 Milan, Italy; 2 Research Department of Pathology, University College London, London, UK

**Keywords:** Machine learning, Genetic variation, Genetic association study

## Abstract

As vast histological archives are digitised, there is a pressing need to be able to associate specific tissue substructures and incident pathology to disease outcomes without arduous annotation. Here, we learn self-supervised representations using a Vision Transformer, trained on 1.7 M histology images across 23 healthy tissues in 838 donors from the Genotype Tissue Expression consortium (GTEx). Using these representations, we can automatically segment tissues into their constituent tissue substructures and pathology proportions across thousands of whole slide images, outperforming other self-supervised methods (43% increase in silhouette score). Additionally, we can detect and quantify histological pathologies present, such as arterial calcification (AUROC = 0.93) and identify missing calcification diagnoses. Finally, to link gene expression to tissue morphology, we introduce RNAPath, a set of models trained on 23 tissue types that can predict and spatially localise individual RNA expression levels directly from H&E histology (mean genes significantly regressed = 5156, FDR 1%). We validate RNAPath spatial predictions with matched ground truth immunohistochemistry for several well characterised control genes, recapitulating their known spatial specificity. Together, these results demonstrate how self-supervised machine learning when applied to vast histological archives allows researchers to answer questions about tissue pathology, its spatial organisation and the interplay between morphological tissue variability and gene expression.

## Abstract (web summary)

Digitisation of vast archives of histology slides provides opportunities to use machine learning to identify specific tissue substructures associated with disease. Here the authors use self-supervised learning on 1.7 million histology images from 23 human tissues. The model segments tissues, detects pathologies and predicts RNA expression, linking morphology and gene expression.

## Introduction

Histology is a relatively inexpensive and effective technique that is commonly used to diagnose and characterise a multitude of diseases, most notably, cancer. Classically, glass histology slides are examined by a pathologist under a microscope; however, recently, there has been considerable momentum in digitising pathology workflows, as histology slides can be quickly scanned at high resolution (40×) to generate Whole Slide Images (WSI). This digitisation provides an opportunity to leverage several advances in computer vision and machine learning (ML). Indeed, multiple ML methods have been developed, largely for malignant pathological entities, to segment specific cell types, tissue structures, diagnostic features of interest^1–4^, predict the mutation status of tumours^5^ and diagnostically classify histology tissue sections^3^. Whilst such supervised learning algorithms have proved successful, they rely on expert crafted labels. Recently, self-supervision has proven to be a useful methodology to learn rich, low-dimensional representations of imaging data that have shown competitive performance to supervised methods, but can be used for a wide variety of downstream tasks.

In parallel, there are ongoing large-scale research efforts to collect both histology and paired molecular data from thousands of samples, including RNA sequencing (RNA-seq) and Whole Genome Sequencing (WGS)^6,7^. Such datasets provide an opportunity to learn how tissue structure and function vary in a population, and how constituent elements of tissue, in both health and disease, are impacted by both common genetic variation and gene expression. Previous efforts have focused on supervised approaches, by extracting and quantifying the size and distribution of specific cell-types of interest and characterising them epidemiologically and genetically. However, this requires the manual collection of binary segmentation labels of cells which is time consuming and therefore does not scale to multiple tissue types. Supervised methods utilising ImageNet pre-trained models have also been developed that aim to predict tissue of origin^8^, or gene expression directly from histology image tiles, but have not sought to decompose gene expression contributions from underlying tissue substructures and pathological features present in the tissue^9–11^. Seminal work on unsupervised approaches that have aimed to couple histology, gene expression and genetic variation have focused on the use of latent factor models^12–14^. These approaches have been able to characterise both shared and specific sources of gene expression and histological variation and have described “image QTLs”, in which genetic variants drive changes in tissue morphology. Finally, the extent to which specific histological tissue substructures and pathological features vary naturally in a population, as quantified computationally, and hence objectively, from large numbers of WSI, has not been widely addressed, nor how such variation can be associated to common genetic variants or changes in gene expression related to tissue morphology.

**Fig. 1:**
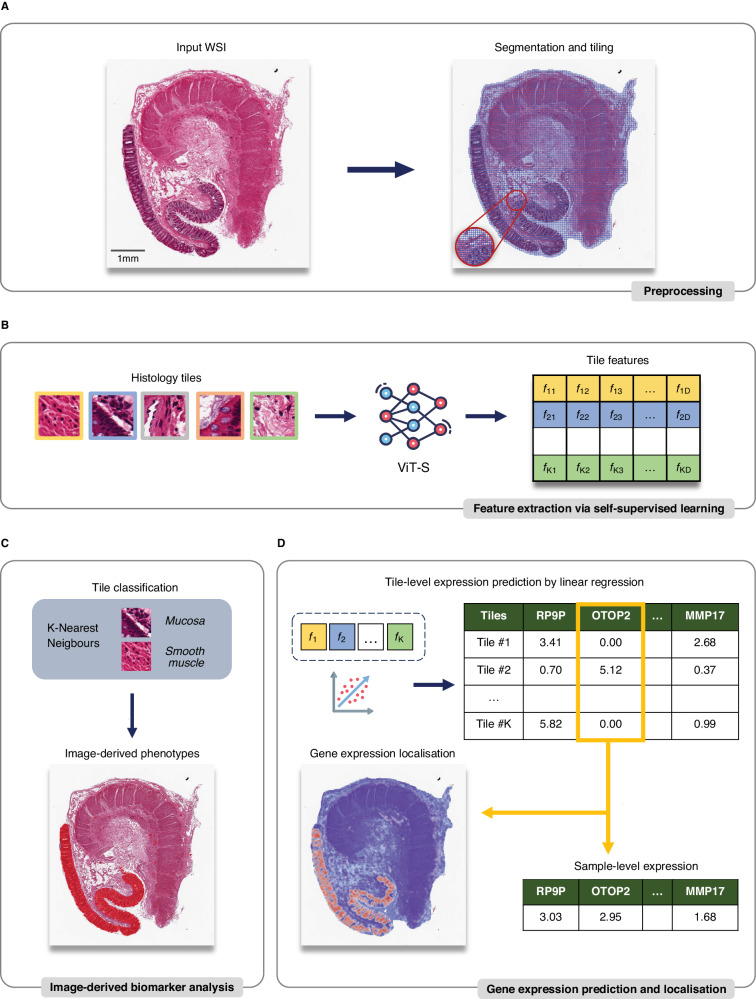
Schematic representation of using self-supervised representations learnt from whole slide image histology for segmentation of tissue substructures, pathological features and understanding morphology-expression-genetic associations using RNAPath. **A** Histology whole slide images (WSI) are preprocessed by segmentation and tiling into 63 × 63 μm^2^ squared regions. **B** Self-supervised learning is used to extract morphological features from tiles. **C** By using learned features, tiles are classified through a K-Nearest Neighbours model and phenotypes—in terms of extent of detected regions in the sample—are derived. **D** RNAPath model takes as input tile embeddings and predicts both local (tile-level) and global (sample-level) gene expression as output, together with the heatmap to visualise predicted spatial gene activity.

We advance previous work by exploiting several recent ML innovations, namely Vision Transformers (ViT)^15^ coupled with self-supervised learning^16^ to combine histology, gene expression and common germline genetic variation in 13,898 samples, representing 23 distinct tissues, from 838 donors and a total of 1.7 M histology tiles (Fig. 1). We start by learning low-dimensional representations of histology tissue tiles using DINO, demonstrating that our representations are able to identify and cluster specific tissue substructures, cells and pathological features without labels (Fig. 1A, B).

We utilise the ability of these representations to identify substructures and pathological features to automatically segment all GTEx` WSI, with limited manual labelling of specific features (< 0.5% of the data used) obtaining substructure and pathology proportions per donor sample (Fig. 1C). Next, using these derived substructures and pathological features, we characterise profound tissue variability across donors that drives substantial differential gene expression, as well as characterise common germline genetic variants associated with specific histopathological features through both genome-wide association analysis (GWAS) and interaction eQTL analyses. Despite not being trained specifically for this task, as our representations are rich morphological descriptors of tissue histology both within and across donors, we are able to predict and spatially localise individual RNA expression levels with superior performance to competing methods (Fig. 1D). We validate our spatial RNA expression predictions using positive control immunohistochemistry (IHC) for several canonical marker genes and subsequently characterise the specific localised expression signatures of 29 individual substructures and pathological features (accuracy > 80%). Finally, we evaluate both our histology tile representations and RNAPath on an held-out external validation cohort, TCGA-BRCA and demonstrate we can segment carcinoma from benign tissue. Here, we have shown that learning self-supervised representations of histology is a powerful approach for understanding the molecular characteristics of tissue and its collective organisation.

## Results

### Histology tile representations learnt via self-supervision distinguish tissue substructures and pathological features without labels

**Fig. 2:**
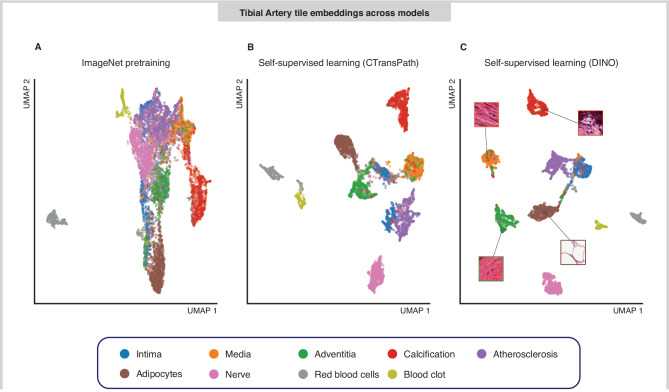
UMAP embeddings of tibial artery tile features from three representation learning methods. **A** ResNet50 with pretrained weights from ImageNet. **B** CTransPath (pretrained on 15 M histology images). **C** Our self-supervised ViT-S model trained using self-distillation with no labels (DINO). Tiles have been manually labelled with tissue substructures/pathologies to interpret clusters. DINO embeddings show both better qualitative clustering and quantitative silhouette scores, a 43% improvement over CTransPath.

We utilised 13,898 Whole Slide Images (WSI) across 23 tissues collected from 838 donor individuals as part of GTEx^7^. WSI are gigapixel images (e.g. 50,000 × 150,000 pixels), in which much of the image does not contain tissue. To obtain only tissue containing sections of the WSIs, we segmented the tissue from background using a previously published U-net (Supplementary Fig. 1). Self-supervised models have been recently shown to be effective in learning compact, rich image representations^17–22^. Therefore, we sought to learn relevant features present in histology images by training a Vision Transformer (ViT-S) on 1.7 M GTEx histology tiles using the self-supervised DINO framework^16,23^ (see Methods). Despite being trained with no labels, we see that learnt representations clearly capture and separate cell types (e.g. adipocytes), pathological features (e.g. arterial calcification) and tissue substructures (e.g. tibial vessel layers: intima, media, adventitia) (Fig. 2, Supplementary Fig. 2 for additional tissues) whilst not being affected by confounding related to where samples was processed or the hospital the donor enrolled at (Supplementary Fig. 3) (see methods). We compared our self-supervised representations learnt from GTEx, to both representations obtained from a ResNet50 model pre-trained on ImageNet and a self-supervised Swin transformer trained on 15 million histology images, CTransPath^24^. We see that our representations better capture intrinsic tissue substructures with a consistent improvement in median silhouette score across a range of k-mean clusters [3,20]: ImageNet embeddings = 0.092, CTransPath embeddings = 0.137, DINO embeddings = 0.196 (Tibial Artery); ImageNet embeddings = 0.103, CTransPath embeddings = 0.138, DINO embeddings = 0.225 (Esophagus Mucosa). (Supplementary Fig. 4). With the aim of assessing the impact training has on DINO embeddings, we performed a 3-fold cross validation on a subset of the data (500k tiles from 7 tissues each), excluding any sample that contained annotated patches. Tile clustering was consistent across folds, as confirmed by the silhouette scores: 0.190, 0.195 and 0.193 for fold 1, 2, 3 respectively (std = 0.003) for tibial artery and 0.219, 0.228 and 0.226 for fold 1, 2, 3 respectively (std = 0.005) for esophagus mucosa (Supplementary Figs. 5, 6).

### Self-supervised WSI tissue substructure and pathology segmentation

**Fig. 3:**
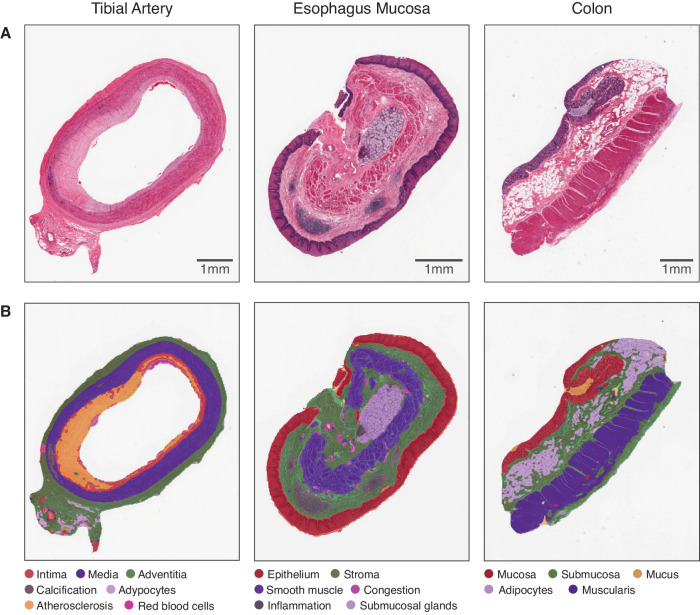
Pathology and tissue substructure segmentation of GTEx histology samples. **A** H&E WSI of tibial artery, esophagus mucosa and colonic tissue. **B** Segmentation of substructures and localised pathological features via k-Nearest neighbours on tile features. WSI tissue types have been labelled according to their GTEx descriptor which may not perfectly represent the specimen (e.g. the esophagus mucosa example includes submucosa).

Segmenting various tissues into constituent tissue substructures is a time consuming task that does not scale easily to thousands of WSI. Additionally, it has not been widely documented how normal tissue substructures vary in a population or which genes are specific to each substructure or pathological feature. To automate the dissection of tissue into constituent substructures and pathological features, we manually labelled a small subset of image tiles from a range of tissue types, with annotations that were validated by a clinical histopathologist (see Methods). Fixing the ViT-S encoder, we performed inference on the WSI tiles as well as the subset of labelled tiles to obtain their corresponding representations. We trained a k-Nearest Neighbours (kNN) model using the labelled tile representations and inferred the class of each unlabelled tile originating from a given WSI (see Methods). First, we benchmarked our approach against Resnet and CTransPath for classifying labelled tiles using a kNN. We see that the average accuracy achieved using our DINO model embeddings outperforms other approaches in all tissues tested: tibial artery (DINO = 0.937, CTransPath = 0.897, ImageNet = 0.757), esophagus mucosa (DINO = 0.909, CTransPath = 0.888, ImageNet = 0.786) and colon (DINO = 0.956, CTransPath = 0.949, ImageNet = 0.907). Additionally, by mapping annotated tiles back to the original histology, we see that our tile-classification-based approach for tissue segmentation clearly detects known tissue substructures and pathological features (Fig. 3). We quantitatively evaluated the accuracy of the kNN in the tile-level classification: for each tissue, we held-out 10% of the annotated tiles of each class from the kNN model fitting and measured its accuracy across 10 folds. Median accuracy across all derived tissue substructure and pathological features was 92% ± 3.5%.

To further assess our automated segmentation quantitatively, we compared calcified cases as described in the GTEx pathology notes versus our inferred calcification labelled tiles per WSI. Tiles inferred as belonging to the calcification class had high sensitivity in recovering ground-truth pathologist labels, with AUROC = 0.93, sensitivity = 89.7%, specificity = 79.0% (Supplementary Fig. 7), indicating that the vast majority of true positive cases were correctly identified by the kNN segmentation model. Additionally, when considering calcification occupying > 5% of the tissue present in a WSI, our model identifies a further eight cases. Upon manual inspection, five of these eight cases were false positives containing debris that resembled calcification; however, three WSI clearly contained calcification (false negatives) that were not labelled as such in the GTEx pathology notes (Supplementary Fig. 8). This highlights the utility of our approach to discover unannotated pathological features, with the potential to aid pathological reporting. Finally, we evaluated the kNN robustness across the three DINO training folds: the median accuracy was highly comparable: 0.930 ± 0.005 for tibial artery and 0.904 ± 0.007 for esophagus mucosa.

The full list of tissue substructures and pathological features with corresponding accuracy and variability across 10-folds are presented in Supplementary Tables 1, 2.

### Substantial variability in tissue substructure proportions across donors

**Fig. 4:**
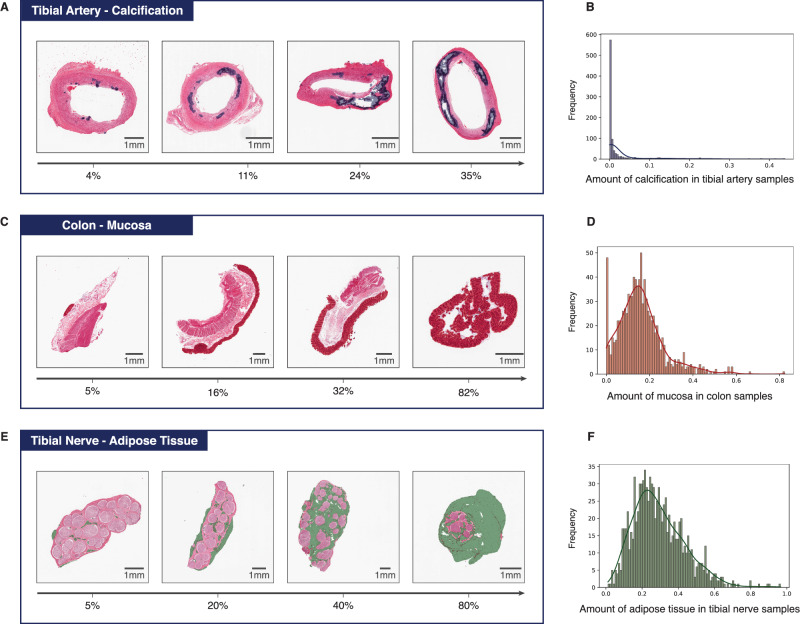
Pathology and tissue substructure segmentation and its inter-donor variability. Examples of differential tibial artery calcification (**A**) and its cohort variability (**B**). Colonic mucosa variability (**C**, **D**) and tibial nerve adipose tissue (**E**, **F**).

Whilst GTEx pathology notes contain labels of whether subjects have a given pathological feature or not, there is no information on its extent, i.e. the proportion of affected tissue, nor its spatial location within the tissue. Using our kNN segmentation model, we inferred labels for all tissue tiles across all WSI considered. By doing so, we can represent any given sample as the proportion of its inferred tissue substructures and pathological features, allowing us to quantify inter-subject variability. We see that the proportion of different tissue substructures and pathological features vary dramatically across donors within the same tissue type (Fig. 4, Supplementary Fig. 9). For example, the proportion of calcified tissue in tibial artery samples varies from 0 to 44% with a mean of 3.3%. This pattern is true across all tissues and substructures quantified (Supplementary Table 3). In some but not all cases, this likely represents tissue sampling variation as opposed to true biological or pathological variation.

An acute difficulty when dissecting tissue without laser capture microdissection (LCM) is obtaining the correct target tissue of interest with little to no contamination of other tissue components. This is critical for enabling the precise characterisation of gene expression tissue specificity or quantifying the degree of tissue sharing across eQTLs^25–27^. For example in GTEx, ‘esophagus mucosa’ tissue is defined as having mucosal epithelium present, whilst ‘esophagus muscularis’ tissue should not. To determine the presence of a contaminant or incorrect target tissue, we assessed the degree to which squamous epithelium was present in muscularis samples. Surprisingly, 6% of muscularis samples (total *n* = 950) contain mucosal tissue (>1%) (Supplementary Fig. 10). To determine whether this is recapitulated at the level of gene expression, we checked the expression level of a gene specific to mucosal epithelium, *KRT6A*. We see that there is substantial expression of *KRT6A* in 8.8% of GTEx esophagus muscularis samples (> 20TPM), confirming both the histology and RNA-seq contain non-target tissue.

Similarly, varying amounts of adherent adipose tissue is commonly present in a variety of GTEx samples due to imperfect histological dissection. For example, 84% of coronary arteries and 95% of tibial nerve samples have > 10% of the specimen composed of adipose tissue. Indeed, using a highly specific gene expression marker of adipocytes, *PLIN1*, we see that after subcutaneous adipose tissue (median TPM = 970), visceral adipose tissue (median TPM = 542) and breast mammary tissue (median TPM = 310), tibial nerve (median TPM = 40) and coronary artery (median TPM = 30) have the highest *PLIN1* expression across all 54 tissues. These findings suggest there is significant inter-tissue donor variability in GTEx histology and hence derived RNA-seq, but also substantial “contamination” of tissue substructure types across tissue classes in GTEx. Importantly, this affects estimates of eQTL tissue-sharing, with tibial nerve, mammary, subcutaneous and visceral adipose tissue having the largest degree of tissue-shared eQTL effects across tissues^25^. Whilst tissue sharing eQTLs between different fat compartments (i.e. subcutaneous and visceral) and breast tissue would be expected, sharing of adipose-nerve eQTL effects is most likely due to the adipocyte fraction present in GTEx nerve samples rather than any underlying biological sharing of nerve-tissue specific eQTLs with adipose tissue. Our findings suggest that estimates of tissue sharing eQTLs are likely inflated and that LCM, single-cell RNA-seq and spatial technologies at scale will likely revise these estimates downwards.

### Sex and age-specific variability in tissue substructure and pathological features

Having quantitative measures of tissue substructures allows us to assess the histological impact of age and other epidemiological variables on tissue structure and its variability in a population. To address this systematically, we fit linear models to investigate whether variability in any of the 29 tissue substructure or pathological features quantified with accuracy > 80% (see Methods) across donors had sex, age, BMI or ischemic time specific effects. We find 18 sex, 19 age, 4 BMI and 19 ischemic time significant associations (see Data and Code Availability to download full summary statistics). For example, we see that the amount of arterial calcification (*P*-value = 7.2 × 10^−15^, β = 0.033) and atherosclerosis (*P*-value = 1.52 × 10^−13^, β = 0.023) increase with age, and atherosclerosis is more common in males (*P*-value = 1.4 × 10^−4^, β = −0.30). Many of the significant associations were confirmatory and expected, for example breast lobules being almost exclusive to female breast tissue (*P*-value = 2.5 × 10^−36^, β = 0.99), the amount of solar elastosis in sun exposed skin increases with age (*P*-value = 1.57 × 10^−32^, β = 0.04), and autolysed mucosa capturing ischemic time effects (*P*-value = 2.04 × 10^−34^, β = 0.001). Interestingly, we find a link between gynecomastoid hyperplasia and age (*P*-value = 2.81 × 10^−8^, β = 0.01). This is likely due to increased adiposity in older age (both sexes) and decreased testosterone production in older men^28^.

Finally, adipose tissue abundance in breast tissue is known to increase with age, and this increased adiposity is associated with risk of breast cancer^29^. We demonstrate our derived adipose proportions are associated with age in female breast mammary tissue samples (*P*-value = 8.5 × 10^−4^, β = 1.9 × 10^−2^). This effect was robust to BMI adjustment (*P*-value = 3.5 × 10^−4^, β = 5.9 × 10^−2^) whilst the same effect was not observed in male donors, despite being better powered (*P*-value = 0.75, β = −1.68 × 10^−3^). This demonstrates the ability of our approach to find epidemiological links between tissue substructures and pathological features in WSI. The integration of WSI (generated from either archival material or through routine digital pathology workflows) with detailed electronic healthcare records could prove useful to discover additional novel, prognostic and epidemiologic associations.

### Pervasive differential expression driven by substructure and pathology variation across tissues

We sought to assess the extent to which gene expression is impacted by tissue substructure variation between donors across a given tissue by differential expression analysis (see Methods). We observe pervasive differential expression within tissues and their constituent tissue substructures, with median = 1753 number of genes (FDR1%) being differentially expressed (see Data and Code Availability to download full summary statistics). Interestingly, even for individual tissue substructures that make up the majority of a particular tissue, such as dermis in skin (1955 FDR1%), tunica media in tibial artery (12,810 FDR1%) and nerve bundles in tibial nerve (751 FDR1%), there is significant differential expression between samples. These findings highlight the tissue sampling variability present in GTEx, in which the underlying proportions of each tissue has substantial inter-donor variability. In the extremes, this can represent tissue samples within a tissue class that do not resemble the same underlying target tissue that was supposed to be acquired (Supplementary Fig. 11).

We first investigated differential gene expression (DE) enrichment in substructures with known positive controls. For example, 1484 differentially expressed genes (FDR1%) were detected for adipocyte proportion across coronary artery samples. Reassuringly, the top differentially expressed gene was *LIPE* (β = 0.46, *P*-value = 4.43 × 10^−9^), a selective marker for adipocytes, as well as *ADIPOQ*, *PLIN1*, *PLIN5* and *CIDEC* (*P*-value < 1 × 10^−7^), all genes known to be specifically expressed in adipocytes. As expected, Gene Set Enrichment Analysis (GSEA) confirmed adipose tissue to be the most likely tissue type (*P*-value = 2.33 × 10^−71^). Similar results were obtained for other well-described tissue substructures, such as submucosal glands in esophagus mucosa being enriched for genes associated with gastric epithelial cells (*P*-value = 1.39 × 10^−33^) with top DE genes including *SPDEF* (*P*-value = 9.87 × 10^−23^), a gene required for mucous cell differentiation^30^, as well as *MUC5B* (*P*-value = 6.07 × 10^−16^), a specific marker of mucin secreting epithelial cells^31^. Similarly, levels of inflammation in esophagus mucosa were enriched for peripheral blood cells (*P*-value = 8.68 × 10^−30^) with top DE genes representing broad lymphocyte markers (e.g. *LTB*, *CD5*, *CD6*, *CD48;* *P*-values all < 1 × 10^−18^). All these confirmatory results provide reassurance that our derived proportions capture specific tissue substructures and that we are able to relate inter-donor variation in such substructures to changes in RNA levels.

Similar to quantified tissue substructures, we investigated genes differentially expressed due to differential amounts of pathological features across donors. For atherosclerosis proportion in tibial artery, 6121 DE genes were detected at FDR 1%. Top cell-type enrichments were T-memory cells, NK-cells and endothelial cells (*P*-value < 1 × 10^−16^). Macrophages, known as foam cells in atherosclerotic plaques, were also enriched but to a lesser extent (*P*-value = 1.36 × 10^−3^).

For tibial artery calcification, a co-morbid pathology of atherosclerosis, we identified 1794 differentially expressed genes (FDR1%). Two of the most significant genes were *DUSP4* (β = 0.25, *P*-value = 2.19 × 10^−16^), known to play a role in calcium homeostasis and *KCNN4* (β = 0.21, *P*-value = 2.09 × 10^−11^), a calcium activated potassium channel shown to induce vascular calcification^32^. Enrichment analysis demonstrated macrophages (*P*-value = 8.47 × 10^−40^) to be the most enriched cell-type. Macrophages are known to play an important role both in atherosclerosis and concurrent arterial calcification, with recruitment of macrophages shown to drive increased osteogenic calcification whilst displaying a pro-inflammatory phenotype. Collectively, these enrichments represent the known interplay in atherosclerosis between intima endothelial cells and the chronic inflammation and fat deposition taking place in atherosclerotic arteries.

Finally, as calcification is reported in the GTEx pathology notes, we sought to compare our continuous measure of calcification derived from the WSI with the reported presence or absence of calcification in the GTEx pathology notes. To do so, we divided samples (*n* = 579) between healthy (*n* = 442) and calcified (*n* = 137) according to the pathology notes and tested for differential expression in a linear model, whilst correcting for confounders (see Methods). We identified 1025 differentially expressed genes after FDR1% correction versus 1794 when using our WSI-derived continuous measure of calcification. Whilst 78% of the differentially expressed genes found using the GTEx reports are shared between both analyses, our results suggest we benefit from increased power when assessing continuous measures of calcification rather than just its presence or absence, as well as the identification of genes associated with amount of calcification rather than merely its presence (Supplementary Fig. 12).

### Genetic association and detection of interaction eQTLs driven by tissue substructure and pathological variation

As pathologies such as calcification are complex traits, we assessed whether derived pathological feature proportions are associated with common genetic variation (see methods). To do this, we performed GWAS on four derived pathologies: coronary and tibial artery calcification as well as inflammation and vascular congestion in esophagus mucosa. Whilst no variants were genome-wide significant, considering suggestive hits (*P*-value < 1.0 × 10^−6^), we find four variants associated with pathological features. All variants have either been previously described in relevant complex disease GWAS or are associated with relevant traits through Phenome-Wide Association Studies (PheWAS). rs971292786-C (β = 0.51, *P*-value = 1.9 × 10^−7^) is associated with levels of calcification in coronary arteries and in a FinnGen PheWAS (Freeze version 8), rs971292786-C is associated with coronary angioplasty (β = 0.055, *P*-value = 4.10 × 10^−5^), with a consistent direction of effect. Coronary angioplasty is the primary surgical procedure used to treat atherosclerotic arteries. For inflammation in esophagus mucosa, we find two variants rs111402007-A (β = 0.53, *P*-value = 7.64 × 10^−7^) and rs35779991-C (β = 0.25, *P*-value = 8.87 × 10^−7^). rs35779991-C is genome-wide significant in a GWAS for Body Mass Index (BMI) (β = 0.018, *P*-value = 9.97 × 10^−12^) whilst rs111402007-A has been previously associated with increased White Blood Cell Count (β = 0.023, *P*-value = 5.8 × 10^−7^). Effect directions are consistent with the known relationship between low-grade systemic inflammation in obesity, and WBC count^33^. Finally, we find a single locus rs4364259-A (β = −0.19, *P*-value = 3.47 × 10^−7^) associated with vascular congestion in esophagus mucosa which has been previously associated with hydroxyvitamin-D levels (β = 0.0158, *P*-value = 2.2 × 10^−308^) (Supplementary Fig. 13).

Similar to previous efforts^12,14,34–36^, we carried out interaction eQTL analyses to identify *cis*-eQTLs whose effect is driven by the amount of tissue substructures and pathological features across donors. By fitting linear models with tissue substructure or a pathological feature as an interaction term, we identified 284 interaction eQTLs (FDR 10%) in 250 unique genes across 31 different phenotypes in eight tissues for which annotations were available. Examples of such interaction eQTLs are visualised in Supplementary Fig. 14. These analyses compare favourably to similar work in which sparse factor models were used to discover 68 abstract image morphology QTLs (imQTLs) across 8 GTEx tissues (FDR 10%)^12^, that could not be linked directly to tissue substructure or function. These results provide further evidence that many bulk-tissue eQTLs could be due to differential amounts of tissue substructure within a tissue type due to experimental sampling variation and/or due to variability and presence of pathological features across donor tissues.

### RNAPath accurately predicts and spatially localises genes in histology WSI

**Fig. 5:**
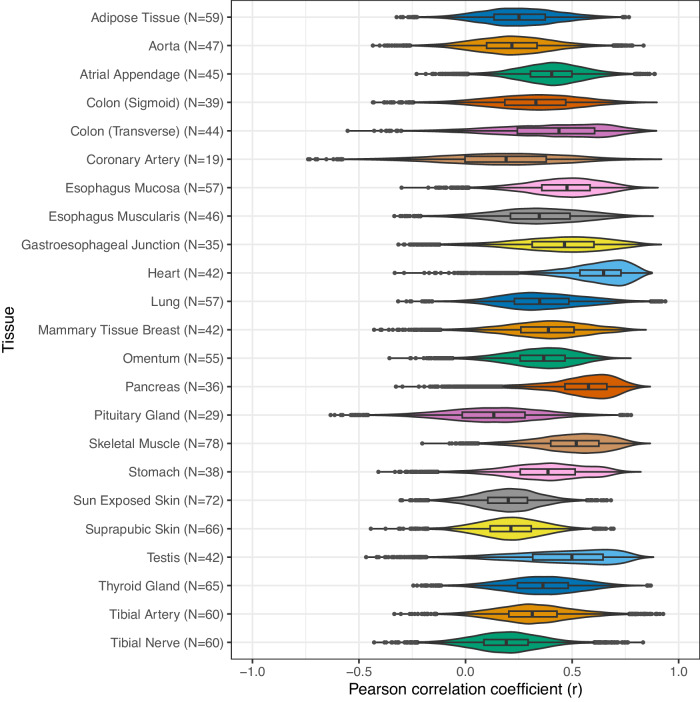
RNAPath accuracy, measured by computing the Pearson correlation coefficient (*r*) between expression prediction and bulk RNA-seq, across 23 GTEx tissues. The number of test samples used to derive statistics is reported next to each model on the *x*-axis. The violin plots depict the distribution of Pearson correlation coefficients, with the inner box showing their median and interquartile range (25th and 75th percentile) and the tails ranging in the interval (Q1 − 1.5 × IQR, Q3 + 1.5 × IQR), across the tested genes (cross-tissue average = 11,327).

As our self-supervised histology tile representations accurately separate known tissue substructures and pathological features, and given paired RNA-seq profiles are available for each donor from the same RNAlater aliquot, we sought to assess whether gene expression influenced by specific histomorphological features could be predicted directly from H&E histology. To do so, we introduce RNAPath, a set of multiple instance learning (MIL) models trained in a tissue specific way (details about training, validation and test set size in Supplementary Table 4), that takes as input histology tile representations and outputs both spatial expression maps for each gene as well as their whole-tissue expression prediction (see Methods). To assess RNAPath’s ability to predict individual RNA abundance at the bulk level, we evaluated the accuracy of bulk RNA-seq prediction by measuring the Pearson correlation coefficient (*r* score) between predicted expression and ground truth (see Methods). Median *r* score across all genes varies substantially across tissues, with the best performance in heart (median *r* score = 0.65) and worst performance in pituitary gland (median *r* score = 0.13) (Fig. 5, Supplementary Table 5). At the individual gene level, we find that 4435 genes can be predicted extremely accurately from histology alone, with an *r* score ≥ 0.75 in at least one tissue. We measured the robustness of the model through a 5-fold cross validation on three tissues, with the smallest (coronary artery, *n* = 239), median (breast, *n* = 456) and largest (skeletal muscle = 797) sample size available. The accuracy of predictions grows linearly with the dimension of the training set (median *r* score = 0.181 ± 0.028 for coronary artery, 0.346 ± 0.038 for breast and 0.484 ± 0.021 for skeletal muscle) as well as the model robustness, with the standard deviation of gene correlation coefficients across folds having a negative correlation with sample size (0.187 ± 0.069 for coronary artery, 0.128 ± 0.051 for breast and 0.082 ± 0.033 for skeletal muscle) both in the validation and test set (Supplementary Fig. 15).

We also tested whether RNAPath results are invariant to DINO encoders trained on different folds (Supplementary Fig. 5). We see consistent results: the mean accuracy of predicted gene expression in esophagus mucosa was 0.461 ± 0.006, demonstrating little to no impact from encoder variability; gene correlation coefficients also had little variation across folds (std = 0.027).

Whilst our histology tile representations were not learnt with the express intent of predicting RNA levels, we demonstrate superior performance against a leading deep learning method, HE2RNA^9^, across the majority of tissues analysed (+0.20 mean *r* score) (Supplementary Fig. 16). Finally, we evaluated the tissue specificity and tissue sharing nature of genes that RNAPath was able to regress well (*r* > 0.5). We see that the majority of these genes are tissue-specific, meaning that they are regressed accurately in a single tissue. However, many genes are regressed equally well in multiple tissues, and recapitulate known tissue relationships and anatomical proximity, such as esophagus muscularis with gastroesophageal junction (shared genes = 837, IoU = 0.35, *P*-value = 1.38 × 10^−6^) and transverse colon with sigmoid colon (shared genes = 1760, IoU = 0.33, *P*-value = 5.83 × 10^−182^). Whilst unrelated tissues such as pituitary gland and omentum share few well regressed genes (shared genes = 11, *P*-value = 0.71). This enrichment reflects the sharing of tissue substructures and cell types between biologically related tissues (Supplementary Fig. 17).

**Fig. 6:**
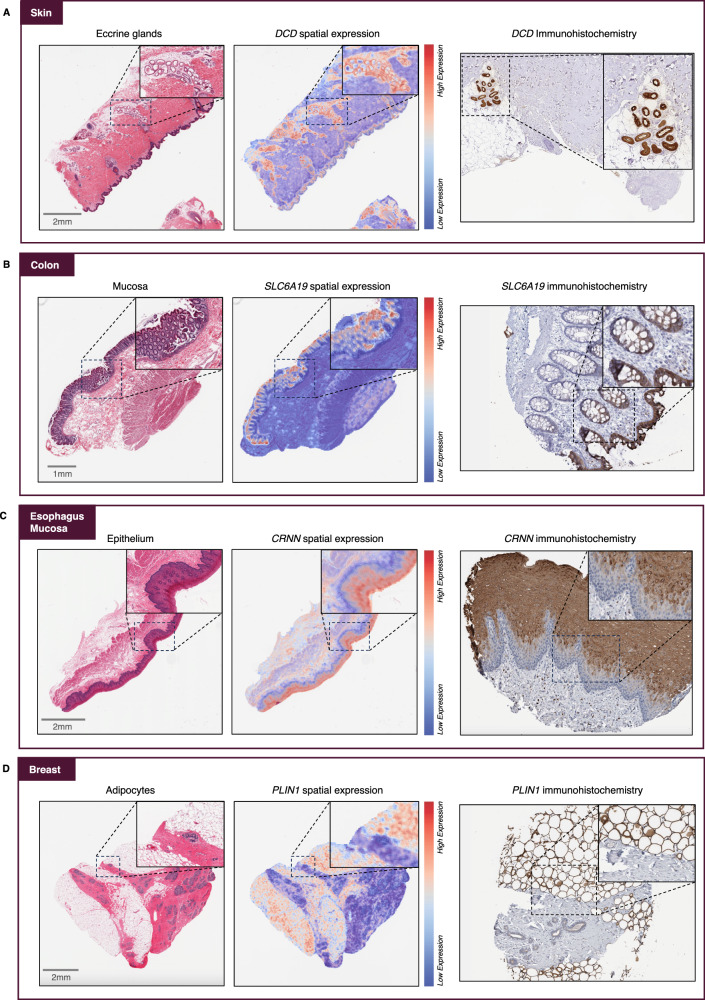
RNAPath predictions of canonical marker gene expression validated by immunohistochemistry (IHC). From left to right: original H&E section from GTEx, RNAPath predicted spatial expression for marker genes (*DCD*: Eccrine sweat glands in skin (**A**); *SLC6A19* in colonic mucosa (**B**); *CRNN* in esophageal mucosal epithelium (**C**); *PLIN1* in breast adipose tissue (**D**); IHC for corresponding protein expression courtesy of Human Protein Atlas^59^. Red corresponds to high expression, blue to low expression. Images available from v23.proteinatlas.org published under a CC BY-SA 3.0 license.

As well as bulk level predictions, RNAPath provides tile level (128 × 128 pixel) expression-morphology predictions which can be used to create spatial expression maps of any specific gene across a histology sample. To validate our spatial predictions in order to use RNAPath for uncovering novel tissue morphology-expression relationships, we sought to examine first the spatial expression of well known marker genes. To do this, we compared the spatial predictions of *PLIN1* (adipocytes), *DCD* (eccrine sweat glands), *CRNN* (mucosal epithelium) and *SLC6A19* (colonic mucosa), genes that are known to be selectively expressed in those tissue substructures, to immunohistochemistry (IHC) in matched tissues from the Human Protein Atlas^37^. We see high concordance between our spatially resolved RNA expression predictions and that of matched antibody staining, validating that we can use RNAPath to draw novel inferences between RNA expression and specific tissue morphology (Fig. 6).

### Spatial expression signatures in tissue substructures and localised tissue pathological features

**Fig. 7:**
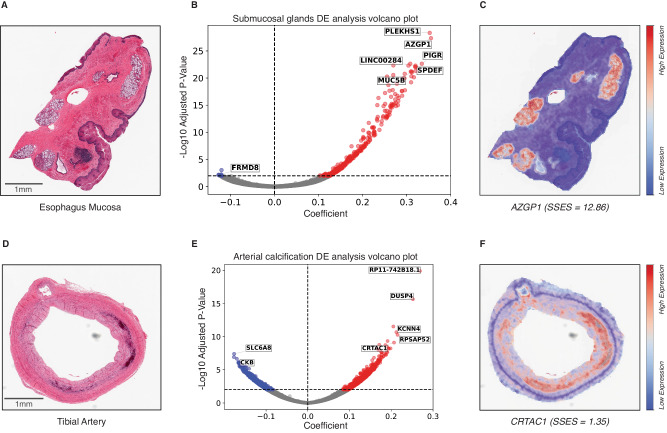
A comparison of differential gene expression analysis and our SSES metric across donors for submucosal gland and arterial calcification proportion. **A**, **D** Esophagus mucosa and tibial artery histology images from GTEx. **B**, **E** Differential expression analysis volcano plot for submucosal gland and arterial calcification proportions (*x*-axis: linear model coefficient, *y*-axis: -Log10 two-sided adjusted *p*-value from t-test after FDR1% correction). Blue corresponds to significantly differentially expressed, downregulated genes, and red corresponds to significantly differentially expressed, upregulated genes. **C**, **F** Considering genes with a positive coefficient (up-regulated), we see that our SSES metric when applied to RNAPath predictions is able to find genes (e.g. *AZGP1* and *CRTAC1*) that are both significant in DE analysis, but are also highly spatially restricted to submucosal glands and calcification foci, respectively.

We sought to detail what genes were specific to individual tissue substructures and pathological features. To do this, we computed an enrichment score for every gene and every quantified tissue substructure and localised pathological feature measured. The enrichment score quantifies the difference of a gene’s predicted spatial expression between a region of interest (ROI) and the whole tissue (see methods). We investigated the relationship between up-regulated genes in our differential expression analysis (e.g. those with a positive coefficient) and our Substructure Specific Enrichment Score (SSES) metric produced by RNAPath. Taking as an example submucosal glands (Fig. 7A–C) and focal inflammation in esophagus mucosa, we identified 311 and 3,471 upregulated genes respectively (FDR1%). By comparing these genes to RNAPath SSE scores, we see that 100% and 91% have SSES > 1 for submucosal glands and inflammation respectively. For tibial artery calcification (Fig. 7D–F), of the 112 upregulated genes in our differential expression results, 64% have SSES > 1, highlighting the difference between differential expression induced changes and genes specific to calcification morphology (see Data and Code Availability to download full summary statistics). Interestingly, *CRTAC1*, a gene we find enriched in areas of calcification (Fig. 7F) was recently independently validated as a spatial calcification and atherosclerosis biomarker^38^.

Finally, as lncRNAs tend to be more tissue specific, we sought to test whether they are also more tissue substructure specific than other gene biotypes. We observe that lncRNAs are predicted more accurately by RNAPath compared to protein-coding genes (0.35 vs 0.32 median *r* score, Supplementary Fig. 18) and are also more likely to be enriched for tissue substructures and pathologies (1.49 vs 1.23 SSES) (see Data and Code Availability to download full summary statistics). These results suggest that RNAPath could be used to further characterise and localise the expression of lncRNAs with unknown function.

In addition to our SSES metric, we computed Moran’s I, a spatial autocorrelation metric commonly used in spatial analysis and more recently in spatial transcriptomics applications^39,40^. A low Moran’s I score indicates a gene is not spatially autocorrelated, with diffuse expression across a tissue section, whilst a high Moran’s I score represents high spatial autocorrelation, with expression restricted to specific substructures, tissue neighbourhoods or pathologies. We computed Moran’s I score for all genes across all tissues, using the spatial predictions of RNAPath, along with the corresponding tile coordinates (see Data and Code Availability to download full summary statistics). The spatial autocorrelation of genes predicted by RNAPath varies significantly, with some genes highly restricted to tissue substructures and others expressed uniformly across the tissue section (Supplementary Fig. 19). Interestingly, as Moran’s I and our spatial predictions are donor specific, we see examples of genes that exhibit subject specific spatial autocorrelation (Supplementary Fig. 20). This analysis highlights how even without substructure or pathology annotations, using RNAPath and derived spatial statistics, it is possible to assign gene expression to specific tissue neighbourhoods.

### External validation in TCGA-BRCA

To test how well our self-supervised representations and RNAPath predictions generalise to a held-out external dataset, we processed all diagnostic histology slides from The Cancer Genome Atlas breast carcinoma study (TCGA-BRCA) and annotated 15 WSI with breast and breast cancer relevant substructures (see methods). As all GTEx slides are acquired with 20× magnification, using 40× TCGA-BRCA WSI demonstrates the generalisation of our approach. Whilst our ViT model was not trained on any oncology data, we see that tile representations of breast-cancer relevant annotations cluster into distinct groups (median silhouette score = 0.159). We also evaluated Imagenet and CTransPath features on the TCGA-BRCA cohort used to externally validate RNAPath. From the qualitative clustering of annotated patches (Supplementary Fig. 21) as well as the median silhouette scores, we see that our model trained using DINO outperforms ImageNet (0.159 vs 0.084, +89%); however, the coefficient is slightly higher for CTransPath than DINO (0.179 vs 0.159), which is explained by the fact that CTransPath has been trained on TCGA. Therefore, our DINO features trained only on GTEx show good generalisation even out of distribution.

Using our kNN approach to segment tissue into its constituent substructures, we see that areas of carcinoma are well segmented from benign tissue (average IoU = 0.723), allowing us to estimate carcinoma proportion variation (0.392 ± 0.212) across all 986 subjects (Supplementary Fig. 22). To validate RNAPath on TCGA-BRCA, we performed two experiments. The first tests RNAPath’s ability to generalise from normal breast to breast carcinoma. Here, we expect the model to be able to predict the expression of genes that are similar across normal breast versus breast carcinoma. With no fine-tuning of our mammary tissue (GTEx) model, we see RNAPath is able to predict known marker genes, for example, *PLIN1* in adipocytes (*r* score = 0.29, *P*-value = 2.27 × 10^−19^) (Supplementary Fig. 23) or breast cancer related genes like *AQP1* (*r* score = 0.32, *P*-value = 1.58 × 10^−23^). However, many other genes that are correctly regressed in the GTEx dataset have a smaller correlation coefficient when evaluated on TCGA-BRCA, like *MEOX2* (*r* score = 0.85 in GTEx, 0.21 in TCGA-BRCA) or *DLK2* (*r* score = 0.84 in GTEx, 0.16 in TCGA-BRCA). This likely reflects that many relations between healthy tissue morphology and gene expression learnt by the model trained in the healthy breast GTEx cohort are not conserved in carcinoma/tumour resection tissue and therefore fine-tuning can be employed to learn disease-specific changes. To account for the fact areas of carcinoma undergo significant morphological and genetic aberrations that profoundly affect gene expression and to assess RNAPath’s ability to predict carcinoma relevant genes, we fine-tuned the model to predict genes from TCGA-BRCA samples using paired RNA-seq, obtaining a correlation score *r* > 0.5 for 351 genes.

**Fig. 8:**
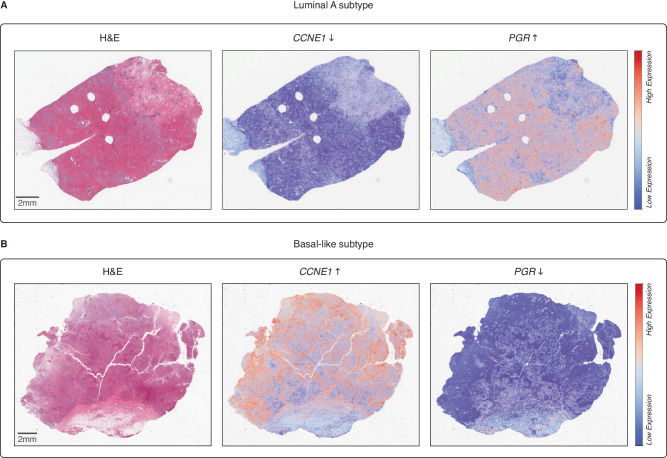
RNAPath predictions of luminal A and Basal-like PAM50 signature genes. RNAPath model predictions on the PAM50 signature, used to stratify breast cancer at molecular level, correlates with the ground truth, with luminal A samples (**A**) having low expression (blue) of *CCNE1 (3.52)* and high expression (red) of *PGR (12.51)* and basal-like samples **B** having high expression of *CCNE1 (10.86)* and low expression of *PGR (2.65)*, as represented in the heatmaps.

Cyclin E1 (*CCNE1)* and Progesterone Receptor (*PGR also known as PR)*, represent two well known breast cancer genes present in the PAM50 signatures that are used to delineate the different molecular subtypes of breast cancer^41^. We see that RNAPath correctly predicts that Luminal-A subtypes are *CCNE1*- *PGR*+, whilst Basal subtypes are *CCNE1*+ *PGR*- (Fig. 8A, B). The median *r* score for the genes in the PAM50 signature is 0.43, and when comparing the fold change of Luminal-A and Basal samples mean gene expression between our predictions, we observe a high correlation (*r* = 0.95).

Moreover, we computed SSES metrics for two genes: *CAMKV* and *MUC2*, which have been previously reported to play an important role in mediating proliferation, apoptosis and metastasis of breast cancer cells^42^. *CAMKV* and *MUC2* are the most enriched genes in areas of carcinoma (SSES = 1.37 and SSES = 1.29 respectively), whilst *NCR1* (synonym: *CD335*) and *CLEC6A*, both selectively expressed in Natural Killer cells (NK-cells), are spatially localised to lymph nodes (SSES = 8.59 and SSES = 6.42 respectively). In conclusion, we demonstrate strong generalisation for both our self-supervised representations and RNAPath gene expression predictions across both normal histology and oncology datasets.

## Discussion

Here we use Vision Transformers (ViT) trained using self-distillation with no labels (DINO) to learn histology image representations from 13,898 WSI across 23 healthy human tissues in 838 donors. By doing so, we are able to demonstrate that representations learnt with no labels are able to identify tissue substructures and pathological features present in WSI, allowing us to represent each donor’s tissue section as a composite proportion of its underlying tissue substructures and any pathology present. By using these proportions, we show profound inter-tissue variability across donors, demonstrating the detection of unannotated pathologies (e.g. calcification), incorrect target tissue assignment (e.g. esophagus mucosa in muscularis samples), contaminate tissue (e.g. adjacent adipose tissue) and how such variability can inflate eQTL tissue sharing estimates. Additionally, we use such proportions to derive and recapitulate known epidemiological links between breast adiposity and age, as well as novel age, sex and BMI associations. Using our derived tissue substructure and pathological feature proportions we characterise differential gene expression signatures and uncover substructure/pathology genetic associations using GWAS. We also demonstrate how such proportions can be used to detect interaction eQTLs, in which tissue substructure and pathological variability across donors drive changes in expression in a genotype-dependent manner.

As our histology representations capture intra- and inter-donor variability in tissue morphology, we propose a multiple instance learning model named RNAPath that can regress RNA expression levels directly from our learnt histology representations, as well as predict the spatial localisation of a given gene’s expression within a tissue section. We validate our expression predictions by showing substantially better performance of RNAPath compared to HE2RNA across a wide range of tissues, and also verify our spatial predictions of known marker genes to ground-truth immunohistochemistry staining.

Our work has several limitations and room for future development. First, we work with 128 × 128 histology image tiles, limiting the resolution of our spatial predictions and segmentations. A natural next step would be to perform cellular or nuclei segmentation and learn self-supervised representations at the single cell-level. Second, whilst we benchmark our SSL representations, it is entirely possible that a model trained on a specific disease cohort (e.g. Inflammatory Bowel Disease, or a specific tumour) would outperform our approach. This is not surprising, as each disease has its own characteristic pathology that a model may only observe in that specific disease context. Third, computational pathology is a fast moving field, with the recent development of large scale foundation models for histology^43,44^. These foundation models, coupled with even newer methodologies for self-supervision could result in even better substructure segmentation and more accurate gene expression prediction and localisation^45^. These developments, alongside more extensive, systematic annotation by pathologists therefore represent a promising future direction. Forth, whilst our discovered GWAS variants recapitulate relationships with known GWAS loci, our genetic analyses are underpowered, both to detect novel GWAS variants and to detect thousands of interaction eQTLs. We believe this will be overcome as larger cohorts with paired histology and genetic data become available, enabling broader discovery but also replication efforts. Fifth, RNAPath is predicting the expression of genes from tissue morphology alone. Many genes exhibit variance attributable to both morphology and non-morphological factors. In these cases, RNAPath will only explain the variance attributable to morphology, whilst sequencing based spatial transcriptomics would explain all variance measurable. Therefore, whilst predicted tile expression correlates to ground truth expression, we would not expect a perfect match between predicted expression scale and ground truth expression. This is to be expected, and still allows for RNAPath to highlight gene-morphology relationships and use this knowledge for many useful downstream inferences.

Finally, whilst we demonstrate and validate RNA-expression prediction from histology this will by definition be limited to genes whose variation influences observable morphological differences in tissue sections. To associate histological variation with intracellular gene expression variation beyond morphology would require spatial transcriptomic assays whose current cost does not scale to large numbers of histology sections, with current endeavours profiling only tens of samples^46^. Therefore, we believe there is still significant value in understanding and characterising more deeply, histological and functional genomic associations at the population level. In summary, as histological archives and pathology workflows become digital, we believe there is substantial opportunity for using self-supervised learning to uncover novel, fundamental biology about tissue structure, function and its variability in a population in both healthy and diseased states.

## Methods

### GTEx Cohort description

All analysis is conducted using data from the Genotype Tissue Expression (GTEx) Consortium^7,25,47^. GTEx consists of a total of 948 post-mortem donors, in which RNA-seq, Whole Genome Sequencing (WGS), and digitised tissue histology have been collected from up to 54 tissue types. For this study, we utilised GTEx v8 considering the overlap between individual donors who had both RNA-seq and matching tissue histology available and tissues with at least 200 donors genotyped. In total, we utilised *N* = 13,898 slides, across 23 tissues.

### RNA-seq normalisation

We used normalised TPM values available in the GTEX v8 release. As the GTEx RNA-seq data is not strand-resolved, we only considered lncRNAs that did not overlap with protein coding loci (see Code Availability). Additionally, on a tissue by tissue basis, we considered only genes that were expressed with TPM > 10 in at least 5% of samples. For prediction, we used log normalised TPM values, *log2*(x + 1). In total, we considered 21,691 genes.

### GTEx Whole Slide Image histology preprocessing

We downloaded all available Whole Slide Image (WSI) histology data from the GTEx portal. All GTEx histology slides were acquired at 20× magnification with an approximate micron per pixel (MPP) scale of 0.494. WSI were first segmented to separate foreground tissue from background, using a previously published U-net architecture trained on 4732 H&E slides^48^. Tissue sections were tiled into image tiles of fixed dimension (128 × 128 pixels, corresponding to approximately 63 × 63 μm^2^) and their coordinates stored in hdf5 files. These tiles were used in all downstream analysis.

### Self-supervised feature learning from histology tiles

After preprocessing, we extracted features from histology tiles building a matrix with as many rows as the number of tiles in the WSI and as many columns as the number of features (i.e. 384). To do this, we trained a small vision transformer (ViT-S, output dimension = 384) using DINO^16^, on 1.7 M histology tiles equally sampled across all the 23 tissues from GTEx that we selected for this study. In this self-supervised training regime there are two networks, a student and a teacher model, sharing the same architecture: the student is provided with global and local augmented crops of the input image, whilst the teacher receives only global crops of the same image. Both models output a *k*-dim probability vector (*k* = 65,536) via a temperature softmax along the feature dimension, which can be thought of as a distribution over latent-classes the model is learning to represent. The student-teacher models are trained by minimising the cross entropy (CE) loss between their output distributions:

1 $${CE}=-{\sum }_{j=1}^{j}\,{y}_{j}\,{\log }_{2}p({x}_{j})$$

This has the desired effect of encouraging the model to learn local-to-global image correspondences. It can be particularly useful in histology, where cells (local crops) may be specific to much larger tissue structures (global crops). We modified the augmentation pipeline of DINO’s global and local crops to better capture the relevant features of histology samples, by adding a random modulation of hematoxylin (H) and eosin (E) channels. Images were stain-normalised before being input to the vision transformer to eliminate any effect due to differential stain intensity^49^.

Once obtained, the matrices of tile representations (K×384, where K is the number of tiles of a WSI) for all samples were stored in a HDF5 together with the corresponding upper-left corner coordinates.

### Weakly supervised segmentation of histology images

The segmentation of histology images into regions of interest identifying substructures or pathological features is fundamental to both extract image derived phenotypes (e.g. size of specific tissue regions) and to compute gene enrichments with RNAPath predictions. To obtain pathology and tissue substructure annotations, we used the following criteria:

1. WSI were randomly selected to extract tiles to include major tissue substructures. For example in arterial tissue: intima, media and adventitia layers. These can be thought of as the tissue’s canonical structures present across the majority of specimens.
2. Pathology notes for tissue specimens were inspected to additionally include donors where incidental structures were present: e.g. presence of hair follicles, or eccrine glands in skin, which are not always present given sampling variability.
3. Pathology notes for specimens were also examined to include incidental pathology findings. For example, atherosclerosis or calcification in arterial samples.

These annotations were performed by C.A.G and independently verified by a trained clinical histopathologist (A.P.L). To perform these annotations, we used QuPath (v0.4.3)^50^ and a groovy script to produce 128 × 128 tiles from each annotated area. All annotation tiles are available for download (see Data Availability). For each tile from an annotated class, we perform a forward pass through our trained ViT-S model, obtaining its 384-dim representation. We can then obtain automatic segmentations of the non-annotated WSI by computing the distance between tile representations from unannotated WSI and by using a k-Nearest Neighbours (k = 200) model fitted on the annotated (tissue-specific) dataset to assign classes. It should be noted, that the kNN model was applied directly to the tile level representations, not the UMAP embeddings, which can vary substantially based on hyperparameter settings. To mitigate cases in which annotated tiles contain multiple classes or individual tile predictions are uncertain, we tile each WSI with overlap, allowing us to average predictions with majority voting. This helps create a continuous prediction and minimise uncertainty at boundaries between two classes (e.g. the media-adventitia boundary in arterial tissue). We stored all segmentations as images and in a dataframe in which the class of each tile is tabulated.

### Assessing associations of tissue substructures with biological and technical covariates

To assess whether a given covariate such as sex, age, BMI or ischemic time was associated with a specific tissue substructure or pathology proportion, we fit linear models in Python using statsmodels (v.0.14.0)^51^. To account for multiple testing, we report adjusted *P*-values based on Bonferroni correction. For effect size estimates, we report the coefficient for the given covariate, conditional on all other covariates as provided by the linear model output.

### Differential expression analysis of image derived phenotypes

Tissue substructure and pathology proportions were computed by counting the number of tiles belonging to each class and normalising by the total number of tissue tiles present in the WSI. However, these phenotypes are compositional as the sum across tissue substructure and pathology proportions within a sample equals one, implying that the measured variables are not independent. This dependence may alter the results of downstream statistical analysis. To address this issue, we transformed the compositional values into pivot coordinates^52,53^. Using these pivot transformed proportions, we fit linear models in Python adjusting for age, sex, BMI, ischemic time and the first 5 genetic PCs.

### GTEx whole genome sequencing (WGS) quality control

The cohort VCF representing whole genome sequencing variant calls was obtained from dbGaP (accession phs000424.v8.p2). All the analyses described here are based on the GTEx v8 analysis freeze dataset containing 838 individuals and 46,569,704 variants. First, we used somalier^54^ to estimate the ancestry of all samples directly from the cohort VCF. Based on somalier estimates, we then selected only the 699 samples of European (EUR) ancestry based on the 1000 G reference populations. Variants were then filtered, retaining only PASS biallelic SNVs. The filtered dataset contained 699 samples and 43,066,451 variants.

To generate a high-quality dataset suitable for GWAS analysis, we further filtered genotypes retaining only those with GQ >= 20 and DP >= 10, and then removed variants with minor allele count <10, HWE test *P*-value < 1 × 10^−30^, or missing call rate > 0.05.

The resulting processed dataset containing 11,527,288 variants was converted to PGEN format and used in step 2 of REGENIE for variant association analyses (see below). Variants in this dataset were further processed to generate a set of independent SNVs to be used in step 1 of REGENIE analysis. First, we filtered out variants with HWE test *P*-value < 1 × 10^−15^, minor allele count <100, or missing call rate > 0.01, and then we applied LD pruning as implemented by plink2 --indep-pairwise method using –indep-pairwise 1000 100 0.5. The final dataset for step 1 included 699 samples and 381,202 variants.

### Genome-wide association analysis (GWAS)

To conduct the GWAS, we used REGENIE v3.2.7^55^ with an automated Nextflow pipeline (v1.8.1) (see Data and Code Availability). The sample size tested varies depending on the phenotype: 691 donors for tibial artery calcification, 479 for coronary artery calcification and 674 for esophagus mucosa inflammation and vascular congestion. For all four phenotypes we tested autosomal variants adjusting for age, sex, BMI, ischemic time and the first five principal components of genetic ancestry. A minMAC filter of 10 was applied in step 2 of the REGENIE pipeline; and the variants with MAF < 5% were excluded from the final analyses. We considered the standard genome-wide significance threshold (*P*-value = 5.0 × 10^−8^), but also examined suggestive hits (*P*-value < 1.0 × 10^−6^). Regional plots, Manhattan plots and quantile-quantile plots were generated with GWASLab (v3.4.21)^56^. All summary statistics are available for download (see Data Availability).

### Interaction eQTL mapping

To perform interaction eQTL analysis, we used TensorQTL (v1.0.8)^57^, an open source package that allows QTL mapping to be executed on GPUs, resulting in ~200–300 fold faster computations compared to the CPU-based implementations. Interaction eQTL analysis requires genotypes, gene expression data and an interaction variate (e.g. a phenotype or environmental factor) for each individual. The statistical model is described by the following equation:

2 $$Y=I+{\beta }_{1}G+{\beta }_{2}P+{\beta }_{3}P\times G+\epsilon$$

where $I$ is the intercept, $G$ the genotype, $P$ the phenotype, $P\times G$ represents the interaction term and $\epsilon$ the residual error. We used the same genotype data as per the GWAS (described above). For the gene expression data we utilised the normalised gene expression matrices and covariates provided by GTEx in the cis-eQTLs section of the open access data.

The covariates include the top five genotype components, PEER factors calculated for the expression matrices, sequencing platform, sequencing protocol, sex, age, BMI and ischemic time. As an interaction term, we used the tissue substructure and pathology proportions transformed into pivot coordinates. The sample size varies across tissue type and depends on the number of genotyped donors with both gene expression and WSI of the histology sample available.

### RNAPath: Multiple instance learning for gene expression regression

RNAPath works as follows: consider a total of $N$ WSIs, each represented as a bag of image tile embeddings, ${X}_{i}\in {R}^{M\times D}$ where $M$ is the number of image tiles for that WSI and $D$ is the embedding dimension of each image tile. $M$, termed the bag size in the MIL literature, is variable across WSI as it depends on the size of the tissue section taken. At the level of each WSI, we have as regression target $Y\in {{R}_{+}}^{G}$, where $G$ is the number of genes selected for the tissue, corresponding to the ${\log }_{2}(x+1)$ TPM values for each gene. The model estimates the gene expression at tile level by *G* independent gene-wise linear regressors applied to tile features. This is a simple linear layer mapping tile representations ${X}_{p}$ to gene level expression predictions, $\hat{{Y}_{p}}$. No fully connected layers or non-linear activation functions were used between layers to ensure that RNAPath tile level predictions are identifiable and interpretable. We subsequently apply a non-linear activation function (ReLU), simply to have non negative tile-level scores. These scores are then averaged to derive a sample-level prediction; mean squared error (MSE) loss between predicted sample-level expression and bulk RNA-seq is computed to train the model (see Code Availability for full implementation).

Formally, the tile-level expression values for a tile $P$ are computed as:

3 $${\widehat{Y}}_{p}=\,{ReLU}({W}^{T}{X}_{P}+b)$$

where $$W\in {R}^{D\times G}$$, $${X}_{p}\in {R}^{D}$$, $$b\in {R}^{G}$$ and $$\widehat{{Y}_{p}\in {{R}^{G}}_{+}}$$.

The sample-level estimate is obtained using the average as an aggregation function of the local predictions:

4 $$\widehat{Y}=\frac{1}{M}{\sum }_{p=1\,}^{M}\widehat{{Y}_{p}}$$

To train RNAPath for each tissue we created a training, validation and test split of 80:10:10, ensuring that tissues from the same individual were present in only one split, to avoid any leakage based on genetic effects shared across tissues. For each sample, we apply dropout both at the bag level (by keeping a random percentage of tiles between 70% and 100% of the total number), and at the level of tile representations (*p* = 0.10) to make the training of RNAPath more stable and to increase robustness to outliers.

We train RNAPath with batch size 1 for a maximum of 200 epochs, using a decaying learning rate scheduler (starting value 1 × 10^−4^); we optimised RNAPath using Adam and a Mean Squared Error (MSE) loss function:

5 $$L=\frac{1}{N}{\sum }_{j=1}^{N}({Y}_{j}-\widehat{{{Y}_{j}}^{2}})$$

We divided the gene set into groups of size ≤ 500, due to memory restrictions. To limit the time taken by the optimization step, we accumulate the gradients over each of these groups and update weights once all the genes for a sample have been regressed. In total, we trained 23 tissue-specific RNAPath models for the regression of gene expression.

We extracted tiles with 75% overlap to have multiple bags of tiles representing the same sample (therefore enlarging the training set with more tile embeddings to be sampled at each iteration, as an additional form of data augmentation) and to achieve fine-grained expression heatmaps by averaging logits in overlapping regions.

### Substructure specific enrichment score (SSES)

To determine whether a given gene expression prediction was spatially restricted, we devised a substructure specific enrichment score (SSES) that computes a ratio between the mean expression in a given area over the total mean expression, using the tile level predictions:

6 $${e}_{i}=\frac{\frac{1}{{{{{{\rm{|}}}}}}j\,\in \,R{{{{{\rm{|}}}}}}}{\sum }_{j\in R}\,\;{p}_{{ij}}}{\frac{1}{{{{{{\rm{|}}}}}}k\,\in \,S{{{{{\rm{|}}}}}}}{\sum }_{k\in S}\,\;{p}_{{ik}}}$$

This produces an SSES metric for each gene $i$, ${e}_{i}$, in which ${e}_{i} \, > \, 1$ represent genes that are spatially enriched for the given ROI (i.e. the average tile-level expression ${p}_{i}$ is higher in the ROI, $R$, than in the whole sample, $S$). Enrichment scores are then averaged across samples, and the final outcome is a matrix reporting the enrichment score for each pair (gene, substructure or localised pathology).

### External validation on TCGA

To validate our study on breast carcinoma specimens from The Cancer Genome Atlas (TCGA-BRCA), we first ran our preprocessing pipeline to separate tissue from background and tile the tissue into 256 × 256 tiles from 986 slides at 40x magnification, in order to maintain the same tissue area per tile (63 × 63 μm^2^) as for the GTEx samples.

Tile features were extracted using the same DINO model trained on 1.7 M GTEx tiles (see section: Self-supervised features learning from histology tiles). We performed substructure segmentation with the same method as for the GTEx samples, just modifying the tile size (256 × 256) and the number of neighbours in the kNN model (k = 50). For the purpose of training and testing our RNAPath model on TCGA samples, we selected the 10,000 genes with highest index of dispersion and used their $\log 2(x+1)$ RPKM as supervisory signal.

### HE2RNA implementation

RNAPath outputs tile-level scores and then averages across tiles for a slide-level aggregated prediction. In our implementation of HE2RNA, we used the authors defined model class, available from their published repository, with our 128 × 128 tiles and self-supervised embeddings. HE2RNA considers bag shapes (number of tiles per WSI) of 8,000 and the number *k* of tiles used in the training step is randomly sampled from the list *L* = [10, 20, 50, 100, 200, 500, 1000, 2000, 5000]. Given that some of our slides have a substantially higher number of tiles, we substituted the absolute numbers in *L* into proportions (*L* / 8000), in order to keep the ratio of tiles used in the training step equal to the original implementation, despite having larger bags. It is worth noting that the original HE2RNA implementation used pre-trained ImageNet feature representations for tiles, which we demonstrate perform significantly worse in representing histological entities. Therefore, this is a conservative comparison in which HE2RNA benefits from using our self-supervised representations.

### Reporting summary

Further information on research design is available in the Nature Portfolio Reporting Summary linked to this article.

### Supplementary information

Supplementary Information

Peer Review File

Reporting Summary

## Data Availability

GTEx V8 data are accessible via an approved dbGAP application (https://www.ncbi.nlm.nih.gov/projects/gap/cgi-bin/study.cgi?study_id=phs000424.v8.p2). DINO ViT and RNAPath Model weights, annotations and GWAS summary statistics for this study are publicly available via: https://github.com/GlastonburyC/RNAPath (10.5281/zenodo.11519629)^58^. Upon publication, summary statistics as well as being available through github will be uploaded to the GWAS catalog. Raw eQTL summary statistics, due to file size (330GB) are available on request (craig.glastonbury@fht.org) and will be made available to requestees within 2 weeks. The original Immunohistochemistry images courtesy of the Human Protein Atlas, used in Fig. 6 can be found here: *PLIN1*: https://v22.proteinatlas.org/ENSG00000166819-PLIN1/tissue/breast *SLC6A19*: https://v22.proteinatlas.org/ENSG00000174358-SLC6A19/tissue/colon *CRNN*: https://v22.proteinatlas.org/ENSG00000143536-CRNN/tissue/esophagus *DCD*: https://v22.proteinatlas.org/ENSG00000161634-DCD/tissue/skin All other data supporting our findings can be found in our supplementary tables.

## Code availability

All code developed as part of this manuscript is publicly available via: https://github.com/GlastonburyC/RNAPath (10.5281/zenodo.11519629)^58^. The nextflow-pipeline used for REGENIE based GWAS is available here: https://github.com/HTGenomeAnalysisUnit/nf-pipeline-regenie.
