# Supplementary material for: Self-supervised learning for characterising histomorphological diversity and spatial RNA expression prediction across 23 human tissue types
Source: Nat Commun. 2024 Jul 13;15:5906. doi: 10.1038/s41467-024-50317-w (PMC11246527; doi:10.1038/s41467-024-50317-w)
Supplement: Supplementary file 1 — Supplementary Information [file 41467_2024_50317_MOESM1_ESM.pdf]

Supplementary Figures

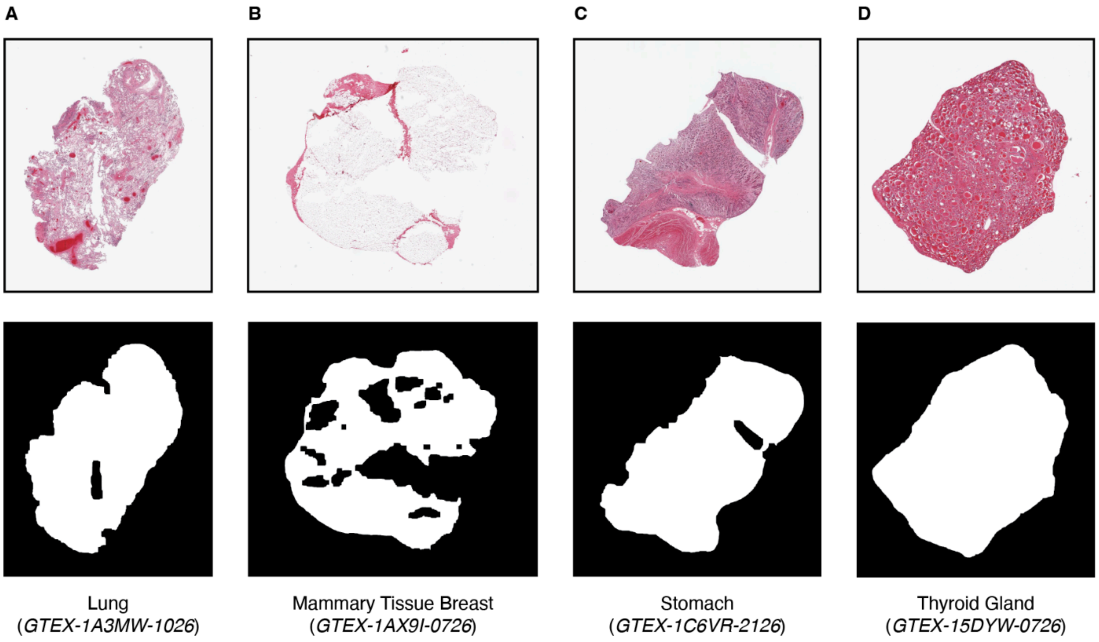

Supplementary Figure 1: Binary segmentation of tissue sections of four HE slides from GTEx tissues: lung (A), breast (B), stomach (C) and thyroid gland (D).

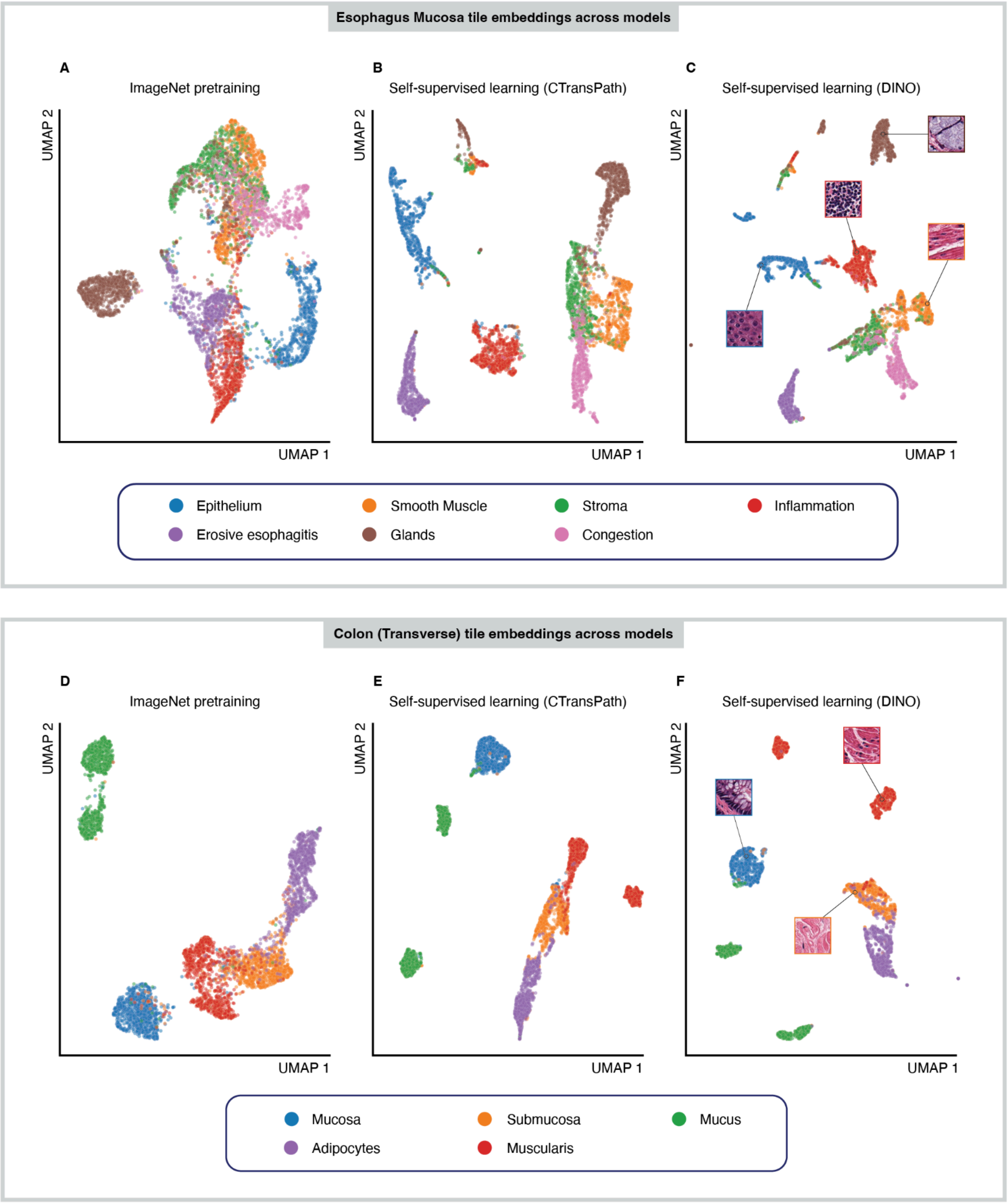

Supplementary Figure 2: UMAP of tile features across models. Esophagus mucosa tile embeddings from ResNet50-ImageNet (A), CTransPath (B) and DINO (C); transverse colon tile embeddings from ResNet50-ImageNet (D) CTransPath (E) and DINO (F). DINO embeddings consistently outperform the other models.

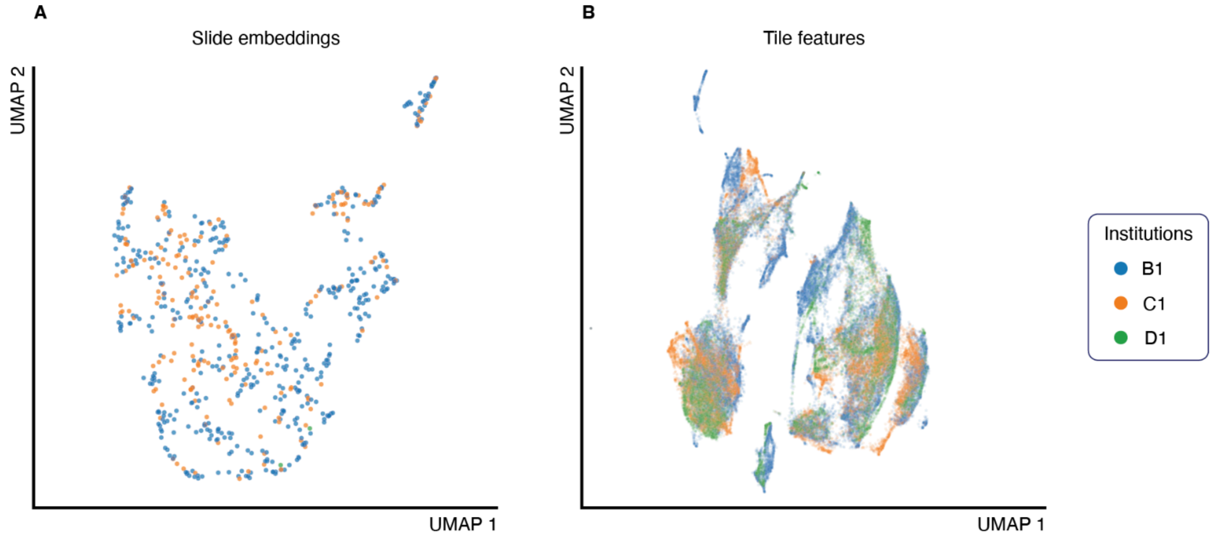

Supplementary Figure 3: UMAP demonstrating lack of residual confounding by institution/centre. (A) Image derived phenotypes for 687 tibial artery samples coloured by different collection centres/institutions. (B) Tile representations from a total of 33 slides, coloured by the institution where the sample was collected. Both IDPs used in downstream analysis and tile representations do not cluster by centre/institution.

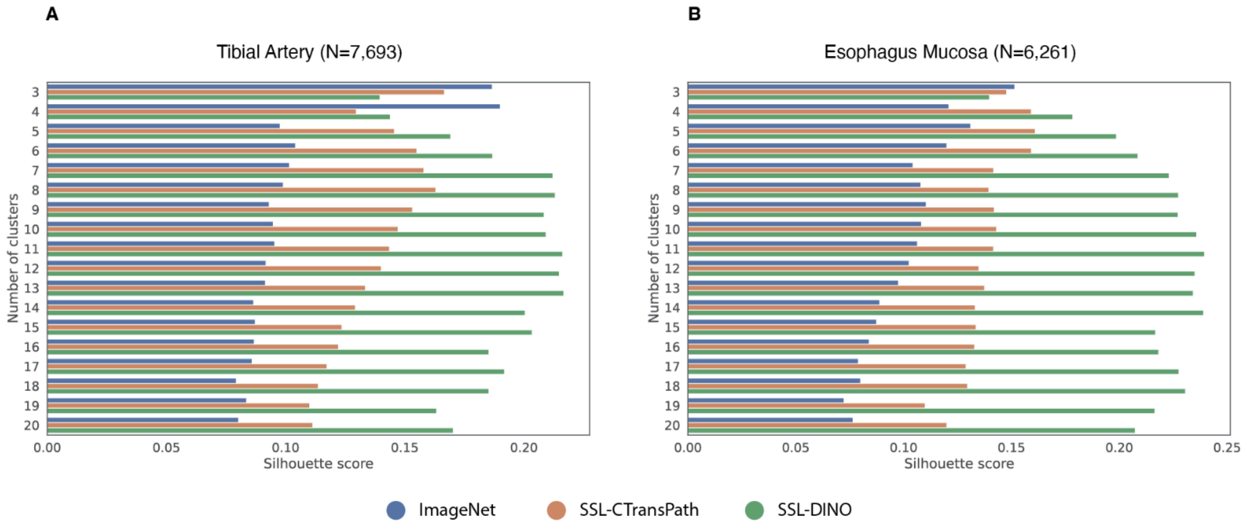

Supplementary Figure 4: Comparison of silhouette scores across several possible cluster values k=[3,20] for tibial artery (A) and esophagus mucosa (B). Models compared include ImageNet pretraining and self-supervised (SSL) learning (CTransPath, DINO). DINO consistently outperforms the other models.

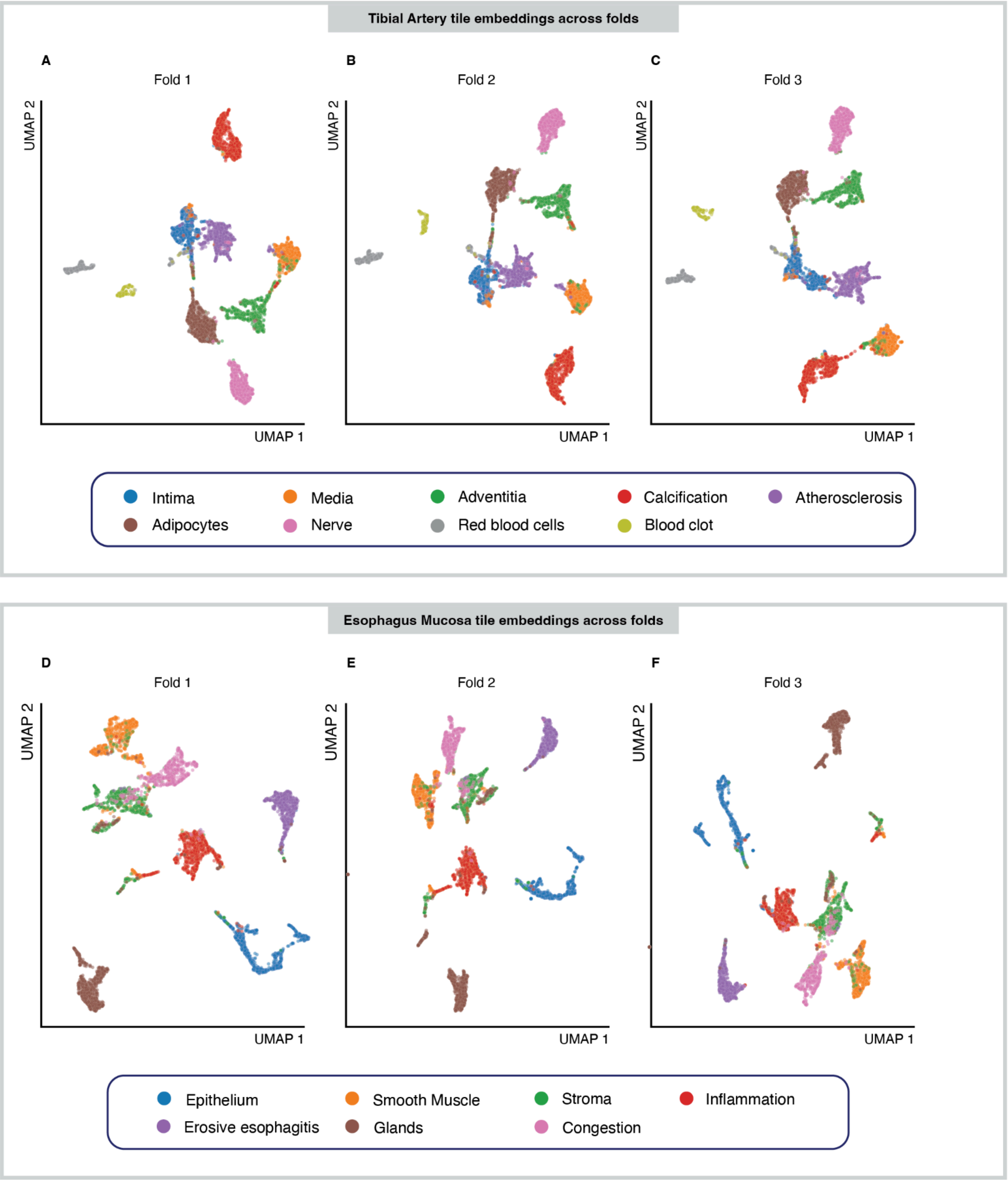

Supplementary Figure 5: UMAP embeddings of tibial artery tile features from DINO model training fold 1 (A), 2 (B) and 3 (C). UMAP embeddings of esophagus mucosa tile features from DINO model training fold 1 (D), 2 (E) and 3 (F).

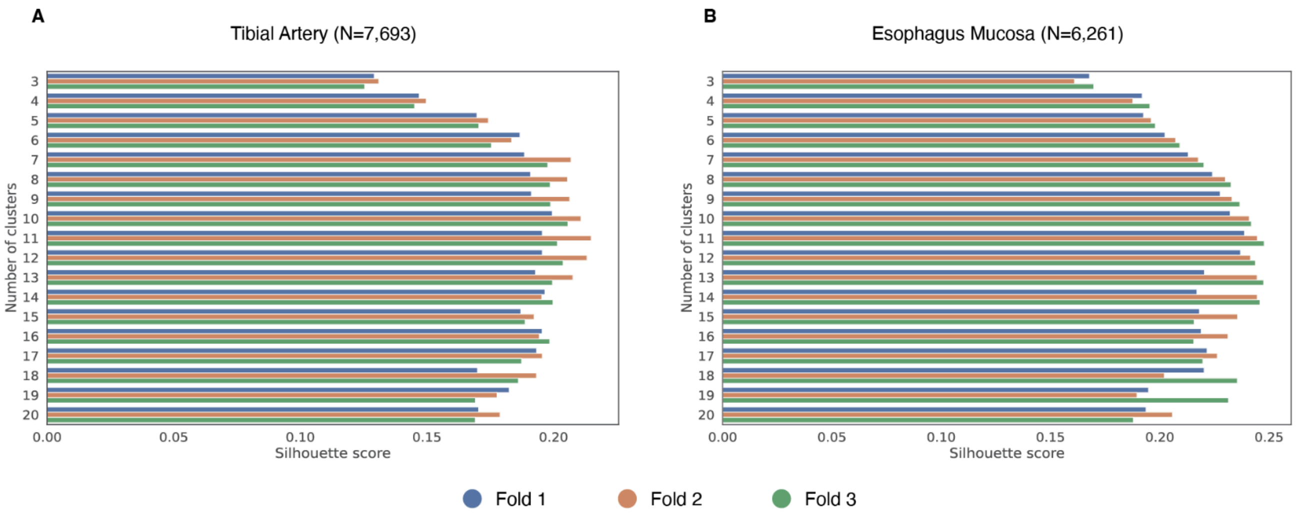

Supplementary Figure 6: Comparison of silhouette scores across several possible cluster values k=[3,20] for tibial artery (A) and esophagus mucosa (B). Models are compared by DINO model training fold; the plots clearly show little difference in clustering across the three folds.

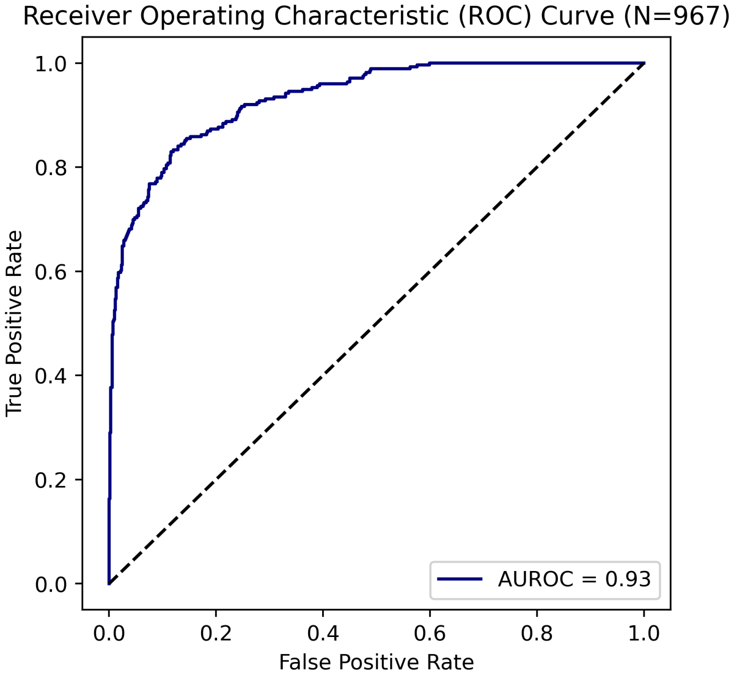

Supplementary Figure 7: ROC curve TPR/FPR for 967 tibial artery samples pathology notes vs calcification level prediction.

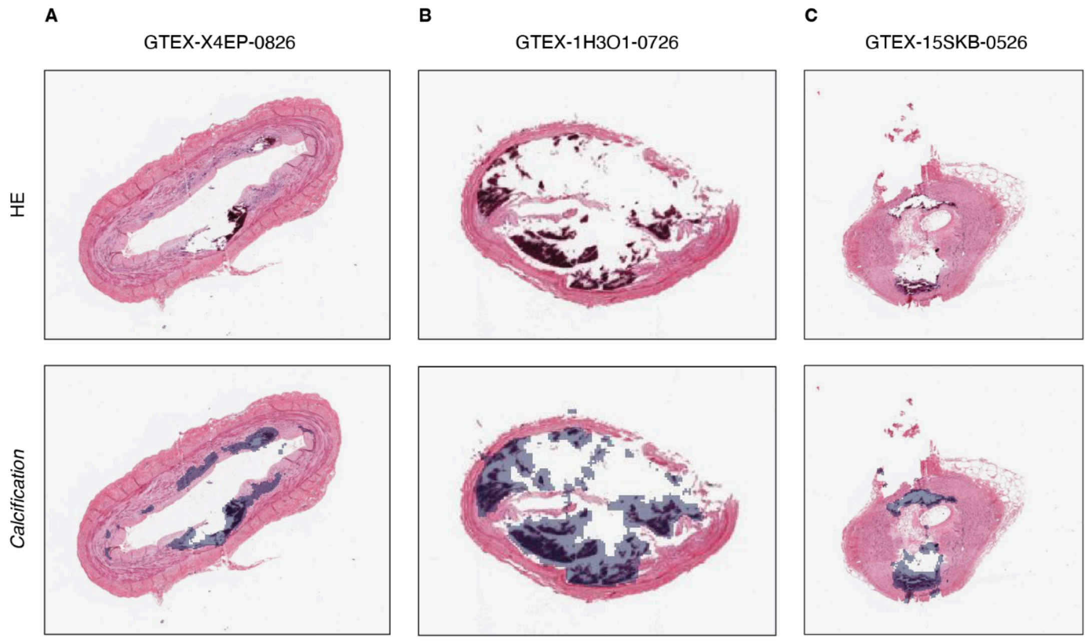

Supplementary Figure 8: Samples with unreported tibial artery calcification. GTEX-X4EP-0826 (A), GTEX-1H3O1-0726 (B) and GTEX-15SKB-0526 (C) were detected to contain calcification, but this was not outlined in the pathologist notes.

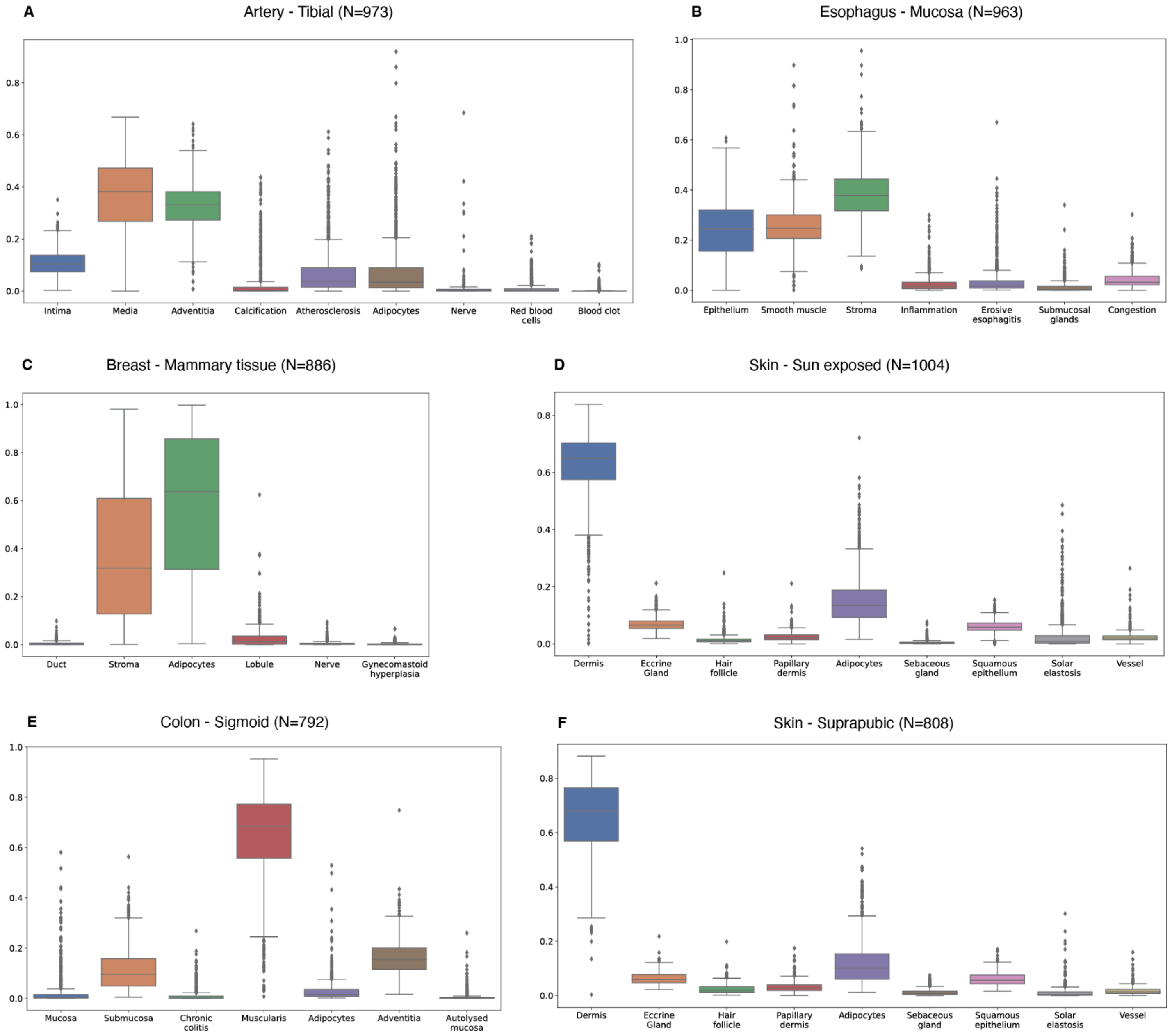

Supplementary Figure 9: Variability of derived phenotypes (tissue substructures and localised pathologies) across GTEx donors from 6 example tissues: tibial artery (A), esophagus mucosa (B), breast (C), sun exposed skin (D), sigmoid colon (E) and suprapubic skin (F). The boxes extend from the lower to the upper quartile; the whiskers range is (Q1 - 1.5×IQR, Q3 + 1.5×IQR).

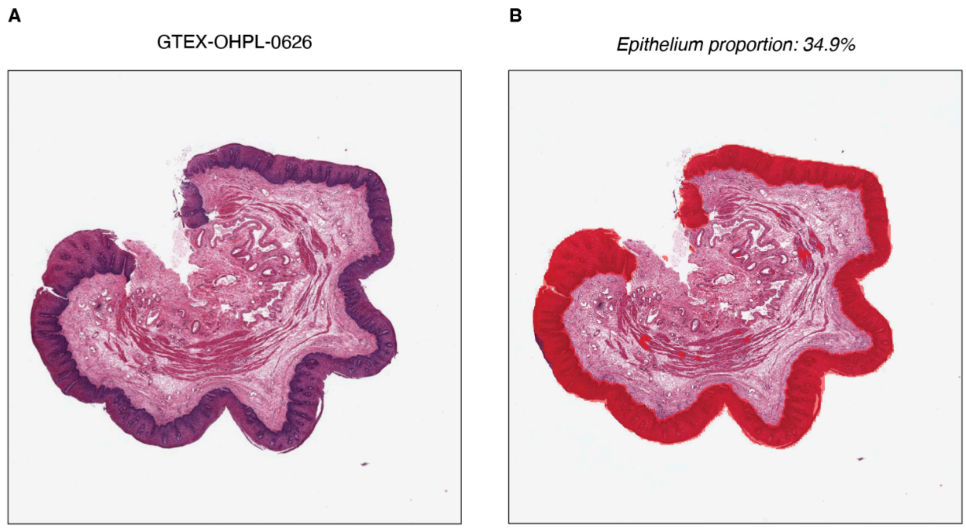

Supplementary Figure 10: Esophagus muscularis sample (A) with large mucosal epithelium proportion that has not been trimmed and its corresponding segmentation (B).

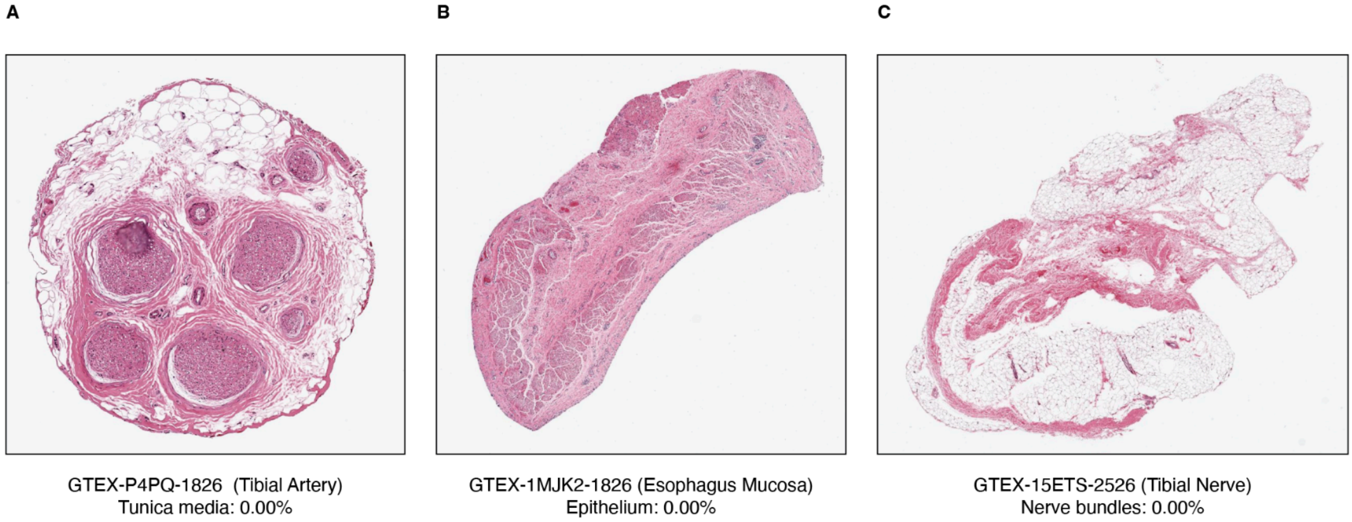

Supplementary Figure 11: GTEx outliers in the tissue proportion distributions. (A) Sample stored as tibial artery just having peripheral nerve. (B) Esophagus mucosa histology without mucosal epithelium. (C) Tibial nerve sample with no nerve bundle; unknown provenance, not GTEx target according to the pathology notes.

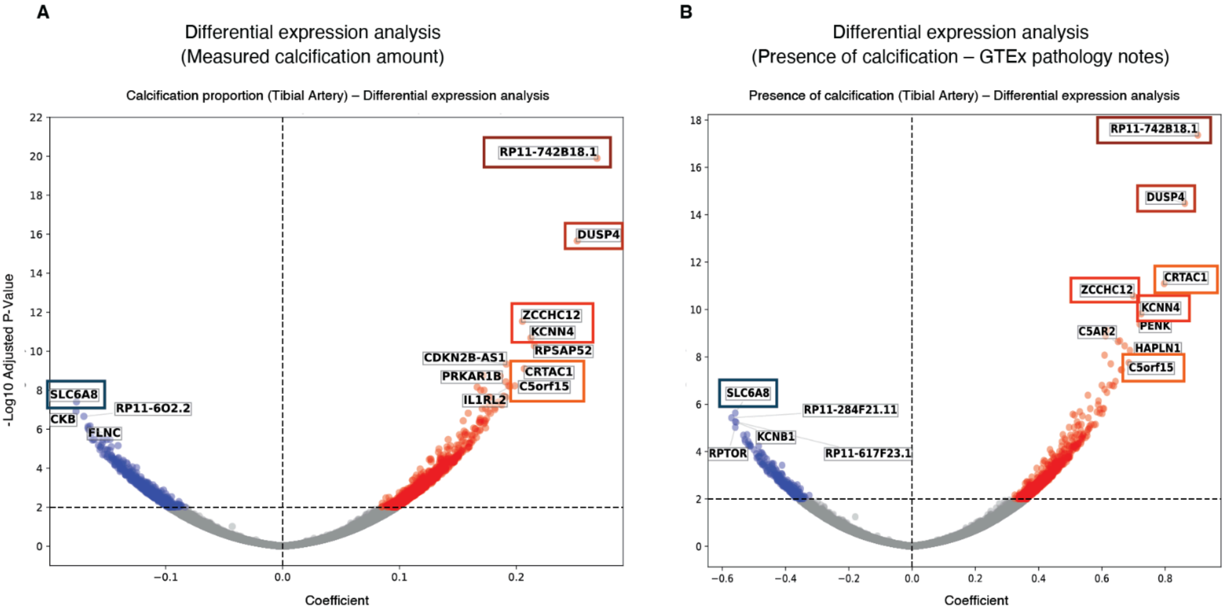

Supplementary Figure 12: Differential expression analysis for tibial artery calcification using the image-derived phenotypes (A) and the GTEx pathology reports (B). The top low-expressed (blue) and high-expressed (red) genes are shared by both analysis; however, when using the continuous values of the calcified tissue proportion detected in the histology sample, we both have increased statistical power and find more DE genes, likely the ones associated with amount of calcification rather than just its presence.

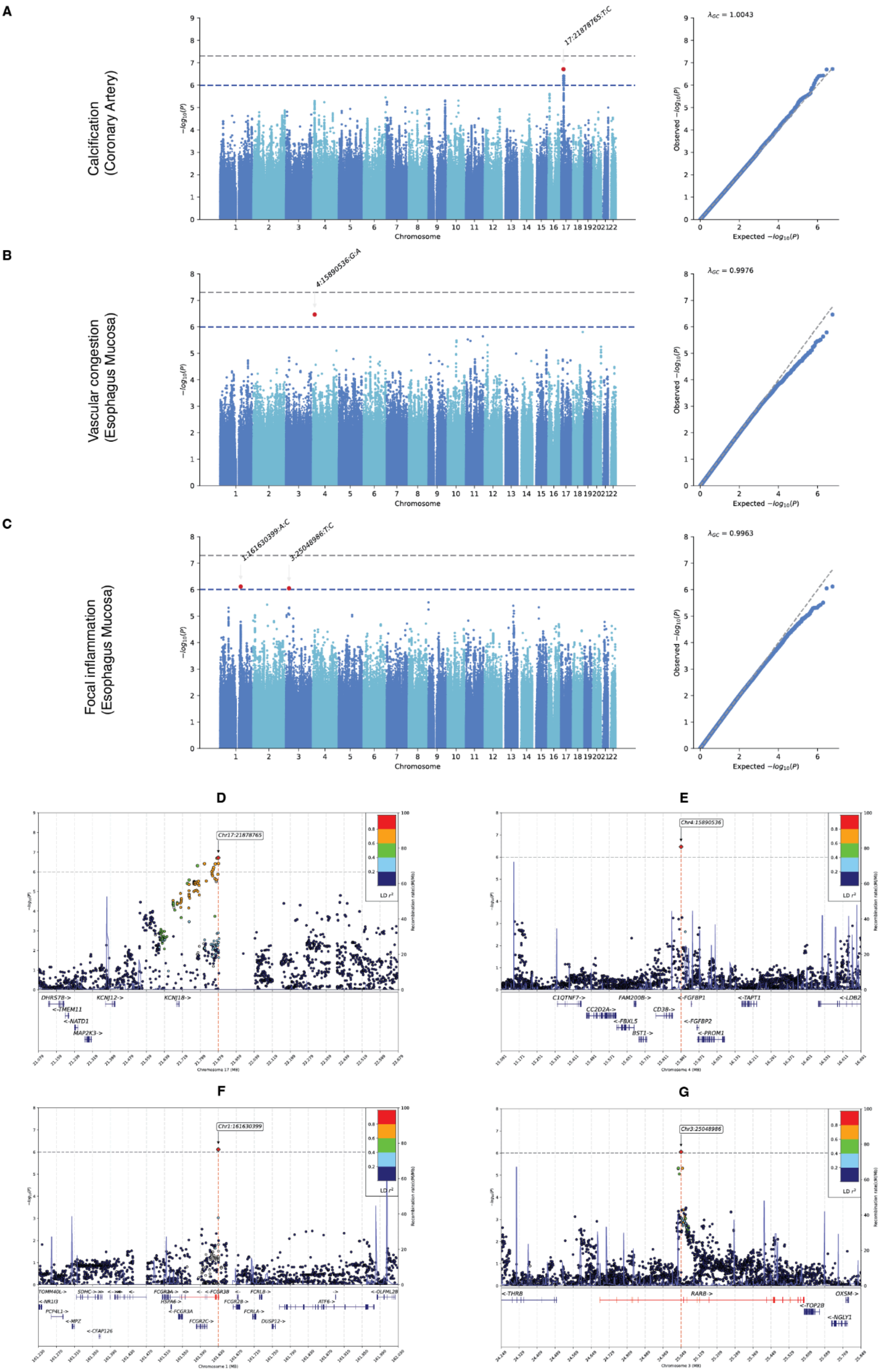

A
Calcification
(Coronary Artery)
17:21878765:T:C
λ_{GC} = 1.0043
B
Vascular congestion
(Esophagus Mucosa)
4:15890536:G:A
λ_{GC} = 0.9976
C
Focal inflammation
(Esophagus Mucosa)
1:161630399:A:C
3:25048986:T:C
λ_{GC} = 0.9963
$-log_{10}(P)$
Chromosome
Observed $-log_{10}(P)$
Expected $-log_{10}(P)$
D
Chr17:21878765
DHRS7B->
<-TMEM11
<-NATD1
MAP2K3->
KCNJ12->
KCNJ18->
Chromosome 17 (Mb)
E
Chr4:15890536
C1QTNF7->
CC2D2A->
FAM200B->
<-FBXL5
BST1->
CD38->
<-FGFBP1
<-FGFBP2
<-PROM1
<-TAPT1
<-LDB2
Chromosome 4 (Mb)
F
Chr1:161630399
TOMM40L->
<-NR1I3
PCP4L1->
<-MPZ
<-CFAP126
SDHC->
FCGR2A->
HSPA6->
<-FCGR3A
FCGR2C->
<-FCGR3B
FCGR2B->
FCRLA->
FCRLB->
DUSP12->
ATF6->
<-OLFML2B
Chromosome 1 (Mb)
G
Chr3:25048986
<-THRB
RARB->
<-TOP2B
OXSM->
<-NGLY1
Chromosome 3 (Mb)
LD r^2
Recombination rate (cM/Mb)

Supplementary Figure 13: Manhattan, QQ (top) and locus zoom (bottom) plots for four variants associated with calcification (A, D) in coronary artery, vascular congestion (B, E) and focal inflammation (C, F, G) in esophagus mucosa.

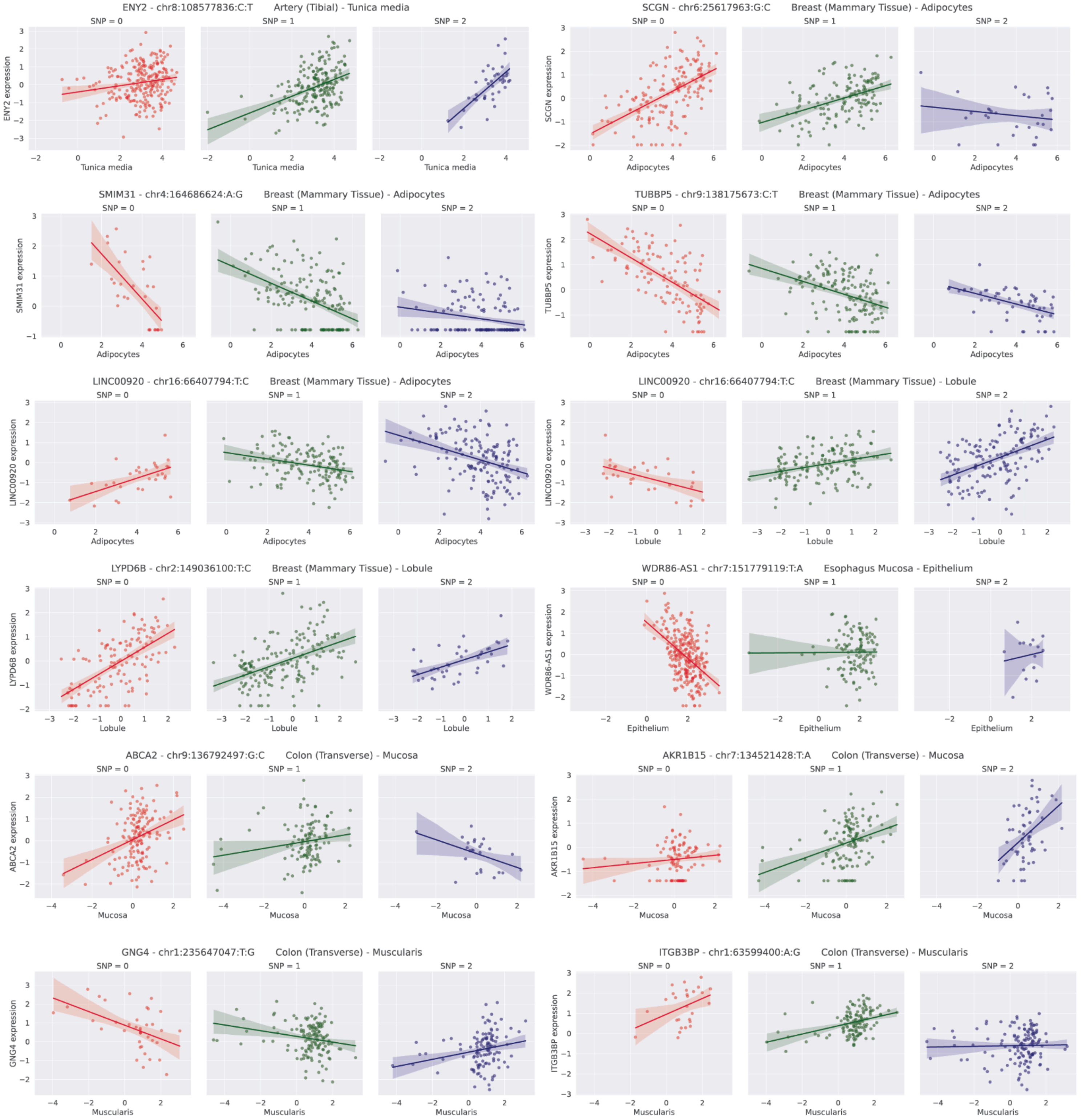

Supplementary Figure 14: Example interaction eQTL pivot plots for several tissue substructures and pathologies.

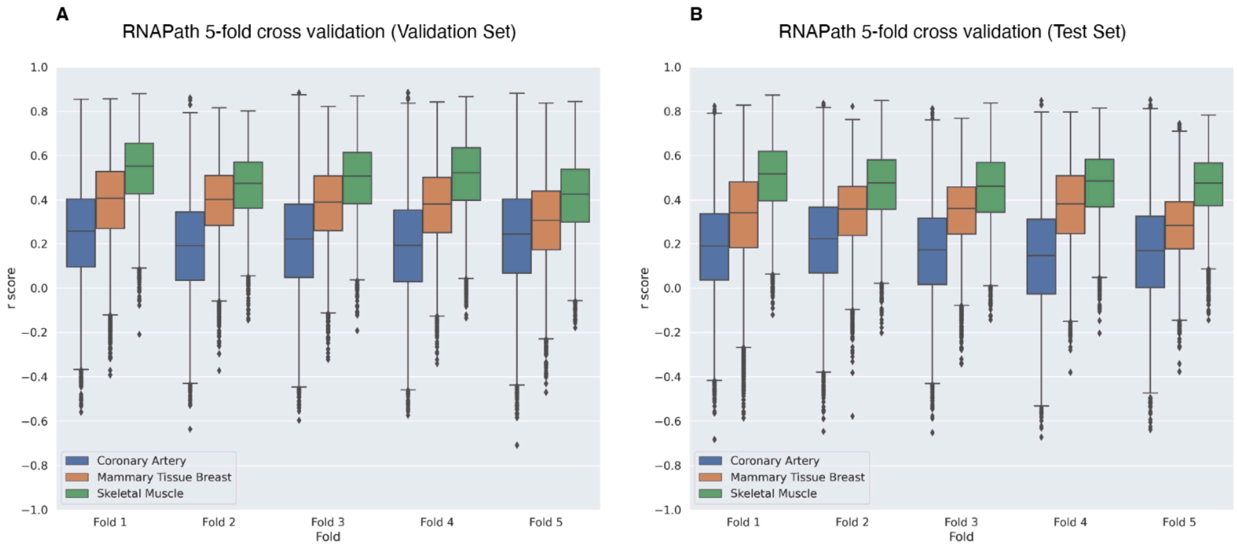

Supplementary Figure 15: RNAPath 5-fold cross validation. Validation (A) and test (B) set results across the smallest sample size (Coronary Artery), median sample size (Breast) and the largest sample size (Skeletal Muscle). The box extends from the lower to the upper quartile; the whiskers range is (Q1 - 1.5×IQR, Q3 + 1.5×IQR). The number of samples in the validation and test sets per tissue are detailed in Supplementary Table 4. As expected, an increase in sample size corresponds to more accurate and robust regression of gene expression.

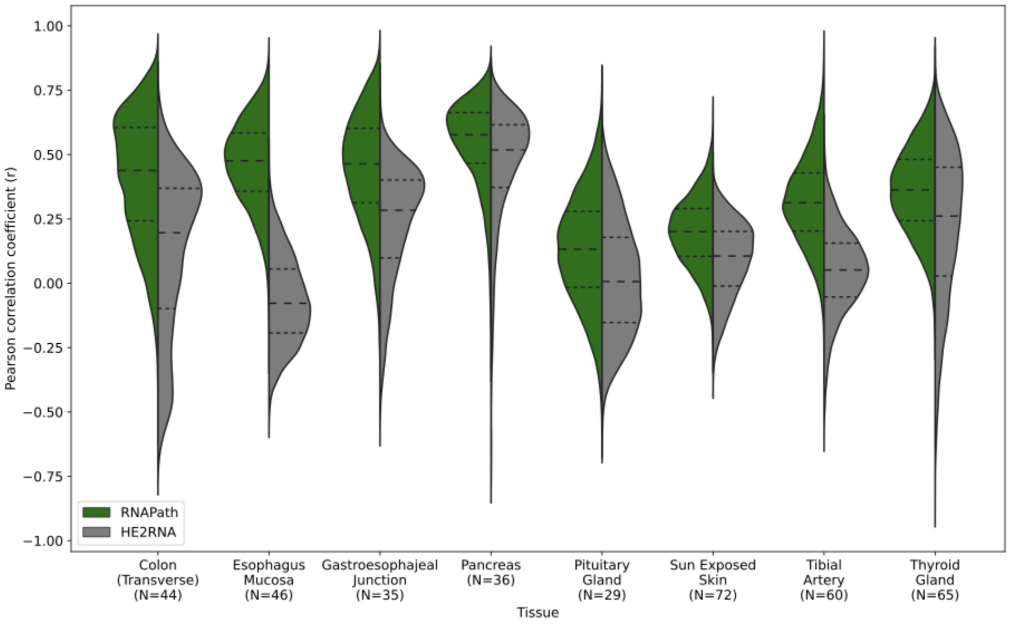

Supplementary Figure 16: Comparison of RNA-expression prediction accuracy by RNAPath versus HE2RNA across 8 example tissues. Distribution quartiles are explicitly

reported in each violinplot. The average number of genes regressed per tissue is 11,268. Tissue names are outlined on the x-axis, together with the number of individuals in each test set.

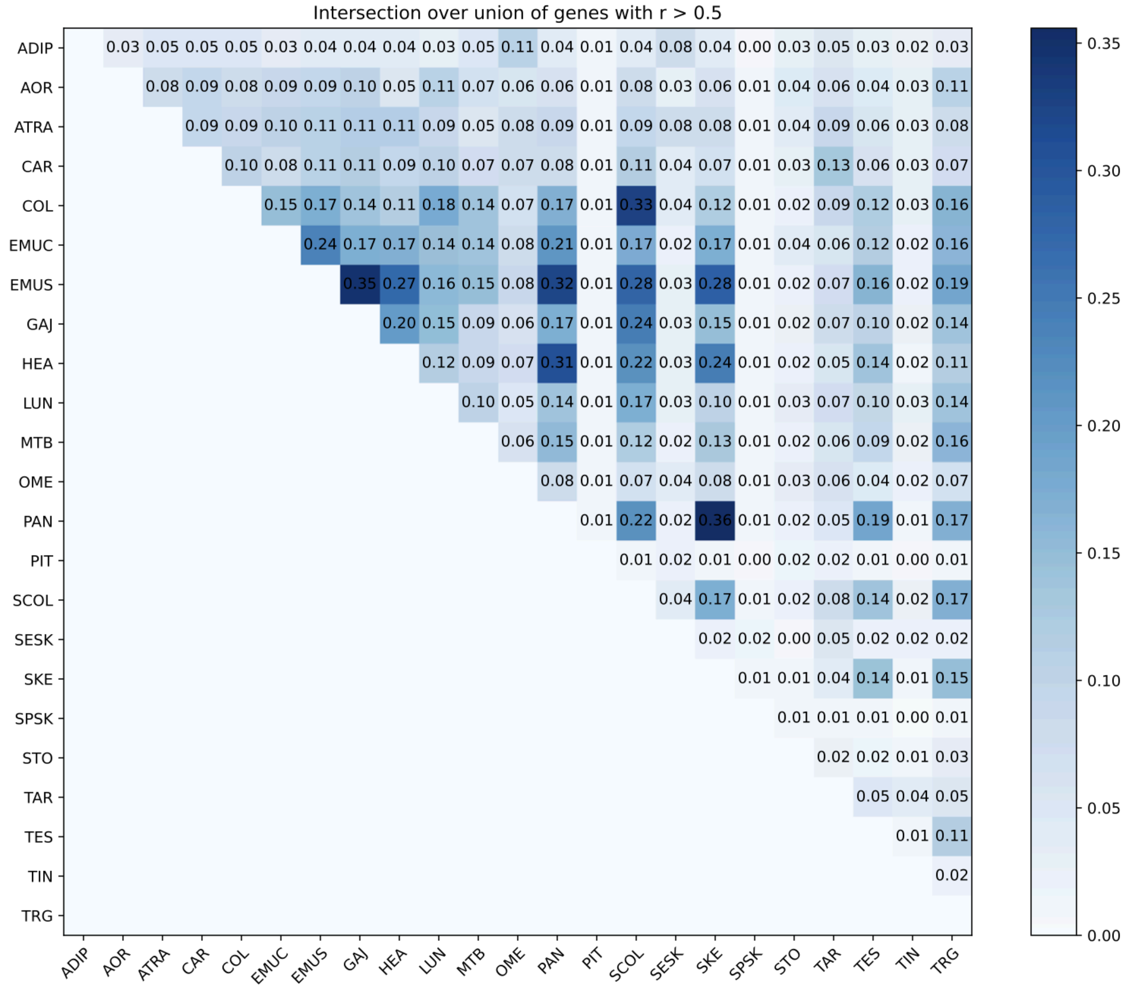

Supplementary Figure 17: Intersection over union (IoU) of genes regressed with correlation r score > 0.5 in the validation set. The number of samples in the validation set per tissue are detailed in Supplementary Table 4. The couple of tissues sharing the majority of well-predicted genes are pancreas (PAN) with skeletal muscle (SKE), esophagus muscularis (EMUS) with gastroesophageal junction (GAJ) and transverse colon (COL) with sigmoid colon (SCOL).

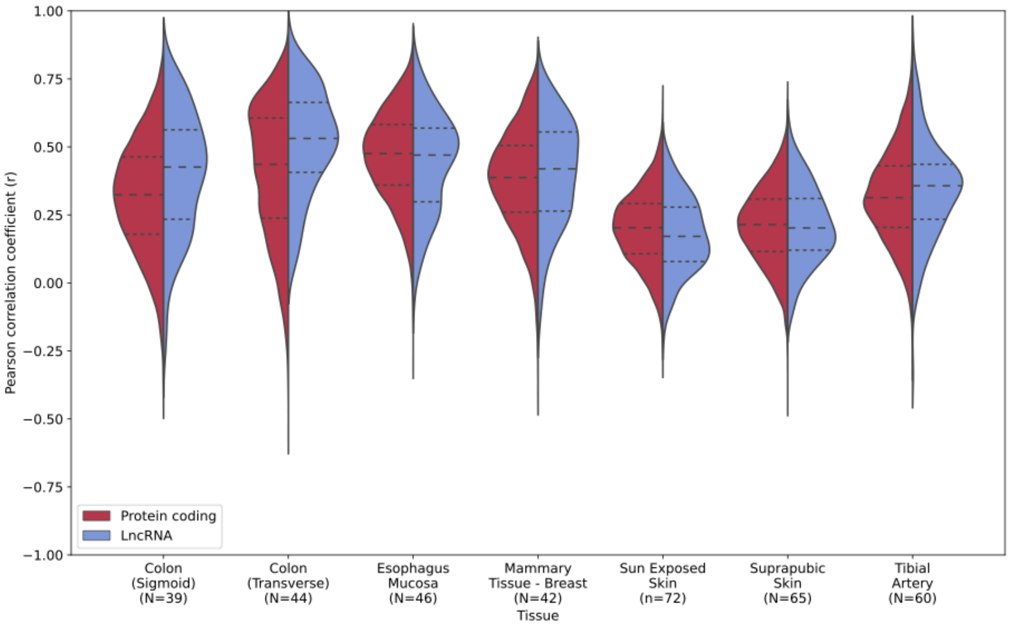

Supplementary Figure 18: Comparison of RNA-expression prediction accuracy between protein coding genes and long non-coding RNAs across the 7 annotated tissues. Distribution quartiles are explicitly reported in each violinplot. The average number of genes regressed per tissue is 10,651 (protein coding) and 178 (lncRNAs). Tissue names are outlined on the x-axis, together with the number of individuals in each test set.

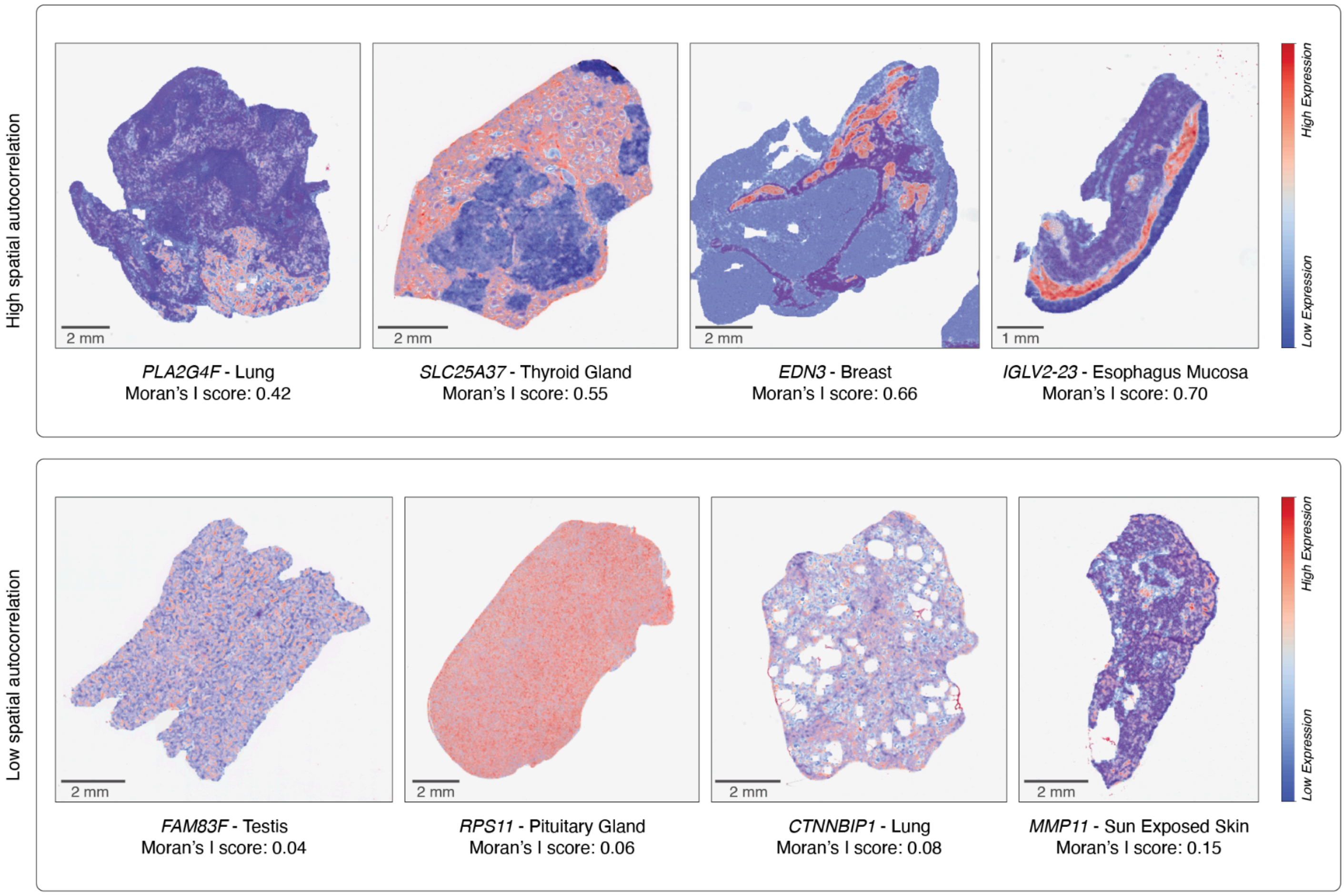

Supplementary Figure 19: Genes with high (top row) and low (bottom row) spatial autocorrelation. For example, *IGLV2-23*, a gene whose expression is specific to B-cells, has high spatial autocorrelation and its expression is spatially restricted to regions of focal inflammation below the mucosa. Whereas *RPS11*, a ribosomal subunit, is constitutively expressed across the pituitary gland with low spatial autocorrelation.

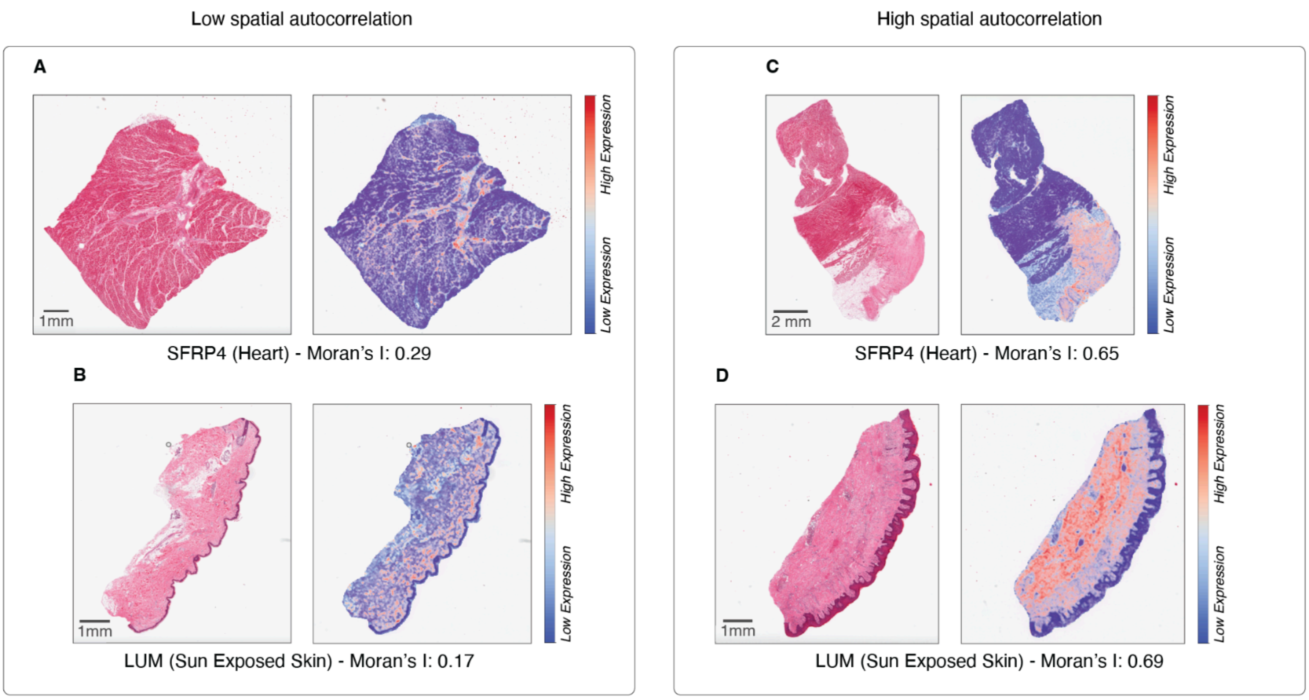

Supplementary Figure 20: Examples of genes which exhibit donor-specific spatial autocorrelation: *SFRP4* in heart (A, C) and *LUM* in sun exposed skin (B, D).

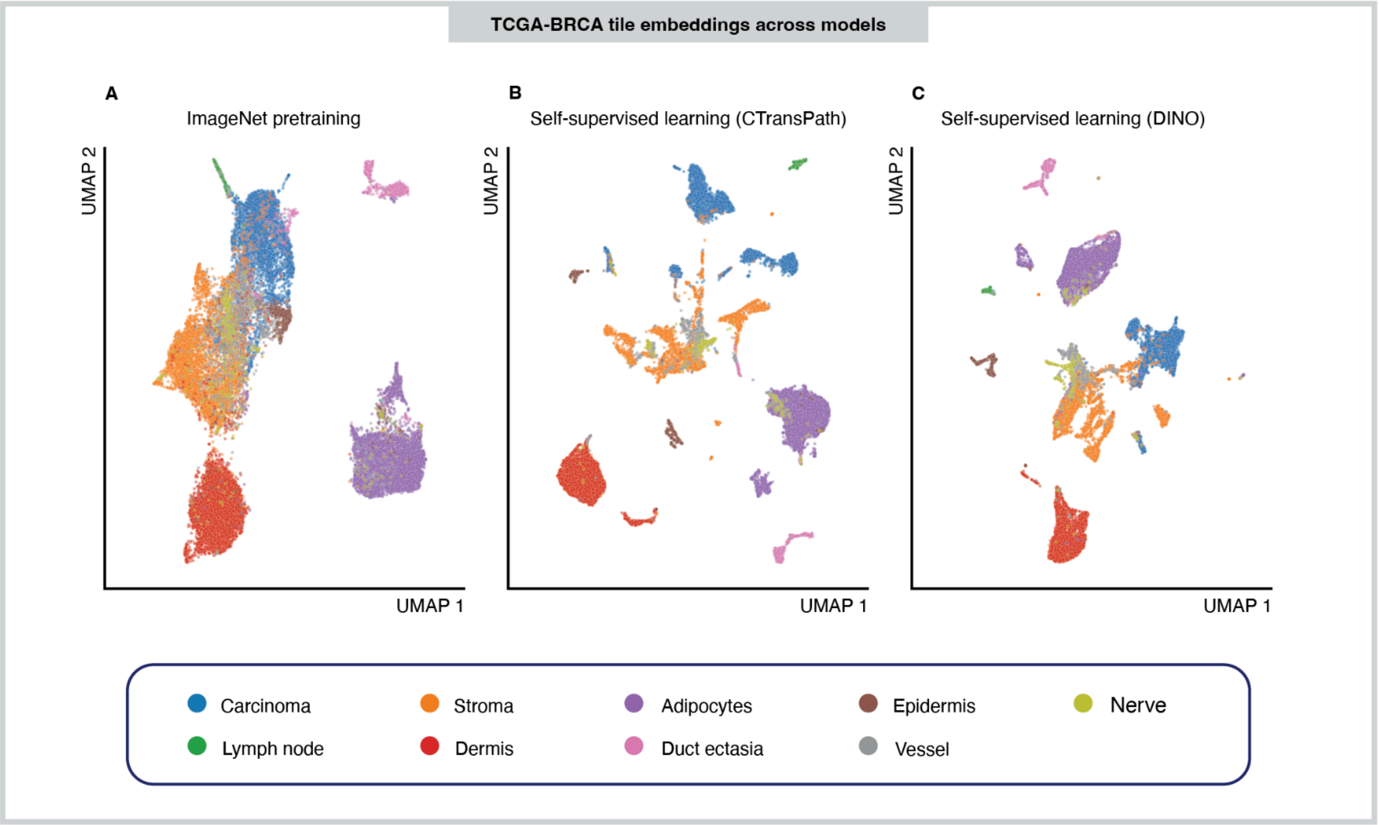

Supplementary Figure 21: UMAP embeddings of BRCA tile features from ResNet50-ImageNet (A), CTransPath (B) and the DINO model trained on GTEx (C).

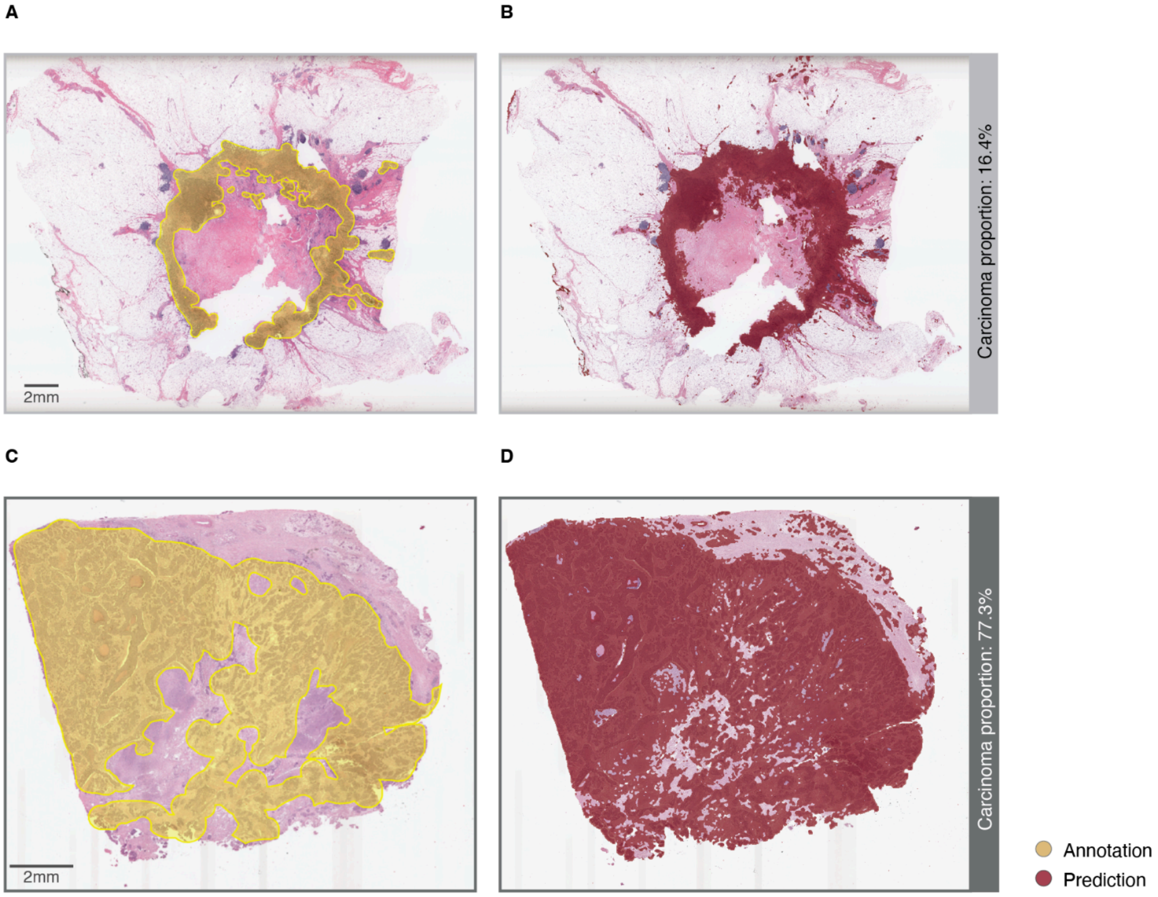

Supplementary Figure 22: Carcinoma area annotation (A, C) and prediction (B, D) for two TCGA-BRCA samples.

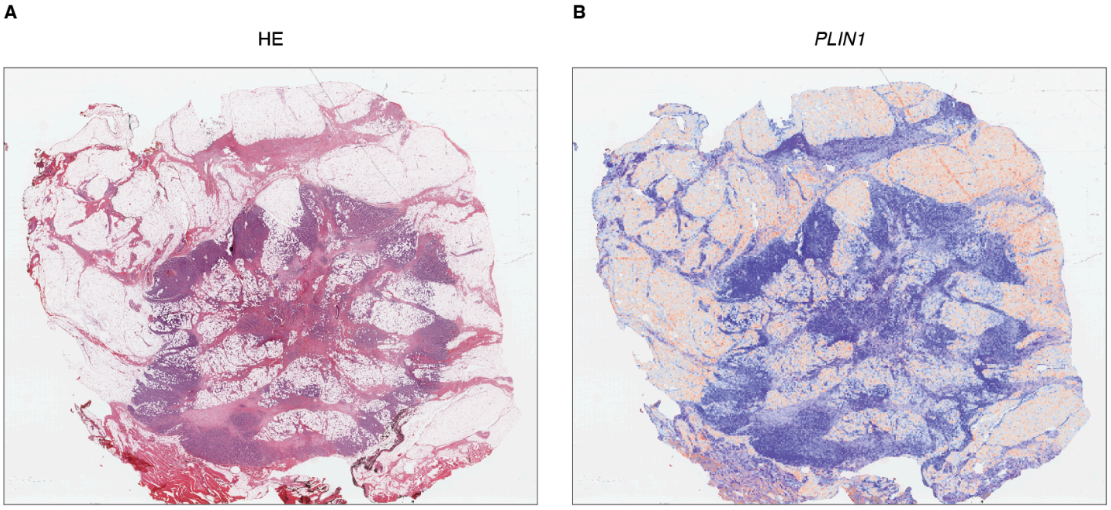

Supplementary Figure 23: A TCGA-BRCA histology (A) with its corresponding heatmap of *PLIN1* (B), from the RNAPath model trained on GTEx breast tissue samples. *PLIN1* is a marker of adipocytes, and the heatmap clearly shows high expression in adipose tissue.

Supplementary Tables

Tissue	Image Derived Phenotype	Annotated Tiles	Slides	Accuracy (tile-level split)	Accuracy (slide-level split)
Artery (Tibial)	Tunica intima	1,038	5	0.925 ± 0.023	0.917
	Tunica media	5,365	5	0.920 ± 0.024	0.927
	Tunica adventitia	3,172	5	0.900 ± 0.038	0.948
	Calcification	1,058	4	0.939 ± 0.014	0.906
	Atherosclerosis	1,780	2	0.943 ± 0.019	0.727
	Adipocytes	5,297	7	0.964 ± 0.020	0.950
	Nerve	1,376	2	0.982 ± 0.015	0.870
	Red blood cells	465	2	0.951 ± 0.032	0.229
	Blood clot	228	1	0.908 ± 0.060	-
Colon (Transverse)	Mucosa	2,680	2	0.979 ± 0.018	0.967
	Submucosa	4,030	3	0.960 ± 0.024	0.966
	Mucus	1,215	3	0.987 ± 0.012	0.965
	Muscularis	2,752	2	0.941 ± 0.019	0.795
	Adipocytes	953	3	0.914 ± 0.021	0.582
Colon (Sigmoid)	Mucosa	2,692	3	0.945 ± 0.021	0.927
	Submucosa	2,628	3	0.702 ± 0.036	0.329
	Chronic colitis	541	1	0.919 ± 0.029	-
	Muscularis	5,545	5	0.972 ± 0.009	0.984
	Adipocytes	823	2	0.855 ± 0.046	0.642
	Adventitia	1,030	1	0.834 ± 0.031	-
	Autolysed mucosa	196	1	0.816 ± 0.092	-
Esophagus Mucosa	Epithelium	1,530	5	0.919 ± 0.038	0.917
	Smooth muscle	2,963	4	0.873 ± 0.045	0.846
	Stroma	4,328	5	0.851 ± 0.038	0.856
	Inflammation	884	5	0.937 ± 0.027	0.933
	Erosive esophagitis	747	1	0.973 ± 0.026	-
	Submucosal gland	1,822	3	0.923 ± 0.027	0.940
	Congestion	630	1	0.886 ± 0.027	-
Mammary Tissue Breast	Duct	471	3	0.495 ± 0.078	0.316
	Stroma	11,399	8	0.963 ± 0.021	0.969
	Adipocytes	12,820	7	0.976 ± 0.011	0.966
	Lobule	803	3	0.940 ± 0.019	0.965
	Nerve	328	3	0.829 ± 0.039	0.547
	Gynecomastoid hyperplasia	262	3	0.771 ± 0.097	0.554
	Dermis	1,464	2	0.973 ± 0.018	0.874

Skin	Eccrine gland	763	2	0.885 ± 0.038	0.956
	Hair follicle	960	2	0.850 ± 0.034	0.612
	Papillary dermis	218	2	0.288 ± 0.066	0.047
	Adipocytes	2,727	2	0.903 ± 0.028	0.957
	Sebaceous gland	653	2	0.847 ± 0.044	0.898
	Squamous epithelium	470	2	0.819 ± 0.067	0.423
	Solar elastosis	178	2	0.820 ± 0.084	0.576
	Vessel	630	1	0.835 ± 0.049	0.794

Supplementary Table 1: Accuracy of all the annotated image derived phenotypes across 6 tissues and their cross validation variability using a k-nearest neighbours model on DINO tile features.

**Tissue**	**Image Derived Phenotype**	**Accuracy (tile-level split)**	
		**ImageNet**	**CTransPath**
Artery (Tibial)	Tunica intima	0.640 ± 0.051	0.882 ± 0.026
	Tunica media	0.867 ± 0.031	0.926 ± 0.031
	Tunica adventitia	0.785 ± 0.017	0.866 ± 0.033
	Calcification	0.707 ± 0.067	0.901 ± 0.022
	Atherosclerosis	0.692 ± 0.033	0.888 ± 0.039
	Adipocytes	0.851 ± 0.030	0.956 ± 0.022
	Nerve	0.873 ± 0.034	0.917 ± 0.031
	Red blood cells	0.847 ± 0.073	0.873 ± 0.054
	Blood clot	0.551 ± 0.095	0.864 ± 0.085
Colon (Transverse)	Mucosa	0.914 ± 0.025	0.955 ± 0.025
	Submucosa	0.894 ± 0.030	0.926 ± 0.024
	Mucus	0.952 ± 0.013	0.983 ± 0.014
	Muscularis	0.904 ± 0.032	0.937 ± 0.024
	Adipocytes	0.870 ± 0.040	0.942 ± 0.015
Esophagus Mucosa	Epithelium	0.788 ± 0.041	0.902 ± 0.032
	Smooth muscle	0.845 ± 0.028	0.871 ± 0.023
	Stroma	0.768 ± 0.038	0.815 ± 0.047
	Inflammation	0.845 ± 0.028	0.880 ± 0.030
	Erosive esophagitis	0.884 ± 0.051	0.991 ± 0.011
	Submucosal gland	0.745 ± 0.047	0.888 ± 0.032
	Autolysed mucosa	0.624 ± 0.063	0.870 ± 0.039

Supplementary Table 2: Accuracy of all the annotated image derived phenotypes across 3 tissues and their cross validation variability using k-nearest neighbours models on ResNet50-ImageNet and CTransPath tile features.

Tissue	Image Derived Phenotype	Mean	Standard Deviation	Min	Max
Artery (Tibial)	Tunica intima	0.11	0.05	0.00	0.35
	Tunica media	0.36	0.14	0.00	0.67
	Tunica adventitia	0.33	0.09	0.01	0.64
	Calcification	0.03	0.07	0.00	0.44
	Atherosclerosis	0.07	0.09	0.00	0.61
	Adipocytes	0.08	0.11	0.00	0.92
	Nerve	0.01	0.03	0.00	0.68
	Red blood cells	0.01	0.02	0.00	0.21
	Blood clot	0.00	0.01	0.00	0.10
Colon (Transverse)	Mucosa	0.16	0.11	0.00	0.82
	Submucosa	0.35	0.09	0.03	0.84
	Mucus	0.07	0.07	0.00	0.21
	Muscularis	0.33	0.14	0.00	0.25
	Adipocytes	0.10	0.08	0.00	0.72
Colon (Sigmoid)	Mucosa	0.03	0.06	0.00	0.58
	Submucosa	0.12	0.09	0.01	0.56
	Chronic colitis	0.01	0.03	0.00	0.27
	Muscularis	0.64	0.18	0.01	0.95
	Adipocytes	0.03	0.05	0.00	0.53
	Adventitia	0.16	0.07	0.02	0.75
	Autolysed mucosa	0.01	0.02	0.00	0.26
Esophagus Mucosa	Epithelium	0.23	0.13	0.00	0.61
	Smooth muscle	0.26	0.09	0.00	0.90
	Stroma	0.38	0.11	0.00	0.96
	Inflammation	0.03	0.03	0.00	0.30
	Erosive esophagitis	0.04	0.06	0.00	0.67
	Submucosal gland	0.01	0.03	0.00	0.34
	Congestion	0.05	0.04	0.00	0.30
Mammary Tissue Breast	Duct	0.00	0.01	0.00	0.10
	Stroma	0.38	0.28	0.00	0.98
	Adipocytes	0.58	0.31	0.00	1.00
	Lobule	0.03	0.05	0.00	0.62
	Nerve	0.01	0.01	0.00	0.09
	Gynecomastoid hyperplasia	0.00	0.00	0.00	0.07
Skin (Sun exposed)	Dermis	0.62	0.13	0.00	0.84
	Eccrine gland	0.07	0.02	0.02	0.21
	Hair follicle	0.01	0.02	0.00	0.25
	Papillary dermis	0.02	0.02	0.00	0.21
	Adipocytes	0.15	0.09	0.02	0.72
	Sebaceous gland	0.00	0.01	0.00	0.08
	Squamous epithelium	0.06	0.02	0.00	0.15

	Solar elastosis	0.03	0.05	0.00	0.49
	Vessel	0.02	0.02	0.00	0.26
Skin (Suprapubic)	Dermis	0.65	0.14	0.00	0.88
	Eccrine gland	0.06	0.02	0.02	0.22
	Hair follicle	0.02	0.02	0.00	0.20
	Papillary dermis	0.03	0.02	0.00	0.17
	Adipocytes	0.12	0.09	0.01	0.54
	Sebaceous gland	0.01	0.01	0.00	0.07
	Squamous epithelium	0.06	0.02	0.02	0.17
	Solar elastosis	0.01	0.02	0.00	0.30
	Vessel	0.02	0.01	0.00	0.16
Coronary Artery	Calcification	0.03	0.07	0.00	0.57
	Adipocytes	0.34	0.22	0.00	0.95
Tibial Nerve	Adipocytes	0.29	0.14	0.01	0.97
	Nerve	0.49	0.14	0.00	0.88
Esophagus Muscularis	Epithelium	0.00	0.02	0.00	0.35
	Inflammation	0.00	0.01	0.00	0.13
	Glands	0.00	0.00	0.00	0.08

Supplementary Table 3: Mean, standard deviation, minimum and maximum value of image derived phenotypes. In order to detect and quantify coronary artery, tibial nerve and esophagus muscularis phenotypes, we used the annotations from tibial artery and esophagus mucosa respectively. Despite not having annotations from those specific tissues, it was possible to identify some shared substructures and pathological features.

Tissue	Training set	Validation set	Test set
Adipose Tissue	526	75	59
Aorta	340	43	47
Atrial Appendage	340	42	45
Colon (Transverse)	327	41	44
Colon (Sigmoid)	294	37	39
Coronary Artery	199	21	19
Esophagus Mucosa	443	56	57
Esophagus Muscularis	422	42	46
Gastroesophageal Junction	302	35	35
Heart	351	33	42
Lung	453	62	57
Mammary Tissue Breast	371	43	42
Omentum	422	61	55
Pancreas	264	27	36
Pituitary Gland	217	36	29
Skeletal Muscle	641	78	78
Skin (Sun Exposed)	557	73	72

Skin (Suprapubic)	472	63	66
Stomach	295	29	38
Testis	284	34	42
Thyroid Gland	522	63	65
Tibial Artery	531	67	60
Tibial Nerve	495	65	60

Supplementary Table 4: Number of train, validation and test samples per tissue/model.

Tissue	Median r score	#Genes r > 0.50	#Genes r > 0.75	#Genes regressed	#Significant genes (FDR1%)
Adipose Tissue	0.25	946	2	11,413	3,761
Aorta	0.22	629	9	11,591	2,169
Atrial Appendage	0.40	2,195	58	8,857	5,039
Colon (Transverse)	0.44	4,948	590	12,002	6,982
Colon (Sigmoid)	0.33	2,356	127	11,683	4,178
Coronary Artery	0.19	1,517	136	12,163	834
Esophagus Mucosa	0.48	4,869	278	10,975	8,575
Esophagus Muscularis	0.35	2.598	123	11,014	4,888
Gastroesophageal Junction	0.46	4,866	639	11,217	6,341
Heart	0.65	6,097	1,384	7,538	6,894
Lung	0.35	2,983	224	13,115	6,819
Mammary Tissue Breast	0.39	3,327	129	12,471	6,118
Omentum	0.37	2,067	4	11,582	6,449
Pancreas	0.58	5,042	378	7,370	5,953
Pituitary Gland	0.13	475	3	12,788	705
Skeletal Muscle	0.52	4,516	289	8,305	7,425
Skin (Sun Exposed)	0.20	138	0	11,538	2,565
Skin (Suprapubic)	0.21	187	0	11,514	2,712
Stomach	0.39	3,174	36	11,530	5,093
Testis	0.50	7,473	960	14,994	9,798
Thyroid Gland	0.36	2,817	49	12,970	7,778
Tibial Artery	0.31	1,648	73	11,282	5,196
Tibial Nerve	0.19	298	5	12,614	2,313

Supplementary Table 5: Summary of RNAPath results across tissues in the test set (median correlation, number of genes with correlation coefficient r > 0.5 and r > 0.75, total number of regressed genes and their FDR1% significance).
