# Supplementary material for: Self-supervised learning for characterising histomorphological diversity and spatial RNA expression prediction across 23 human tissue types
Source: Nat Commun. 2024 Jul 13;15:5906. doi: 10.1038/s41467-024-50317-w (PMC11246527; doi:10.1038/s41467-024-50317-w)
Supplement: Supplementary file 3 — Reporting Summary [file 41467_2024_50317_MOESM3_ESM.pdf]

nature portfolio

Corresponding author(s): Dr Craig A Glastonbury

Last updated by author(s): Jun 17, 2024

Reporting Summary

Nature Portfolio wishes to improve the reproducibility of the work that we publish. This form provides structure for consistency and transparency in reporting. For further information on Nature Portfolio policies, see our Editorial Policies and the Editorial Policy Checklist.

Statistics

For all statistical analyses, confirm that the following items are present in the figure legend, table legend, main text, or Methods section.

n/a	Confirmed	
☐	☒	The exact sample size (*n*) for each experimental group/condition, given as a discrete number and unit of measurement
☐	☒	A statement on whether measurements were taken from distinct samples or whether the same sample was measured repeatedly
☐	☒	The statistical test(s) used AND whether they are one- or two-sided<br>*Only common tests should be described solely by name; describe more complex techniques in the Methods section.*
☐	☒	A description of all covariates tested
☐	☒	A description of any assumptions or corrections, such as tests of normality and adjustment for multiple comparisons
☐	☒	A full description of the statistical parameters including central tendency (e.g. means) or other basic estimates (e.g. regression coefficient) AND variation (e.g. standard deviation) or associated estimates of uncertainty (e.g. confidence intervals)
☐	☒	For null hypothesis testing, the test statistic (e.g. F, t, r) with confidence intervals, effect sizes, degrees of freedom and P value noted<br>*Give P values as exact values whenever suitable.*
☒	☐	For Bayesian analysis, information on the choice of priors and Markov chain Monte Carlo settings
☒	☐	For hierarchical and complex designs, identification of the appropriate level for tests and full reporting of outcomes
☐	☒	Estimates of effect sizes (e.g. Cohen's d, Pearson's r), indicating how they were calculated

Our web collection on statistics for biologists contains articles on many of the points above.

Software and code

Policy information about availability of computer code

Data collection	Data are from the publicly available GTEx consortium and were obtained via an approved dbGAP application
Data analysis	All analysis conducted in Python 3.7 with the following packages:<br>h5py==3.8.0<br>imgaug==0.4.0<br>matplotlib==3.7.2<br>numpy==1.21.6<br>opencv_python==4.7.0.72<br>openslide_python==1.1.1<br>pandas==1.2.4<br>Pillow==10.0.0<br>PyYAML==6.0.1<br>scikit_image==0.17.2<br>scikit_learn==1.3.0<br>scipy==1.4.1<br>skimage==0.0<br>statsmodels==0.13.5<br>tensorboardX==2.6.2.2<br>tensorqtl==1.0.7<br>tifffile==2020.2.16<br>torch==1.12.1

```
torchstain==1.3.0
torchvision==0.13.1
tqdm==4.64.0
matplotlib==3.1.1
```

For manuscripts utilizing custom algorithms or software that are central to the research but not yet described in published literature, software must be made available to editors and reviewers. We strongly encourage code deposition in a community repository (e.g. GitHub). See the Nature Portfolio guidelines for submitting code & software for further information.

Data

Policy information about availability of data

All manuscripts must include a data availability statement. This statement should provide the following information, where applicable:

- Accession codes, unique identifiers, or web links for publicly available datasets
- A description of any restrictions on data availability
- For clinical datasets or third party data, please ensure that the statement adheres to our policy

GTex v8 data are accessible via an approved dbGAP application (accession: phs000424.v8.p2). All code, model weights, annotations and summary files are available on our github page

Research involving human participants, their data, or biological material

Policy information about studies with human participants or human data. See also policy information about sex, gender (identity/presentation), and sexual orientation and race, ethnicity and racism.

Reporting on sex and gender	For any male/female specific analyses, we refer to the gametic sex (XX/XY) for females and males respectively
Reporting on race, ethnicity, or other socially relevant groupings	All analyses were on european ancestry individuals to avoid population structure effects in the genetic analyses
Population characteristics	All participants of the GTEx cohort experienced sudden death. 33% are female, 67% are male. Age spans from 20-70 years old. They were not specifically recruited for a disease of interest.
Recruitment	All participants were organ donors and consented to medical research
Ethics oversight	This is an NIH approved study (GTEx) in which deceased donors are not classified as human subjects research under 45 CRF 46

Note that full information on the approval of the study protocol must also be provided in the manuscript.

Field-specific reporting

Please select the one below that is the best fit for your research. If you are not sure, read the appropriate sections before making your selection.

☒ Life sciences ☐ Behavioural & social sciences ☐ Ecological, evolutionary & environmental sciences

For a reference copy of the document with all sections, see nature.com/documents/nr-reporting-summary-flat.pdf

Life sciences study design

All studies must disclose on these points even when the disclosure is negative.

Sample size	n = 13898 was the total number of whole slide images used. We determined this by taking all WSI with a sample size of at least 200 subjects. This was to ensure enough data per tissue type was available for training our models.
Data exclusions	WGS individuals were excluded based on sample quality control, such as mismatched sex or low call rates.
Replication	All machine learning predictions were replicated in a held out dataset (TCGA)
Randomization	Groups were allocated based on disease status. For example, having arterial calcification or not. In statistical analyses we adjusted for common confounders that could vary in these groups, such as age, sex and BMI.
Blinding	Not necessary as we are not testing the effect of a drug.

Reporting for specific materials, systems and methods

We require information from authors about some types of materials, experimental systems and methods used in many studies. Here, indicate whether each material, system or method listed is relevant to your study. If you are not sure if a list item applies to your research, read the appropriate section before selecting a response.

Materials & experimental systems

n/a	Involved in the study
☒	☐ Antibodies
☒	☐ Eukaryotic cell lines
☒	☐ Palaeontology and archaeology
☒	☐ Animals and other organisms
☒	☐ Clinical data
☒	☐ Dual use research of concern
☒	☐ Plants

Methods

n/a	Involved in the study
☒	☐ ChIP-seq
☒	☐ Flow cytometry
☒	☐ MRI-based neuroimaging

Plants

Seed stocks	N/A
Novel plant genotypes	N/A
Authentication	N/A
