# Supplementary material for: Self-supervised learning for characterising histomorphological diversity and spatial RNA expression prediction across 23 human tissue types
Source: Nat Commun. 2024 Jul 13;15:5906. doi: 10.1038/s41467-024-50317-w (PMC11246527; doi:10.1038/s41467-024-50317-w)
Supplement: Supplementary file 2 — Peer Review File [file 41467_2024_50317_MOESM2_ESM.pdf]

nature portfolio

Peer Review File

Self-supervised learning for characterising histomorphological diversity and spatial RNA expression prediction across 23 human tissue types

Open Access This file is licensed under a Creative Commons Attribution 4.0 International License, which permits use, sharing, adaptation, distribution and reproduction in any medium or format, as long as you give appropriate credit to the original author(s) and the source, provide a link to the Creative Commons license, and indicate if changes were made. In the cases where the authors are anonymous, such as is the case for the reports of anonymous peer reviewers, author attribution should be to 'Anonymous Referee' followed by a clear attribution to the source work. The images or other third party material in this file are included in the article's Creative Commons license, unless indicated otherwise in a credit line to the material. If material is not included in the article's Creative Commons license and your intended use is not permitted by statutory regulation or exceeds the permitted use, you will need to obtain permission directly from the copyright holder. To view a copy of this license, visit http://creativecommons.org/licenses/by/4.0/.

REVIEWER COMMENTS

Reviewer #1 (Remarks to the Author):

This study highlights the importance of studying histomorphology in its own right. Using state-of-the-art self-supervised learning, the author trained a general-purpose ViT encoder and a kNN to automatically segment WSIs in GTEx into a set of known tissue substructures. They further quantified the variability of substructures across tissue types and their associations in GWAS and eQTL analysis. Finally, we trained a MIL-based regressor to directly predict patch-based gene expression.

Self-supervised training is quickly becoming a state-of-the-art strategy in AI for digital pathology. The method of choice is reasonable. I have a few comments about this part.

1. Pretrained ImageNet ResNet50 is not a state-of-the-art encoder in histology. Encoders are more frequently trained on WSIs rather than ImageNet pretrained. I, therefore, question the added value of the comparison here.

2. There appears to be a 10-fold cross-validation of kNN. Is the self-supervised trained encoder also part of the 10-fold cross-validation? A robust test should also isolate the encoder training.

3. How are the labelled 0.50% tiles chosen? Is there any significant shift in the rest of the tiles? Are there tiles that don't fit to those known substructures?

4. Given the importance of variations in the study, it is also important to show the variation in the kNN's performance across the 10 folds.

I like the study of the variability of tissue substructures and the connection with GWAS and eQTL.

5. I wonder if there is any technical confounder left after self-supervised training. For example, stain variations, differences in resolution i.e. size per pixel, or different institutions?

I found the MIL prediction interesting, but somewhat underdeveloped. I have some comments on this part.

6. There should be clear model descriptions in the Methods section. Left all of it to code repo is not adequate.

7. Were any feed-forward layers or any transformations being used on ViT representations? What was the MIL architecture used here? Was it a weighted average? How could there be patch-specific Y_ij in the objective function, when only the bulk expression profile is available?

8. How should we interpret the IoU score, in the context of image segmentation ~0.4 is considered poor performance.

9. What is the corresponding number of WSIs and genes in the 80:10:10 split? Is the performance reported on the 10% held-out set? Why not cross-validation? I'm concerned with the robustness of the performance in the 10% held-out set.

Reviewer #2 (Remarks to the Author):

In this manuscript, the authors propose a self-supervised learning approach to generate a

representation of histology slides from the GTEx database. They show that this representation contains meaningful information about the morphological structures and can be used to classify tissues in a semi-supervised manner. Based on this, they evaluate the variability among donors and evidence associations between tissue substructure proportions and age, sex and gene expression. Finally, they leverage their representation to train a multiple-instance learning model to predict bulk gene expression from histology images.
While the paper contains several interesting analyses, I have a few concerns.

1. The main concern relates to the absence of external validation. In particular, the self-supervised learning algorithm is trained on the same data that is used for all downstream analyses. This raises the question of the applicability of the methods (both the tissue classification and the gene expression prediction) to external datasets generated with different protocols and scanners. In my opinion, this should be addressedd before publication.

2. The validation of the gene expression prediction at the level of individual tiles is purely quantitative (Figure 5). The presented examples look convincing, but since the last part, associating local gene expressions with tissue substructure - which is in my opinion an important contribution of this work - relies on this, a quantitative validation appears necessary.

3. The main machine learning methods presented here are not new. Several works applying self-supervised learning to histology data have already been published. In particular, the choice of an ImageNet pretrained model as the baseline to compare against is debatable. The prediction of gene expression from histology slides has also been the subject of several previous articles. While the RNAPath approach shows better performance than HE2RNA (the only other method presented here), since it just consists of a set of linear models, it cannot really be described as "novel".
As such, this is not a blocking point because the downstream analyses are still interesting, but some statements should be tempered, in particular:
- L. 51-52 "with some preliminary applications focused on histopathology". As mentioned earlier, there have already been several works focusing on self-supervised learning applied to histology data, including for instance the following references:
* Saillard, C., Dehaene, O., Marchand, T., Moindrot, O., Kamoun, A., Schmauch, B., & Jegou, S. (2021). Self supervised learning improves dMMR/MSI detection from histology slides across multiple cancers. arXiv preprint arXiv:2109.05819.
* Ciga, O., Xu, T., & Martel, A. L. (2022). Self supervised contrastive learning for digital histopathology. Machine Learning with Applications, 7, 100198.
* Srinidhi, C. L., Kim, S. W., Chen, F. D., & Martel, A. L. (2022). Self-supervised driven consistency training for annotation efficient histopathology image analysis. Medical Image Analysis, 75, 102256.
* Saldanha, O. L., Loeffler, C. M., Niehues, J. M., van Treeck, M., Seraphin, T. P., Hewitt, K. J., ... & Kather, J. N. (2023). Self-supervised attention-based deep learning for pan-cancer mutation prediction from histopathology. NPJ Precision Oncology, 7(1), 35.
* Chen, R. J., Chen, C., Li, Y., Chen, T. Y., Trister, A. D., Krishnan, R. G., & Mahmood, F. (2022). Scaling vision transformers to gigapixel images via hierarchical self-supervised learning. In Proceedings of the IEEE/CVF Conference on Computer Vision and Pattern Recognition (pp. 16144-16155).
- L. 87-88 "ImageNet pre-trained models which are the standard in the field". Same comment, as the field is evolving rapidly, I do not think it is right anymore to say it is the standard.
- L. 669 "Our novel method, RNAPath": I would remove "novel".

Minor points

4. The methodology for the weakly supervised segmentation of histology images could be clarified a little bit. In the Methods section (L. 595) it is said that "a small number of tiles from 5-10 WSI per tissue" were annotated. The authors should specify how the tiles were sampled (were they randomly chosen or selected in a way?). Even if the information is present in the supplementary material, the

"small number" could be made explicit here. It is also not clear who actually performed the annotation, as the text only says (L.147) that the annotations "were validated by a clinical histopathologist".

5. Do all annotated patches belong to only one class? If so, what if a patch lies at the border between two histological structures?

6. L.152-153, the methodology for the 10-fold cross-validation should also be clarified. In particular, did the authors ensure that patches from the same slides were not present both in the training and test set? If yes, this should be mentioned explicitly. Otherwise, the reported accuracy is probably optimistic.

7. L.160-165, "Tiles inferred as belonging to the calcification class had high sensitivity in recovering ground-truth pathologist labels, with AUC = 0.94 (Supplementary Figure 4), indicating that the vast majority of true positive cases were correctly identified by the kNN segmentation model": AUC is not the right metric to evaluate the model's sensitivity and the number of true positives.

8. In section "Sex and age-specific variability in tissue substructure and pathological features" (L. 234), the statistical test used should be mentioned, as well as whether any correction was applied for multiple hypothesis testing.

9. P-values, confidence intervals or standard deviations of cross-validation results should be reported, for instance
- L.154-155 and supp. table 1
- When describing RNAPath results (in the text and supp. table 3). It would also be interesting to report how many genes are regressed with a significant p-value.

10. When reporting the IoU of well predicted genes between different tissues, again the significance of the result should be assessed more rigorously (L. 387-388 and supp. fig. 12). In particular, how many genes were predicted in both tissue types, and what is the p-value of the result.

11. L. 398-399, "Surprisingly, we see high concordance between our spatially resolved RNA expression predictions and that of matched antibody staining": I am not sure the word "surprisingly" is appropriate here, as similar results were shown in
* Fu, Y. et al. Pan-cancer computational histopathology reveals mutations, tumor composition and prognosis. Nat Cancer 1, 800-810 (2020)
* Schmauch, B. et al. A deep learning model to predict RNA-Seq expression of tumours from whole slide images. Nat. Commun. 11, 3877 (2020).

Reviewer #3 (Remarks to the Author):

This article addresses an interesting and timely concept. The authors use self-supervised learning (SSL) to train a foundation model for tissue image analysis. The approach is interesting and the paper could be a good fit for this journal, but there are a number of fundamental issues which need to be fixed.

- The abstract is very poorly written. Even for a researcher in this field, the abstract is too convolved and poorly understandale. This is exacerbated by the fact that the authors do not define abbreviations such as GTEx or eQTL. Also, the abstract is too long and poorly structured. The abstract needs to be completely rewritten to provide an adequate background, information on the methods and technical

novelty, then to provide quantitative (!) data and short interpretation of the findings.

- the results section is really long and convoluted and poorly structured.

- The authors use DINO but this is not state of the art anymore. DINO-v2 has been out for quite some time and is far superior. Other studies, such as the recent Nature paper on a retinal photograph foundation model by the Keane lab have systematically evaluated multiple SSL methods. This needs to be addressed by additional experiments.

- The statement that "previous ImageNet pre-trained models" are the standard in the field is simply untrue. For more than a year, publicly available models which are pre-trained with SSL are available, e.g. RetCCL and Ctranspath. Dozens of studies have been published using these SSL-trained models. Recent papers on new SSL-trained models (please check recent papers by the Mahmood lab at Harvard, by Owkin and by Thomas Fuchs) have compared their performance to Ctranspath. The authors only compare to imagenet-trained models and thereby choose an unrealistically low-quality benchmark. This needs to be addressed by additional experiments.

- The figures have a suboptimal visual quality and there are some typos in the figures, e.g. "Features extraction" instead of "Feature extraction". Also, abbreviations in the figures need to be explained in the legend. Finally, all histopathology images in the figures require scale bars as required by the journal's author guidelines.

- Figure 2,3,4,5,6,7 need panel labels A, B, C and scale bars and a legend for the color code and color scale bars with unit labels for the heat maps which are shown

- UMAP plots should not be quantitatively interpreted. UMAP is sensitive to hyperparameter choices.

- the authors cite only 42 references which is not a lot for this massive article. The authors omit a few relevant references on the preidciton of gene expression from pathology slides (e.g. https://pubmed.ncbi.nlm.nih.gov/35143898/ and https://www.nature.com/articles/s41467-020-17678-4 and https://www.nature.com/articles/s43018-020-0087-6 and https://www.thelancet.com/journals/ebiom/article/PIIS2352-3964(21)00181-X/fulltext and several others)

Reviewer #1 (Remarks to the Author):

This study highlights the importance of studying histomorphology in its own right. Using state-of-the-art self-supervised learning, the author trained a general-purpose ViT encoder and a kNN to automatically segment WSIs in GTEx into a set of known tissue substructures. They further quantified the variability of substructures across tissue types and their associations in GWAS and eQTL analysis. Finally, we trained a MIL-based regressor to directly predict patch-based gene expression.

Self-supervised training is quickly becoming a state-of-the-art strategy in AI for digital pathology. The method of choice is reasonable. I have a few comments about this part.

We thank the reviewer for taking the time to read our manuscript and providing extremely helpful feedback. We are pleased that the reviewer shares our opinion that histomorphology and its variation in a population is an important topic in its own right and that our approach, including the use of self-supervised learning, is reasonable. We have addressed all questions in detail line-by-line below. To summarise the main changes briefly:

1. We have performed an additional experiment demonstrating our DINO learnt representations not only outperform ImageNet-ResNet50 but also lead to a 43% higher median silhouette score as compared to CTransPath, a multi-scale Swin transformer model pre-trained on 15M histology images (see updated **Figure 2** and **Supplementary Figure 3** as well as results in the main text).
2. We have clarified the reviewers' questions on our use of cross-validation by including performance across cross validation folds
3. We have improved the description of how annotations were obtained and used
4. We have performed an additional experiment to demonstrate no technical confounding remains due to the institute/centre the sample was collected in both at the level of derived substructures and underlying DINO embeddings.
5. We have improved the description of our models in our methods section and clarified the use of IoU computation to compare gene lists.

We again thank the reviewer for all their helpful suggestions and hope we have been able to systematically address all of their concerns with new experiments, additional data and clearer text.

1. Pretrained ImageNet ResNet50 is not a state-of-the-art encoder in histology. Encoders are more frequently trained on WSIs rather than ImageNet pretrained. I, therefore, question the added value of the comparison here.

We thank the reviewer for their comment. However, the primary reason we include a comparison to ImageNet representations is because whilst we agree with the reviewer they are not the state of the art anymore, they are still widely used in practice (Huang *et al.,* 2023; Azizi *et al.,* 2023; Chen *et al.,* 2022). However, we do recognise that this field moves incredibly

quickly and large pretrained histopathology models have now been released, such as CTransPath.

To compare our approach to a more state of the art baseline, we have included as the reviewer (as well as Reviewer 2, Q3, Reviewer 3, Q4) an additional experiment that benchmarks our DINO learnt histology embeddings from GTEx, versus both ImageNet pretrained models and CTransPath, a Swin-transformer pre-trained on 15M histology images. We have modified **Figure 2 and Supplementary Figure's 2 & 3** to include this comparison and reproduce them below for the reviewers convenience:

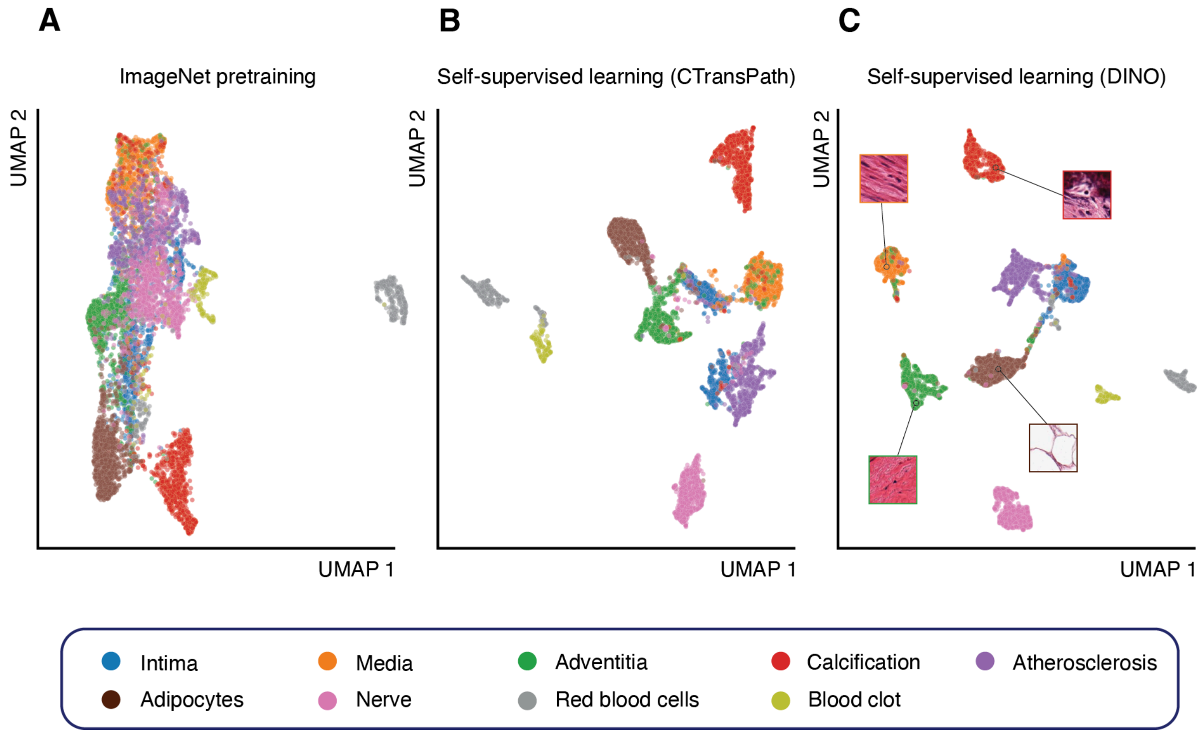

Updated Figure 2. UMAP embeddings of tibial artery tile features from self-supervised ViT-S (trained using DINO) versus CTransPath (pretrained on 15M histology images) and ResNet50 with pretrained weights from ImageNet. Tiles have been manually labeled with tissue substructures/pathologies to interpret clusters. DINO embeddings show both better qualitative UMAP clustering but also higher quantitative silhouette scores, ~ 43% higher than CTransPath.

As you can see, our GTEx histology representations learnt using DINO not only outperform ImageNet pretrained embeddings but also CTransPath, with a 43% higher median silhouette score across a range of k = [3,20]: ImageNet embeddings = 0.097 CTransPath embeddings = 0.137, DINO Embeddings = 0.196 **(Tibial Artery);** ImageNet embeddings = 0.103, CTransPath embeddings = 0.138, DINO embeddings = 0.227 **(Esophagus Mucosa)**.

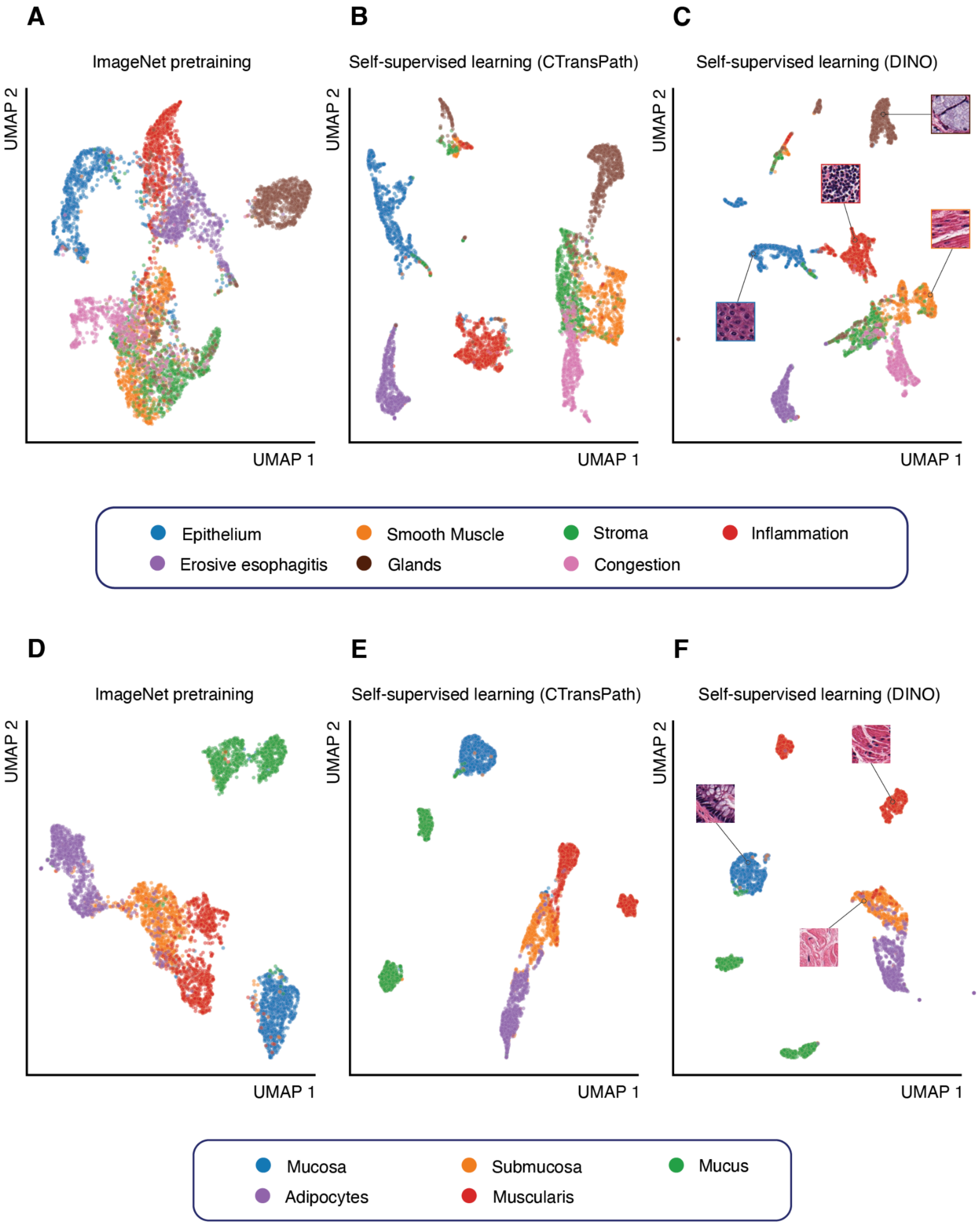

Updated Supplementary Figure 2: UMAP embeddings of esophagus mucosa tile features from ResNet50-ImageNet (A), CTransPath (B) and DINO (C) and Colon: ResNe50-ImageNet (D), CtransPath (E), DINO (F). DINO embeddings consistently outperform the other models.

We also see that if we assume the true number of clusters reflects the number of annotations present, **k=9**, our DINO representations outperform both methods again, by a significant margin (**Updated Supplementary Figure 4**). We believe this additional experiment furthers our evidence that our self-supervised representations richly capture the underlying histomorphology and are competitive with state of the art approaches for our use-cases.

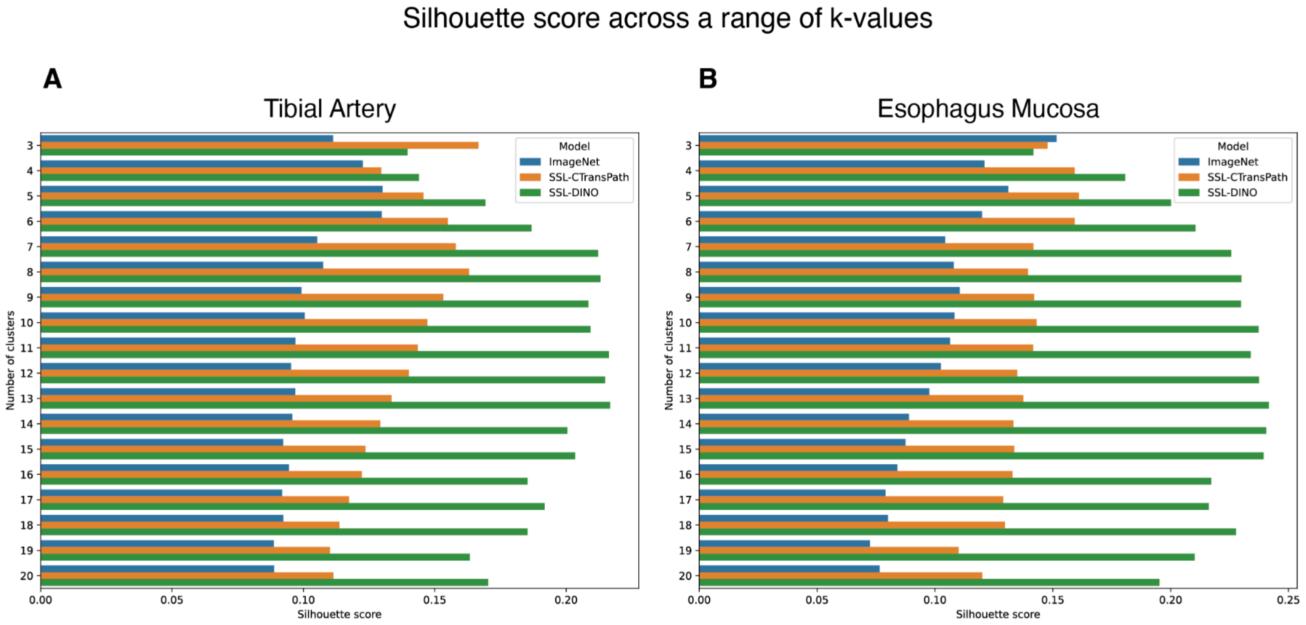

Updated Supplementary Figure 4: Comparison of silhouette scores across several possible cluster values k=[3,20] for tibial artery (**A**) and esophagus mucosa (**B**). Models compared include ImageNet pretraining and self-supervised (SSL) learning (CTransPath, DINO). DINO consistently outperforms the other models.

References:

1. Huang, Z., Bianchi, F., Yuksekgonul, M., Montine, T.J. and Zou, J., 2023. A visual–language foundation model for pathology image analysis using medical twitter. Nature medicine, 29(9), pp.2307-2316.
2. Azizi, S., Culp, L., Freyberg, J., Mustafa, B., Baur, S., Kornblith, S., Chen, T., Tomasev, N., Mitrović, J., Strachan, P. and Mahdavi, S.S., 2023. Robust and data-efficient generalization of self-supervised machine learning for diagnostic imaging. Nature Biomedical Engineering, pp.1-24.
3. Chen, C., Lu, M.Y., Williamson, D.F., Chen, T.Y., Schaumberg, A.J. and Mahmood, F., 2022. Fast and scalable search of whole-slide images via self-supervised deep learning. Nature Biomedical Engineering, 6(12), pp.1420-1434.

2. There appears to be a 10-fold cross-validation of kNN. Is the self-supervised trained encoder also part of the 10-fold cross-validation? A robust test should also isolate the encoder training.

We thank the reviewer for their question. If we understand correctly, the reviewer is asking whether the encoder training was part of the 10-fold cross validation. The answer is no. The DINO/ViT encoder is not coupled to the kNN, in the sense that the training of the encoder is isolated from the evaluation of the kNN model, where the input to the kNN are the tile features (DINO representations) and the labels are the tissue substructures. Therefore, what we have currently reported in the manuscript does indeed represent a 10-fold cross-validation of the kNN only, with the encoder remaining fixed. Please see lines **L164-168** that now clarify this.

3. How are the labelled 0.50% tiles chosen? Is there any significant shift in the rest of the tiles? Are there tiles that don't fit to those known substructures?

We thank the reviewer for their comment and completely agree that this section could have been clearer and more explicit. We have added more information to the methods section regarding how annotated tiles were selected. We briefly summarize here the new lines **L684-692** for the reviewers convenience:

Tiles were selected by author C.A.G. and all of them were confirmed by a clinical histopathologist (author: A.P.L) using the following criteria:

1. WSI were randomly selected to extract tiles to include major tissue substructures. For example in arterial tissue: Intima, Media and Adventitia layers. These can be thought of as the canonical structures present across the majority of tissue specimens.
2. Pathology notes for tissue specimens were inspected to additionally include donors where incidental structures were known to be present: e.g. presence of hair follicles, or eccrine glands in skin, which are not always present given tissue sampling variability.
3. Pathology notes for specimens were also examined to include incidental pathology findings. For example, atherosclerosis or calcification in arterial samples.

These inclusion criteria enabled us to have broad coverage of tissue substructures that are common across donors for a given tissue, whilst ensuring we can segment incidental pathology findings. These classes are not exhaustive but missing classes can be handled by the model. For example in skin, there are specimens that have solar elastosis. Whilst we annotate this mild pathology (changes due to sun exposure), it's still technically dermis. If we hadn't annotated solar elastosis, these tiles would still be successfully segmented as belonging to dermis. If users have additional and specific substructure or pathologies they are interested in, it is quite easy to extend our method to use additional annotations and we do recommend annotations cover the widest range of substructures known to a particular dataset.

Furthermore, the reviewer asks about significant shifts in the rest of the tiles. Given our kNN uses a majority vote and the fact within tissue substructure tiles are far more similar than tiles between two distinct substructures (i.e. epidermis tiles look more similar to each other even if

varying as compared to epidermis vs dermis), this doesn't represent a problem in practice, as a tile that looks slightly different to those annotated, will still have a smaller distance between its correct annotation, then to a different class.

Finally, the gene expression prediction and computation of moran's I that we present in the *RNAPath* section of the manuscript does not depend on any annotated substructures. Therefore, if genes are specific to substructures or pathologies, and are regressed accurately, this presents an opportunity for *RNAPath* to identify novel substructures via its localised gene expression prediction without any annotations necessary.

4. Given the importance of variations in the study, it is also important to show the variation in the kNN's performance across the 10 folds.

We thank the reviewer for raising this point. We have modified the supplementary table for the tile classification evaluation by including the standard deviation of the accuracy across folds (Updated Supplementary Table 1).

As the reviewer can see, the variability across folds is low, as reflected in the vast majority of standard deviations reported for each tissue substructure and pathology below. Slightly higher variability (always < 10%) is present for classes that are poorly represented and complex to segment, such as autolysed mucosa. However, with an accuracy of 93% ± 0.035 we are happy that our segmentations are accurate and lead to sensible downstream inferences.

Tissue	Phenotype	Annotated Tiles	Accuracy
Artery (Tibial)	Tunica intima	1,038	0.925 ± 0.023
	Tunica media	5,365	0.920 ± 0.024
	Tunica adventitia	3,172	0.900 ± 0.038
	Calcification	1,058	0.939 ± 0.014
	Atherosclerosis	1,780	0.943 ± 0.019
	Adipocytes	5,297	0.964 ± 0.020
	Nerve	1,376	0.982 ± 0.015
	Red blood cells	465	0.951 ± 0.032
	Blood clot	228	0.908 ± 0.060
Colon (Transverse)	Mucosa	2,680	0.979 ± 0.018
	Submucosa	4,030	0.960 ± 0.024
	Mucus	1,215	0.987 ± 0.012
	Muscularis	2,752	0.941 ± 0.019
	Adipocytes	953	0.914 ± 0.021
Colon (Sigmoid)	Mucosa	2,692	0.945 ± 0.021
	Submucosa	2,628	0.702 ± 0.036
	Chronic colitis	541	0.919 ± 0.029
	Muscularis	5,545	0.972 ± 0.009
	Adipocytes	823	0.855 ± 0.046
	Adventitia	1,030	0.834 ± 0.031
	Autolysed mucosa	196	0.816 ± 0.092

Esophagus Mucosa	Epithelium	1,530	0.919 ± 0.038
	Smooth muscle	2,963	0.873 ± 0.045
	Stroma	4,328	0.851 ± 0.038
	Inflammation	884	0.937 ± 0.027
	Erosive esophagitis	747	0.973 ± 0.026
	Submucosal gland	1,822	0.923 ± 0.027
	Congestion	630	0.886 ± 0.027
Mammary Tissue Breast	Duct	471	0.495 ± 0.078
	Stroma	11,399	0.963 ± 0.021
	Adipocytes	12,820	0.976 ± 0.011
	Lobule	803	0.940 ± 0.019
	Nerve	328	0.829 ± 0.039
	Gynecomastoid hyperplasia	262	0.771 ± 0.097
Skin	Dermis	1,464	0.973 ± 0.018
	Eccrine gland	763	0.885 ± 0.038
	Hair follicle	960	0.850 ± 0.034
	Papillary dermis	218	0.288 ± 0.066
	Adipocytes	2,727	0.903 ± 0.028
	Sebaceous gland	653	0.847 ± 0.044
	Squamous epithelium	470	0.819 ± 0.067
	Solar elastosis	178	0.820 ± 0.084
	Vessel	630	0.835 ± 0.049

Updated Supplementary Table 1: Accuracy of tile classification across tissues with variability reported across 10-fold cross validation.

I like the study of the variability of tissue substructures and the connection with GWAS and eQTL.

5. I wonder if there is any technical confounder left after self-supervised training. For example, stain variations, differences in resolution i.e. size per pixel, or different institutions?

We thank the reviewer for this interesting question. In regards to the specific question around stain variation and resolution differences, we took two approaches to handle this. Whilst training our models, we perform stain normalisation (Macenko *et al.,* 2009) and colour augmentation to alleviate differences in H&E intensity across tissues and donors. With respect to resolution differences, all GTEx slides are acquired at 20x objective magnification and have a micron per pixel (MPP) value of 0.495 so there is no variation in resolution across tissues or donors. As GTEx is a large multi-institutional study we have now included an assessment of whether our

substructure/pathology phenotypes or the underlying self-supervised representations cluster according to institute/hospitals. As you can see from the UMAP below, neither the derived substructure proportions nor the DINO tile level representations cluster by the institute/centre the sample was enrolled and processed in. We have now included this additional experiment as **Supplementary Figure 3** and added to the result (see new lines: **L136-137**).

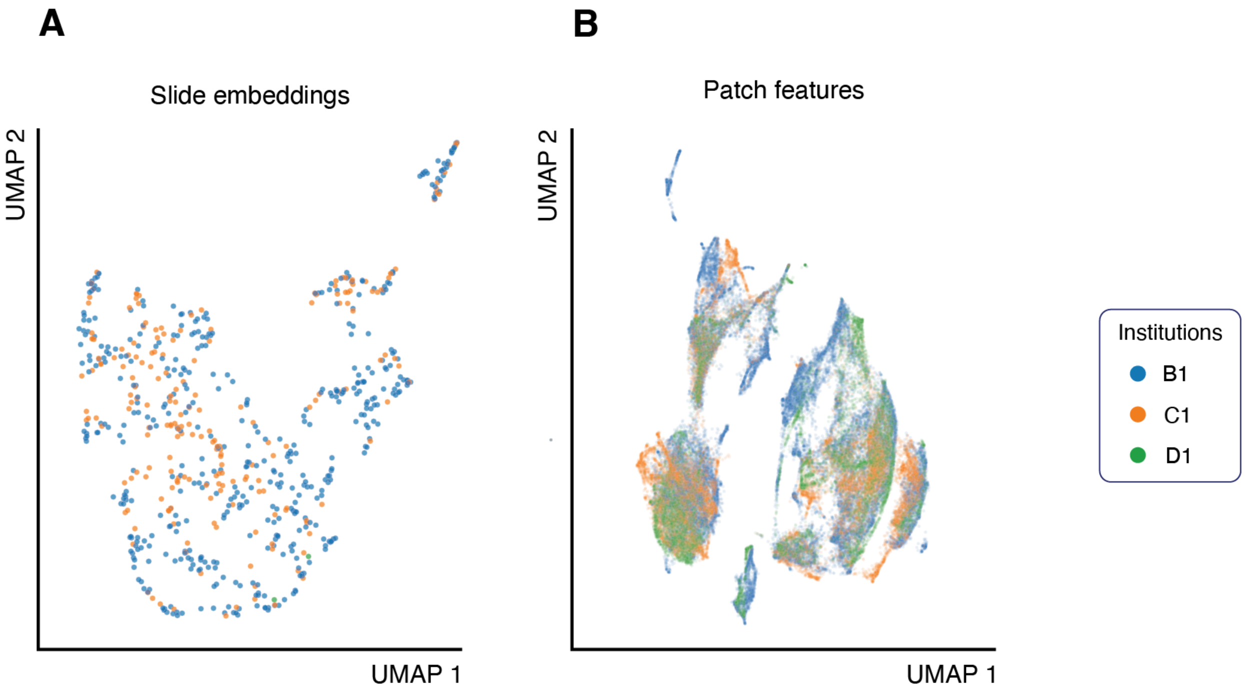

Supplementary Figure 3: UMAP demonstrating lack of residual confounding by institution/centre. (A) Image derived phenotypes for 687 tibial artery samples coloured by different collection centres/institutions. (B) Tile representations from a total of 33 slides, coloured by the institution where the sample was collected. Both IDPs used in downstream analysis and tile representations do not cluster by centre/institution.

References:

1. Macenko, M. et al. A method for normalizing histology slides for quantitative analysis. in 2009 IEEE International Symposium on Biomedical Imaging: From Nano to Macro 1107–1110 (2009).

I found the MIL prediction interesting, but somewhat underdeveloped. I have some comments on this part.

6. There should be clear model descriptions in the Methods section. Left all of it to code repo is not adequate.

We thank the reviewer for their suggestion. We have improved the model description in the methods section, clearly stating its mathematical basis.

7. Were any feed-forward layers or any transformations being used on ViT representations? What was the MIL architecture used here? Was it a weighted average? How could there be patch-specific Y_ij in the objective function, when only the bulk expression profile is available?

We thank the reviewer for their question. *RNAPath* learns a linear mapping between the ViT learnt representations and the regressed gene expression outcome without any intermediate hidden layers. This enables clear, identifiable and interpretable (linear) tile-level gene expression predictions. Whilst the addition of non-linear activation functions and additional fully connected layers could lead to increased performance, it would hinder the direct and simple interpretation of our tile-level predictions. The reviewer is correct that our MIL architecture is a simple weighted average. The Y_{ij} in the objective function does not refer to tile-specific expression, but refers to the ground-truth bulk expression level of gene *j*, in sample *i*. $\hat{Y}_{ij}$ is the aggregated tile-level expression prediction, forming a single prediction of the bulk-level expression per sample, used in the objective function. We have now clarified this in the methods section: "*RNAPath: Multiple instance learning for gene expression regression*": **- L782-L841**.

8**. How should we interpret the IoU score, in the context of image segmentation ~0.4 is considered poor performance.**

We thank the reviewer for their comment. It should be noted that the reference to IoU in our manuscript **should not be compared to image segmentation performance**, but as a metric to quantify gene expression sharing across different tissues. **For imaging, as the reviewer points out, an IoU of 1 would represent perfect segmentation/overlap. For our use case here, we would not expect perfect overlap of lists of genes predicted from two different tissues as that is not biologically plausible.** The hypothesis we are testing here is that well predicted genes across tissues should have a higher degree of overlap/sharing for tissues of similar developmental origin or tissues that contain the same canonical substructures as compared to distinct tissues. For example, we might expect that two tissue sections from the gastrointestinal tract will share more tissue substructure similarity (and hence have more similar gene expression) than brain versus adipose tissue, for example. This is indeed what we see. We see that our RNAPath predictions recapitulate known tissue relationships. For example, transverse colon and sigmoid colon share a significant number of well regressed genes (IoU = 0.33, number of genes shared = 1,760) and are structurally and anatomically similar as compared to pituitary gland and omentum (IoU = 0.01, number of shared genes = 11, P-value = 0.71), which are not. In summary, we do not expect to see high IoUs, but we do expect that the relative ranking of such IoUs should recapitulate some known tissue relationships, with more similar tissues having higher IoUs than more distantly related tissues. We edited the manuscript to make this much more clear (see new lines: **L428-432**)

9. What is the corresponding number of WSIs and genes in the 80:10:10 split? Is the performance reported on the 10% held-out set? Why not cross-validation? I'm concerned with the robustness of the performance in the 10% held-out set.

We thank the reviewer for this question. We confirm that reported performance **(Supplementary Table 3**) refers to the 10% held-out set. We did not perform cross-validation as this would have involved training 230 (10 x 23) models, which is not practical computationally.
However, to show how the prediction of gene expression is robust across different train/test split sizes and that our 10% reported estimates are reasonable estimates, we performed cross-validation on two tissues that represent the extremes: The lowest and the highest sample size respectively: coronary artery (*N* = 239) and skeletal muscle (*N* = 797). For coronary artery representing the worst case scenario with smallest test set size, we report a mean median r score of 0.181 ± 0.028, with the original 10% test set r-score reported in **Supplementary Table 3** falling comfortably within these confidence intervals. For skeletal muscle, that is the tissue with larger sample size, the median r score is 0.484 ± 0.021, having a slightly smaller standard deviation.

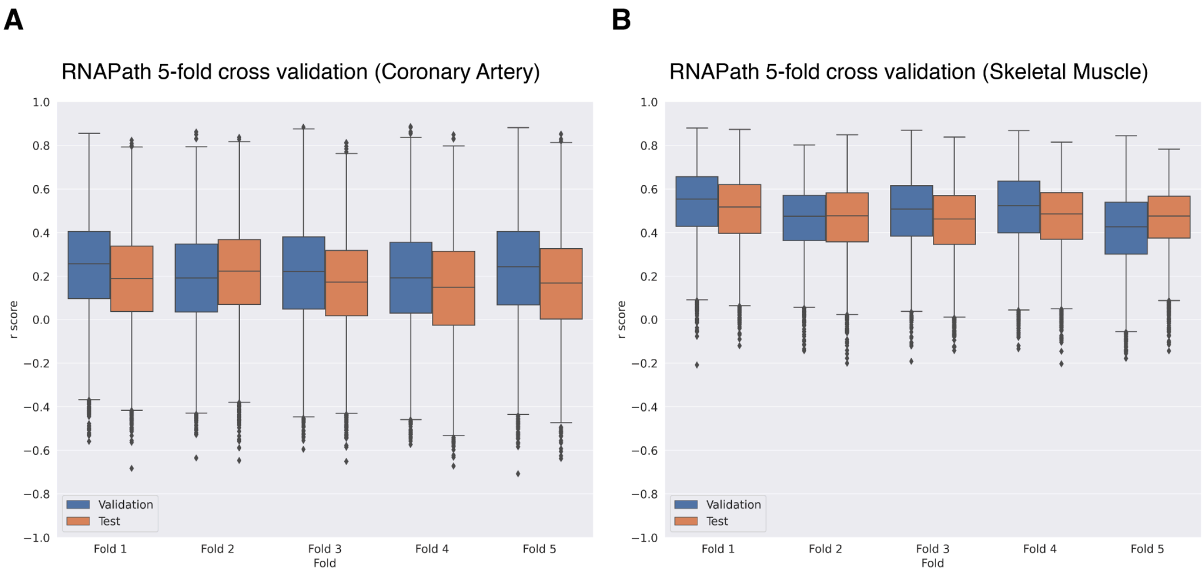

Updated Supplementary Figure 13: RNAPath 5-fold cross validation between the smallest sample size tissue (Coronary Artery) (A) and the largest sample size tissue (Skeletal Muscle) (B).

We have also added a table (**Supplementary Table 4**) to the manuscript, reporting the number of training, validation and test samples used per tissue. We have added a small section in the results detailing this: **L417-L420.**

In conclusion the 5-fold cross validation variability difference between the smallest sample size tissue (n=239) and largest tissue (n=797) was negligible, with estimates from our 10% test set falling within the cross validation range. We hope this reassures the reviewer of our 10% test set estimates.

Reviewer #2 (Remarks to the Author):

In this manuscript, the authors propose a self-supervised learning approach to generate a representation of histology slides from the GTEx database. They show that this representation contains meaningful information about the morphological structures and can be used to classify tissues in a semi-supervised manner. Based on this, they evaluate the variability among donors and evidence associations between tissue substructure proportions and age, sex and gene expression. Finally, they leverage their representation to train a multiple-instance learning model to predict bulk gene expression from histology images.
While the paper contains several interesting analyses, I have a few concerns.

We thank the reviewer for their detailed feedback and suggestions. We believe that by addressing your concerns, we have significantly improved the quality of our manuscript. We have included a detailed line by line response to all your questions below, but also summarise here for brevity:

1. **We have conducted a new experiment that validates our approach on an independent external dataset (n=986, TCGA-BRCA).** We demonstrate our representations generalize to oncology, being able to separate carcinoma from benign tissue as well as incidental normal tissue substructures. We quantify the proportion of carcinoma present in each donor tissue section and demonstrate its impact on survival time. Finally, we validate RNAPath can predict gene expression in normal substructures (i.e. *PLIN1* in adipose tissue, much like GTEx) but also show we can find enriched genes spatially restricted to areas of carcinoma. We believe this should give significant reassurance of the broad utility our approach has and its generalization.
2. **We have conducted an additional experiment that benchmarks our representations against a transformer based method trained on 15M histopathology images (CTransPath).** We have updated **Figure 2** to reflect this, which demonstrates that our method outperforms (43% higher silhouette score) CTransPath and ImageNet, in terms of our representations identifying specific tissue substructures and pathologies.
3. We have reworked the text and references of our manuscript, incorporating all changes suggested by the reviewer.
4. We have clarified how annotated tiles were obtained, how they belong to a single class, and have detailed the protocol we used.
5. We have implemented all minor points raised by the reviewer around statistical significance of reported results, cross validation variability and have also reported model sensitivity and TP/FP metrics.

We believe the manuscript is now significantly better having addressed all reviewers comments and have included line by line answers below.

1. The main concern relates to the absence of external validation. In particular, the self-supervised learning algorithm is trained on the same data that is used for all downstream analyses. This raises the question of the applicability of the methods (both the tissue classification and the gene expression prediction) to external datasets

generated with different protocols and scanners. In my opinion, this should be addressed before publication.

We thank the reviewer for their comment and understand their concerns relating to external validation. First, we would like to make clear that GTEx is a multi-institutional study that has collected data on >9000 specimens with histology and RNA-seq and therefore have been processed at different sites, by different technicians, different hospitals and storage conditions. Therefore GTEx does not represent a homogeneous dataset in which all samples were uniformly processed and scanned on a single machine, and therefore our model should handle a range of different conditions. We would also like to make clear that all reported predictions and results are on our held-out test set. Having validated our model across 23 distinct human tissues, with many different control experiments, for example:

1. IHC confirmation of gene expression prediction
2. RNAPath predictions recapitulating well known substructure specific control gene specificity (e.g. *PLIN1* in adipocytes, *DCD* in eccrine glands, etc)
3. Validation of substructure segmentation by a trained histopathologist (A.P.L)
4. Use of held-out test sets for all downstream predictions

However, we appreciate the reviewers' concerns and therefore to further validate our approach we have set out to test our method on an independent dataset that is significantly different to GTEx. To do this, we have processed 986 breast carcinoma cases from TCGA (TCGA-BRCA). This dataset is significantly different from GTEx in two ways:

1. Oncology vs healthy tissue with incidental pathology
2. The TCGA-BRCA WSI are acquired using different scanners and with different objective lens magnification (40x vs 20x)

A histopathologist (A.P.L) annotated tissue substructures specific to breast or breast cancer from 15 TCGA-BRCA WSI spanning the following categories: carcinoma, stroma, lymph node, dermis, adipocytes, epidermis, duct ectasia, vessel and nerve. We extracted 256x256 level 0 tiles (40x) to match the resolution of GTEx 128x128 tiles (20x). Annotated tile representations obtained from our frozen ViT model (no fine-tuning on TCGA), form distinct clusters, with clear separation between carcinoma tissue and benign tissue but also incidental tissue substructures (**Supplementary Figure 19A**). This demonstrates that our learnt representations generalize well out of their training domain.

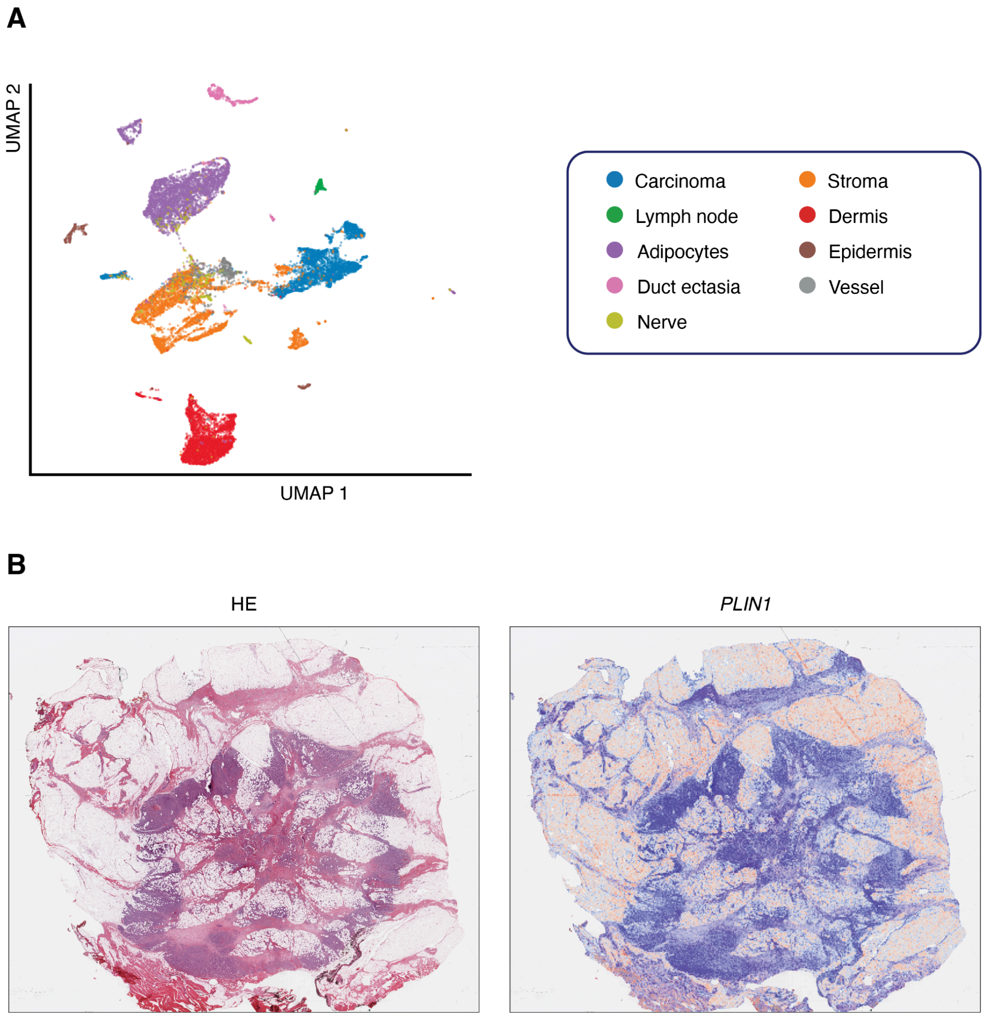

New Supplementary figure 19: (A) UMAP embeddings of BRCA tile features from the DINO model trained on GTEx. (B) The heatmap of *PLIN1*, a marker of adipocytes, from the RNAPath model trained on GTEx mammary tissue samples and applied to a TCGA BRCA histology, clearly shows high expression in adipose tissue.

Using our kNN approach to segment WSI, we estimated the proportion of carcinoma present in 986 samples (**Figure 7A**). Carcinoma proportion varies dramatically in TCGA-BRCA (0.392 ± 0.212) and likely reflects the differential severity of disease at diagnosis amongst patients. To test this, we performed survival analysis, comparing the bottom 25% and top 75% percentiles of carcinoma proportion. A clear negative relationship between carcinoma proportion and survival time is observed (P-value = 0.017) (**Figure 7B**).

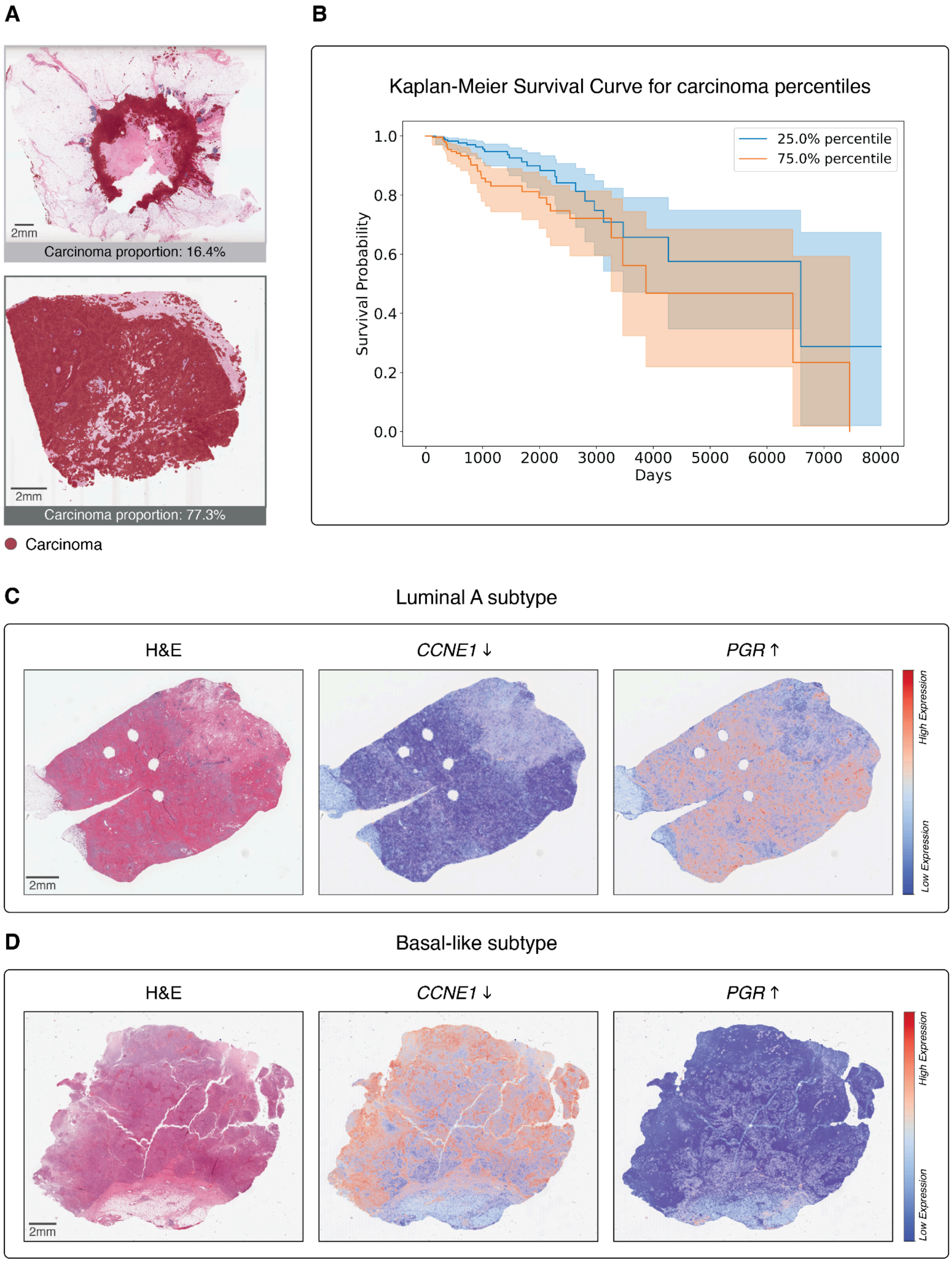

New Figure 7: Segmentation and quantification of carcinoma (**A**). The Kaplan-Meier survival curve identifies a relation between size of carcinoma at diagnosis and survival probability (**B**). Our RNAPath model predictions on the PAM50 signature, used to stratify breast cancer at molecular level, correlates with the ground truth, with luminal A samples (**C**) having low

expression of *CCNE1* and high expression of *PGR* and basal-like samples (**D**) having high expression of *CCNE1* and low expression of *PGR*, as represented in the heatmaps.

To validate *RNAPath* gene expression predictions we demonstrate the ability of *RNAPath* to predict well known substructure marker genes in TCGA-BRCA tissue samples. To do this, we fix our representations and *RNAPath* weights and predict the expression of a canonical marker gene present in adipose, *PLIN1*. As you can see, we recapitulate the well known spatial specificity of *PLIN1* in TCGA samples (**Supplementary Figure 19B**).

As *RNAPath* was never trained on oncology data, to find genes specifically spatially restricted and expressed in areas of breast carcinoma requires fine-tuning. We therefore fixed our representations, but fine-tuned *RNAPath's* linear layer using TCGA-BRCA concurrently measured RNA-sequencing data. We split samples into train (785), validation (100) and test (96) sets by case id, so that samples from the same patients were in the same split (for 5 samples there was no associated gene expression data). Moreover, we selected the 10,000 genes with highest index of dispersion and used their $\log 2(x+1)$ RPKM as the supervisory signal. *RNAPath* was able to regress 351 genes with an r score > 0.5. Cyclin E1 (*CCNE1)* and Progesterone Receptor (*PGR also known as PR),* represent two well known breast cancer genes present in the PAM50 signatures that are used to delineate the different molecular subtypes of breast cancer[1]. We see that *RNAPath* correctly predicts that Luminal-A subtypes are *CCNE1*- *PGR*+, whilst Basal subtypes are *CCNE1*+ *PGR*-, despite having access to no subtype information (**Figure 7D**).

Finally, to find subsets of genes that are specifically restricted to areas of carcinoma, we utilized our substructure specific enrichment score (SSES). We see that 348 genes had SSES scores > 1, for carcinoma, *MUC2* (SSES = 1.29), a well known breast cancer associated gene[2], is predicted to be specifically spatially restricted to areas of carcinoma.

Overall, we see strong generalization of our approach. We believe this forms a robust external validation of our results and demonstrates our findings have broad utility in digital pathology regardless of disease status or tissue type. We have added this as a new section to our manuscript "External validation in TCGA-BRCA" from lines **L520-L570** as well as new figures: **Figure 7** and **Supplementary Figure 19**.

References:

1. Brigham & Women's Hospital & Harvard Medical School Chin Lynda 9 11 Park Peter J. 12 Kucherlapati Raju 13, Genome data analysis: Baylor College of Medicine Creighton Chad J. 22 23 Donehower Lawrence A. 22 23 24 25, Institute for Systems Biology Reynolds Sheila 31 Kreisberg Richard B. 31 Bernard Brady 31 Bressler Ryan 31 Erkkila Timo 32 Lin Jake 31 Thorsson Vesteinn 31 Zhang Wei 33 Shmulevich Ilya 31 and Oregon Health & Science University Anur Pavana 37 Spellman Paul T. 37, 2012. Comprehensive molecular portraits of human breast tumours. Nature, 490(7418), pp.61-70.

2. Astashchanka, A., Shroka, T.M. and Jacobsen, B.M., 2019. Mucin 2 (MUC2) modulates the aggressiveness of breast cancer. Breast cancer research and treatment, 173, pp.289-299.

2. The validation of the gene expression prediction at the level of individual tiles is purely qualitative (Figure 5). The presented examples look convincing, but since the last part, associating local gene expressions with tissue substructure - which is in my opinion an important contribution of this work - relies on this, a quantitative validation appears necessary.

We thank the reviewer for their question. First we would like to highlight that we do not make any claims that a given tile-level prediction's scale should or could match what the equivalent piece of tissue expression for a given gene would be in reality if we had a means to measure it locally. The idea behind *RNAPath* is to use bulk-level tissue expression as a weak supervision signal to enable the association of tissue substructures, as represented by tile representations, to the expression level of a given gene measured in bulk. This allows us to infer (proportional) gene-localised morphology relationships, regardless of whether the predicted scale of expression for a given tile is correct. Indeed, most WSI have multiple repeated tissue sections, therefore this means that whilst *RNAPath* can predict the location of expression, its scale will only be proportional to the ground truth. We account for the fact that some slides have many more tissue sections or larger tissue sections than others, by normalising by the number of image tiles derived from a given WSI.

Additionally, whilst **Figure 5** is qualitative in some sense, we do believe that the fact our *in-silico* predictions of very well known substructure specific control genes highlight the very substructures they are known to be specifically expressed in, is strong evidence *RNAPath* is able to link localised tissue substructures to gene expression. Furthermore, our predictions of where these genes are expressed were also validated with independent ground truth Immunohistochemistry (IHC) results, which is considered the gold-standard for assessing cell/tissue substructure expression specificity in histological tissue specimens. We believe this represents significant evidence our method works and the tile-level predictions it makes are sound. Indeed this is a similar approach to method validation from previous publications, including the method we benchmark RNAPath against, *HE2RNA*. HE2RNA performed two IHC validation experiments, whilst we provide four IHC experiments here (Schmauch *et al.,* 2020; Nature Communications).

We do perform quantitative analyses of our tile-level predictions in the manuscript by introducing a substructure specific enrichment score (SSES). This SSES metric, for a given gene expression prediction, computes the degree of enrichment of a gene's tile-level predictions, by averaging over its predicted expression coming from tiles in a given substructure of interest, relative to everything else. For example, if we take *SLC6A19*, a well known colonic mucosa specific marker gene (see **Figure 5** for *SLC6A19* IHC and *RNAPath* prediction), it has a SSES score of 3.42. An SSES of 1 corresponds to a gene having equal expression in colonic mucosa

containing tiles versus any other tile, i.e. a gene with non-enriched, non-specific expression. The SSES score of 3.42 for *SLC6A19* ranks it 7th out of all 3319 genes regressed, recapitulating that *RNAPath* predicts it as a highly specific colonic mucosa marker. All SSES metrics for all genes across all substructures are shared as supplementary tables with numerous examples demonstrating that our tile-level predictions recapitulate known biology in terms of well understood, substructure specific gene expression.

Finally, whilst our manuscript has been in review, Mosquera *et al.,* 2023 have published, described and validated spatially, how *CRTAC1* is a biomarker of calcification and atherosclerosis progression. Interestingly, we predicted both with our differential expression analysis and our *RNAPath* predictions that *CRTAC1* is highly expressed and highly localised to calcified arterial tissue (**Figure 6, Supplementary Figure 10**). We feel that collectively these results validate *RNAPath's* spatial/tile predictions.

References:

1. *Schmauch, B. et al. A deep learning model to predict RNA-Seq expression of tumours from whole slide images. Nat. Commun. 11, 3877 (2020).*
2. *Mosquera JV, Auguste G, Wong D, Turner AW, Hodonsky CJ, Alvarez-Yela AC, Song Y, Cheng Q, Lino Cardenas CL, Theofilatos K, Bos M, Kavousi M, Peyser PA, Mayr M, Kovacic JC, Björkegren JLM, Malhotra R, Stukenberg PT, Finn AV, van der Laan SW, Zang C, Sheffield NC, Miller CL. Integrative single-cell meta-analysis reveals disease-relevant vascular cell states and markers in human atherosclerosis. Cell Rep. 2023 Nov 9;42(11):113380. doi: 10.1016/j.celrep.2023.113380. Epub ahead of print. PMID: 37950869.*

3. The main machine learning methods presented here are not new. Several works applying self-supervised learning to histology data have already been published. In particular, the choice of an ImageNet pretrained model as the baseline to compare against is debatable. The prediction of gene expression from histology slides has also been the subject of several previous articles. While the RNAPath approach shows better performance than HE2RNA (the only other method presented here), since it just consists of a set of linear models, it cannot really be described as "novel".
As such, this is not a blocking point because the downstream analyses are still interesting, but some statements should be tempered, in particular:
- L. 51-52 "with some preliminary applications focused on histopathology".

As mentioned earlier, there have already been several works focusing on self-supervised learning applied to histology data, including for instance the following references:
*** Saillard, C., Dehaene, O., Marchand, T., Moindrot, O., Kamoun, A., Schmauch, B., & Jegou, S. (2021). Self supervised learning improves dMMR/MSI detection from histology slides across multiple cancers. arXiv preprint arXiv:2109.05819.**
*** Ciga, O., Xu, T., & Martel, A. L. (2022). Self supervised contrastive learning for digital histopathology. Machine Learning with Applications, 7, 100198.**

*** Srinidhi, C. L., Kim, S. W., Chen, F. D., & Martel, A. L. (2022). Self-supervised driven consistency training for annotation efficient histopathology image analysis. Medical Image Analysis, 75, 102256.**
*** Saldanha, O. L., Loeffler, C. M., Niehues, J. M., van Treeck, M., Seraphin, T. P., Hewitt, K. J., ... & Kather, J. N. (2023). Self-supervised attention-based deep learning for pan-cancer mutation prediction from histopathology. NPJ Precision Oncology, 7(1), 35.**
*** Chen, R. J., Chen, C., Li, Y., Chen, T. Y., Trister, A. D., Krishnan, R. G., & Mahmood, F. (2022). Scaling vision transformers to gigapixel images via hierarchical self-supervised learning. In Proceedings of the IEEE/CVF Conference on Computer Vision and Pattern Recognition (pp. 16144-16155).**
- L. 87-88 "ImageNet pre-trained models which are the standard in the field". Same comment, as the field is evolving rapidly, I do not think it is right anymore to say it is the standard.
- L. 669 "Our novel method, RNAPath": I would remove "novel".

We thank the reviewer for their suggestions, especially the relevant articles referenced. We agree with the reviewer on all points in terms of additional referencing and text changes, and therefore we have incorporated all suggestions into our revised manuscript.

Additionally, the reviewer suggests (**similar to reviewer 1 Q1 and reviewer 3 Q4**) that an ImageNet baseline for representation learning in histology as a comparison is debatable. The primary reason we include a comparison to ImageNet representations is because whilst we agree with the reviewer they are not the state of the art approach anymore, they are still widely used in practice (Huang *et al.,* 2023; Azizi *et al.,* 2023; Chen *et al.,* 2022). However, we agree with the reviewer that demonstrating our representations are more performant than other self-supervised histology representation learning methods would be more convincing. Therefore, we have conducted an additional experiment that includes a comparison to CTransPath, a model pre-trained on 15 million histopathology images (Wang *et al.,* 2022) as suggested by Reviewer 3. We have updated **Figure 2 as well as Supplementary Figure 2 & 4**. Please see the answer to **Reviewer 1 Q1** for all of the results.

References:

1. Wang, X., Yang, S., Zhang, J., Wang, M., Zhang, J., Yang, W., Huang, J. and Han, X., 2022. Transformer-based unsupervised contrastive learning for histopathological image classification. Medical image analysis, 81, p.102559.
2. Huang, Z., Bianchi, F., Yuksekgonul, M., Montine, T.J. and Zou, J., 2023. A visual–language foundation model for pathology image analysis using medical twitter. Nature medicine, 29(9), pp.2307-2316.
3. Azizi, S., Culp, L., Freyberg, J., Mustafa, B., Baur, S., Kornblith, S., Chen, T., Tomasev, N., Mitrović, J., Strachan, P. and Mahdavi, S.S., 2023. Robust and data-efficient generalization of self-supervised machine learning for diagnostic imaging. Nature Biomedical Engineering, pp.1-24.

4. Chen, C., Lu, M.Y., Williamson, D.F., Chen, T.Y., Schaumberg, A.J. and Mahmood, F., 2022. Fast and scalable search of whole-slide images via self-supervised deep learning. Nature Biomedical Engineering, 6(12), pp.1420-1434.

Minor points

4. **The methodology for the weakly supervised segmentation of histology images could be clarified a little bit. In the Methods section (L. 595) it is said that "a small number of tiles from 5-10 WSI per tissue" were annotated. The authors should specify how the tiles were sampled (were they randomly chosen or selected in a way?). Even if the information is present in the supplementary material, the "small number" could be made explicit here. It is also not clear who actually performed the annotation, as the text only says (L.147) that the annotations "were validated by a clinical histopathologist".**

We thank the reviewer for their comment and completely agree that this section could have been clearer and more explicit. We have added more information to the methods section regarding how annotated tiles were selected on lines **L682-692.**

These inclusion criteria enabled us to have broad coverage of tissue substructures that are common across donors for a given tissue, whilst ensuring we can segment incidental pathology findings. These classes are not exhaustive but missing classes can be handled by the model. For example in Skin, there are specimens that have solar elastosis. Whilst we do not specifically segment this as it's a mild, uninteresting pathology (due to age and sun exposure), it modifies the appearance of the dermis. These tiles are still successfully segmented as belonging to dermis. Indeed, one use-case we wanted is that If users have additional and more specific substructure or pathologies they are interested in, it is quite easy to extend our method to use additional annotations. Finally, the gene expression predictions by RNAPath and the computation of Moran's I (spatial autocorrelation) that we present in the manuscripts supplement (**Supplementary Figure 17**) does not depend on any annotated substructures. Therefore, if genes are specific to non-annotated substructures or pathologies, and are regressed accurately, by using RNAPath we are able to identify novel, unannotated substructures via localised gene expression predictions.

5. Do all annotated patches belong to only one class? If so, what if a patch lies at the border between two histological structures?

All annotated tiles belong to a single class. When annotating, specific care by a histopathologist (A.P.L) was taken to remove ambiguously annotated tiles - for example, tiles which had a minority amount of the target class being specified. However, the reviewer is correct that we are limited by our tile resolution (128x128 pixels, ~ 63um x 63um) and therefore if a tile crosses a boundary (i.e. media and adventitia boundary in arterial tissue) an annotated tile could be labelled "adventitia" but in fact also have some medial tissue present. The way we sought to ameliorate this issue, was to sample tiles multiple times with overlap and perform majority voting

with our kNN model. This way, we can average away uncertainty for boundary cases. Indeed, **Figure 4B** highlights that we have high sensitivity even for tissue substructures that are quite narrow and spatially regionalised (colonic mucosa). We have updated our methods section to more explicitly detail this limitation and how we accounted for it, please see lines **L706-710.**

6. L.152-153, the methodology for the 10-fold cross-validation should also be clarified. In particular, did the authors ensure that patches from the same slides were not present both in the training and test set? If yes, this should be mentioned explicitly. Otherwise, the reported accuracy is probably optimistic.

We thank the reviewer for their comment. We did not ensure that tiles from the same slides were not present in both the training and the test set because, as reported in the tables below, they have been extracted from less than 10 slides for all annotated classes, making a 10-fold cross-validation unfeasible if we were to split at the level of WSI.

In the tables below (**Supplementary Table 1 and 2 for reviewer)**, we have evaluated the accuracy of tile classification by leaving tiles from the same slides either in the train or in the test set, when possible. This is not possible for the following classes: blood clot (tibial artery), erosive esophagitis and congestion (esophagus mucosa). For these cases, we randomly split the train and test set tiles annotated from a single slide).

In the majority of cases the accuracy values of slide-level splitting are comparable to the scores reported in Supplementary Table 1. Indeed, the median difference in accuracy between slide-level splitting and tile-level splitting is 0.03. The class with the largest difference is red blood cells; however, this is a class that has little biological utility, which we annotated for completeness, but we did not use it in any downstream analyses. In summary, with more extensive annotations spanning additional WSI, we believe the use of our representations to perform substructure segmentation would only improve.

Class	# Slides	# Train tiles	# Test tiles	Accuracy (slide-level split)	Accuracy (tile-level split)
Tunica intima	5	891	109	0.917	0.925
Tunica media	5	876	124	0.927	0.920
Tunica adventitia	5	903	97	0.948	0.900
Calcification	4	861	139	0.906	0.939
Atherosclerosis	2	872	128	0.727	0.943
Adipocytes	7	899	101	0.950	0.964
Nerve	2	846	154	0.870	0.982
Red blood cells	2	356	109	0.229	0.951
Blood clot	1	205	23	0.870	0.908

Supplementary Table 1 for reviewer: Number of slides and tiles used to train and test the tile classification in tibial artery samples, with the corresponding accuracy, reported when splitting

the tiles by sample (slide-wise split), versus the average accuracy of the 10-fold-cross validation experiment documented in Supplementary Table 1 (tile-wise split).

Class	# Slides	# Train tiles	# Test tiles	Accuracy (slide-wise split)	#Accuracy (tile-wise split)
Epithelium	5	904	96	0.917	0.919
Smooth muscle	4	922	78	0.846	0.873
Stroma	5	896	104	0.856	0.851
Inflammation	5	794	90	0.933	0.937
Erosive esophagitis	1	672	75	1.000	0.973
Submucosal gland	3	884	116	0.940	0.923
Congestion	1	567	63	0.952	0.886

Supplementary Table 2 for reviewer: Number of slides and tiles used to train and test the tile classification in esophagus mucosa samples, with the corresponding accuracy, reported when splitting the tiles by sample (slide-wise split), versus the average accuracy of the 10-fold-cross validation experiment documented in Supplementary Table 1 (tile-wise split).

7. L.160-165, "Tiles inferred as belonging to the calcification class had high sensitivity in recovering ground-truth pathologist labels, with AUC = 0.94 (Supplementary Figure 4), indicating that the vast majority of true positive cases were correctly identified by the kNN segmentation model": AUC is not the right metric to evaluate the model's sensitivity and the number of true positives.

We are unsure why the reviewer thinks our AUROC TPR/FPR analysis presented in **Supplementary Figure 5** is not appropriate. We are considering a binary classification task, in which given a threshold of calcification predicted to be in a WSI, we assign the label “calcified” or “healthy”. AUROC is the standard metric in the field for evaluating binary classification performance. However, for clarity, we have now also provided the number of True Positives (TPs) as well as sensitivity (89.7%) and specificity calculations (79.0%) . When re-running the analysis, we discovered that a group of samples we discarded as they had NaN values in the pathology notes (which makes the string search incomplete), had instead to be considered healthy. The AUROC went from 0.94 to 0.93 and the number of samples with detected calcification but no calcification reported went from 4 to 8. We have updated the text accordingly on lines **L180-190.**

8. In section "Sex and age-specific variability in tissue substructure and pathological features" (L. 234), the statistical test used should be mentioned, as well as whether any correction was applied for multiple hypothesis testing.

We have now specified in the main text that we used linear models to assess the association between derived substructure proportions and different covariates. All reported p-values are Bonferroni corrected and we have updated our methods section to reflect this by adding a new section: lines: **L712-717.**

9. P-values, confidence intervals or standard deviations of cross-validation results should be reported, for instance
- L.154-155 and supp. table 1
- When describing RNAPath results (in the text and supp. table 3). It would also be interesting to report how many genes are regressed with a significant p-value.

We thank the reviewer for their suggestion. First, we noticed a mistake in which we were reporting # genes regressed with r > 0.8. However, this should have been r > 0.75 and therefore we have updated the manuscript. This did not have any impact on downstream analysis.

We agree with the reviewer that we should have included SDs for the cross-validation results and have updated **Supplementary Table 1** to include this. Additionally, we also agree that reporting the number of significant genes regressed by *RNAPath* is also of interest. We have now included this in **Supplementary Table 3, where we report the number of FDR 1% significant genes.** We reproduce below for the reviewers convenience:

Tissue	Median r score	#Genes r > 0.50	#Genes r > 0.75	#Genes regressed	#Significant genes (FDR1%)
Adipose Tissue	0.25	946	2	11,413	3,761
Aorta	0.22	629	9	11,591	2,169
Atrial Appendage	0.40	2,195	58	8,857	5,039
Colon (Transverse)	0.44	4,948	590	12,002	6,982
Colon (Sigmoid)	0.33	2,356	127	11,683	4,178
Coronary Artery	0.19	1,517	136	12,163	834
Esophagus Mucosa	0.48	4,869	278	10,975	8,575
Esophagus Muscularis	0.35	2.598	123	11,014	4,888
Gastroesophageal Junction	0.46	4,866	639	11,217	6,341
Heart	0.65	6,097	1,384	7,538	6,894
Lung	0.35	2,983	224	13,115	6,819
Mammary Tissue Breast	0.39	3,327	129	12,471	6,118
Omentum	0.37	2,067	4	11,582	6,449
Pancreas	0.58	5,042	378	7,370	5,953
Pituitary Gland	0.13	475	3	12,788	705
Skeletal Muscle	0.52	4,516	289	8,305	7,425
Skin (Sun Exposed)	0.20	138	0	11,538	2,565
Skin (Suprapubic)	0.21	187	0	11,514	2,712
Stomach	0.39	3,174	36	11,530	5,093
Testis	0.50	7,473	960	14,994	9,798
Thyroid Gland	0.36	2,817	49	12,970	7,778
Tibial Artery	0.31	1,648	73	11,282	5,196

Tibial Nerve	0.19	298	5	12,614	2,313

Supplementary Table 3: Summary of *RNAPath* results across tissues in the test set (median correlation, number of genes with correlation coefficient r > 0.5 and r > 0.75, total number of regressed genes and their FDR1% significance).

10. When reporting the IoU of well predicted genes between different tissues, again the significance of the result should be assessed more rigorously (L. 387-388 and supp. fig. 12). In particular, how many genes were predicted in both tissue types, and what is the p-value of the result.

We thank the reviewer for their comment and agree that the IoU tissue sharing results would be stronger if presented with a statistical measure of significance. To do this, we have quantified the statistical significance of gene sharing between two tissues with high IoU (gastroesophageal junction and esophagus mucosa) versus two tissues with a low IoU and are biologically unrelated (pituitary gland and omentum). To do this, we performed a hypergeometric test, assessing the significance of well-regressed gene sharing between tissues, given the universe of expressed genes (i.e. genes expressed in either tissue). For gastroesophageal junction and esophagus mucosa we see that 837 genes are shared and well regressed in both tissues (P-value = 1.38×10^{-6}) where as only 11 genes are shared and well-regressed for pituitary and omentum (P-value = 0.71). This highlights that for biologically related tissues, genes that *RNAPath* can regress accurately are more likely to overlap as compared to unrelated tissues. We believe this is a fairly intuitive result. We have updated the text in the paper, to quote both the number of overlapping genes and the significance of each result. Please see lines **L428-433.**

11. L. 398-399, "Surprisingly, we see high concordance between our spatially resolved RNA expression predictions and that of matched antibody staining": I am not sure the word "surprisingly" is appropriate here, as similar results were shown in
*** Fu, Y. et al. Pan-cancer computational histopathology reveals mutations, tumor composition and prognosis. Nat Cancer 1, 800-810 (2020)**
*** Schmauch, B. et al. A deep learning model to predict RNA-Seq expression of tumours from whole slide images. Nat. Commun. 11, 3877 (2020).**

We thank the reviewer for their comment and we agree that based on the two papers cited, we have removed the word “surprisingly”. In addition, we have included the missing reference mentioned in our revised manuscript.

Reviewer #3 (Remarks to the Author):

This article addresses an interesting and timely concept. The authors use self-supervised learning (SSL) to train a foundation model for tissue image analysis. The approach is interesting

and the paper could be a good fit for this journal, but there are a number of fundamental issues which need to be fixed.

We thank the reviewer for acknowledging the timely nature of our manuscript and how it would be a good fit for Nature Communications. We hope to have addressed the reviewers' concerns below. In summary we have:

1. Included an additional experiment that benchmarks our DINO representations against CTransPath and we demonstrate that our representations outperform not only Imagenet pretrained models, but also CTransPath (43**% higher silhouette score**).
2. We have significantly improved the manuscripts scholarship by including relevant references kindly provided by the reviewer.
3. We have modified and improved all figures by adding scale bars, units and subplot labels.
4. We have cut text whilst keeping the scientific message clear
5. We have clarified that we are not quantitatively interpreting the UMAP plots, but the underlying representations learnt from DINO.

We believe the manuscript is now significantly better having addressed all reviewers comments and have included line by line answers below.

1. The abstract is very poorly written. Even for a researcher in this field, the abstract is too convolved and poorly understandale. This is exacerbated by the fact that the authors do not define abbreviations such as GTEx or eQTL. Also, the abstract is too long and poorly structured. The abstract needs to be completely rewritten to provide an adequate background, information on the methods and technical novelty, then to provide quantitative (!) data and short interpretation of the findings.

We thank the reviewer for their suggestions. We have reduced the amount of text in the abstract significantly, substantially rewritten it and defined abbreviations. It is now < 200 words, which is within the Nature editorial guidelines.

2. the results section is really long and convoluted and poorly structured.

We apologise that the reviewer found our results section to be too long and poorly structured. Whilst no other reviewer had the same concern, we have taken this advice onboard and reduced the amount of text whilst conserving the scientific message. As the reviewer does not suggest any specific changes to be made to the structure, we would be happy to receive those specific suggestions if they feel it still reads poorly.

3. The authors use DINO but this is not state of the art anymore. DINO-v2 has been out for quite some time and is far superior. Other studies, such as the recent Nature paper on a retinal photograph foundation model by the Keane lab have systematically evaluated multiple SSL methods. This needs to be addressed by additional experiments.

We thank the reviewer for their comment. We are aware of DINOv2. When we submitted this manuscript to Nature Communications (22nd August 2023), **DINOv2 had only been published for 4 months (14th April, 2023)** and all analysis for this manuscript had already been completed. Given the rapid development of the field, we feel it is absolutely normal that new methods will be released and improved upon, but it is unrealistic to expect authors to repeat all of their analyses if a newer version is released when significantly into their analyses. Primarily, it should be noted that DINOv2 would not fundamentally change any of our conclusions, it would perhaps lead to slightly better performance in terms of representation clustering (higher silhouette scores) and gene expression prediction (higher median R^2 scores) if we extrapolate from its performance on natural images presented in the DINOv2 manuscript. This would be a nice further development in the future, but does not impact our scientific & biological conclusions in any substantial manner. However, we have now included it as a topic in our Discussion from lines **L610-615.**

4. The statement that "previous ImageNet pre-trained models" are the standard in the field is simply untrue. For more than a year, publicly available models which are pre-trained with SSL are available, e.g. RetCCL and Ctranspath. Dozens of studies have been published using these SSL-trained models. Recent papers on new SSL-trained models (please check recent papers by the Mahmood lab at Harvard, by Owkin and by Thomas Fuchs) have compared their performance to Ctranspath. The authors only compare to imagenet-trained models and thereby choose an unrealistically low-quality benchmark. This needs to be addressed by additional experiments.

We thank the reviewer for their comment. However, the primary reason we included a comparison to ImageNet representations was because even though it is not state of the art anymore, it is still very widely used in computational pathology (Huang *et al.,* 2023; Azizi *et al.,* 2023; Chen *et al.,* 2022). Additionally, upon submission of our manuscript (both Chen *et al.,* 2023 and Vorontsov *et al.,* 2023 that the reviewer cites, were preprinted whilst this paper was in review). However, we do recognise that this field moves incredibly quickly and large foundation histopathology models have now been released, such as CTransPath. Therefore, we have conducted an additional experiment as recommended by the reviewer that includes a comparison to CTransPath, a multi-scale Swin-transformer pre-trained on 15 million histopathology images. We have updated **Figure 2, Supplementary Figure 2 and Supplementary Figure 4** to reflect this, demonstrating our representations outperform CTransPath. Please see the full results in the answer to **Reviewer 1 Q1.**

5. The figures have a suboptimal visual quality and there are some typos in the figures, e.g. "Features extraction" instead of "Feature extraction". Also, abbreviations in the figures need to be explained in the legend. Finally, all histopathology images in the figures require scale bars as required by the journal's author guidelines.

We thank the reviewer for pointing out the typo. We have corrected “Features extraction” to “Feature extraction” and have added all abbreviations to the figure legends, labeled subplots, as

well as adding scale bars to the histology images. With these news changes, we disagree with the reviewer that the figures have a suboptimal visual quality and we ensured they communicate our results clearly. However, we would be happy to take further specific suggestions from the Nature Communications editorial team if deemed detrimental to the scientific understanding of the manuscript.

6. Figure 2,3,4,5,6,7 need panel labels A, B, C and scale bars and a legend for the color code and color scale bars with unit labels for the heat maps which are shown

We agree with the author that the addition of panel labels makes referring to the figures in the text much clearer. We have modified all the figures in the manuscript with respect to the reviewers suggestions.

7. UMAP plots should not be quantitatively interpreted. UMAP is sensitive to hyperparameter choices.

We thank the reviewer for their comment. However, it should be noted that **we do not at any point quantitatively evaluate the UMAP**. The UMAP is presented purely for *qualitative* purposes to visualise the clustering of tissue substructures that are separable given our self-supervised representations. **The quantitative silhouette score metric reported, is computed on the self-supervised representations** themselves, not on the 2D UMAP embeddings. We have modified the text to make this much clearer on lines **L164-168** and in the methods section “Weakly supervised segmentation of histology images” **L704-706**.

8. the authors cite only 42 references which is not a lot for this massive article. The authors omit a few relevant references on the preidciton of gene expression from pathology slides (e.g. https://pubmed.ncbi.nlm.nih.gov/35143898/ and https://www.nature.com/articles/s41467-020-17678-4 and https://www.nature.com/articles/s43018-020-0087-6 and https://www.thelancet.com/journals/ebiom/article/PIIS2352-3964(21)00181-X/fulltext and several others)

We thank the reviewer for supplying very relevant citations and we agree that a more comprehensive list of references could have been used. We note that https://www.nature.com/articles/s41467-020-17678-4 was already cited in our manuscript and forms the basis of our *RNAPath* benchmarking experiment. We have now included the additional missing articles suggested by the reviewer and performed a more comprehensive literature review bringing the total number of references up to 58.

REVIEWER COMMENTS

Reviewer #1 (Remarks to the Author):

Re Q1: I welcome the addition of benchmarking with CTransPath. I have a few questions remaining about the current benchmarking

The performance difference with CTransPath needs to be put in context. CTransPath is trained on tumour WSIs, which have a much lower proportion of normal tissue architectures. It's not surprising that it performs worse on GTEx compared to the authors' model which was trained on GTEx. It would be helpful to use the results to highlight the shortcomings of modern architectures trained only on tumours, which have different histomorphological phenotypes. Therefore, training state-of-the-art architectures on normal tissue WSIs is valuable.
There are methods directly trained on GTEx. Here are two examples including one published in this venue. https://www.nature.com/articles/s41467-021-21727-x and https://journals.plos.org/ploscompbiol/article?id=10.1371/journal.pcbi.1006269. Both are not modern architectures, but more accurately reflect the state-of-the-art in normal tissue.
I suggest comparing classification performance (linear/kNN) on those tiles with annotations to complement silhouette scores.
I respect the authors' decision to keep ImageNet ResNet50 in the benchmarking, and also OK if the authors eventually decide to omit it from further benchmarking.

Re Q2: I thank the authors for clarifying all of the details of cross-validation. I think the key question that should be addressed here is the generality of the encoder, meaning how it performs on data that is completely outside of its training phase. This means I'm suggesting training an encoder from scratch in each training set. I appreciate this would be a lot more computing overhead. I would suggest doing a 5-fold, rather than 10-fold cross-validation.

Re Q3-Q6: The authors have addressed these questions.

Re Q7: The added 5-fold results are very helpful. I'd suggest adding one or two 5-fold results on "typical" sample sizes.

Reviewer #2 (Remarks to the Author):

I acknowledge the fact that the authors did provide an effort to improve the manuscript in light of the reviews. They compared their model to another SSL feature extractor (CTransPath) and applied it on external data (TCGA-BRCA). However in my opinion it is still not sufficient. In particular, the comparison of feature extractors is done only on the GTEx database that was used for training the authors' SSL model, meaning that it cannot be considered fair to other feature extraction methods. This remains perhaps the biggest flaw of the current work. Also, a few statements in the manuscript still do not rely on any proper quantitative evaluation. Overall, I think revision is still required at this stage.

1. While it is true that GTEx is a multi-organ, multi-centric cohort, it does not mean that there cannot be any domain shift between this dataset and others. There is also no guarantee that the model is not "overfitting" the data, even if not trained in a supervised way. The transfer to an external cohort (TCGA-BRCA) is a welcome step to demonstrate the robustness of the model. However, comparison with other extraction methods should also be done on external data. More generally, fair comparison between various feature extractors should ideally be performed on data that was used to train one of them.

2. Regarding self-supervised WSI tissue substructure and pathology segmentation, I thank the authors for clarifying the cross-validation scheme, however the Supplementary Tables 1 and 2 for reviewer raise 2 remarks:

- What does the column Accuracy (slide-level split) mean when there is only one annotated slide?

- Reporting the median difference between the 2 methods is a weird choice, since it hides the fact that the accuracy drops dramatically for certain classes, in particular Red blood cells. The authors say this class has little biological utility, but there is no guarantee that the same drop does not happen for other, critical classes, in tissues other than tibial artery and esophagus mucosa. The average difference between the 2 validation schemes in tibial artery (excluding Blood clot for which there is only one annotated slide) is 0.13 (0.809 for the slide-level split, 0.941 for the tile-wise split).

Therefore, I am not convinced that this matters little, and the authors should report metrics from a slide-wise split - at least for classes for which it is possible in supp. table 1 and when reporting the median accuracy (l.173-175). Metrics from the tile-wise cross-validation can still be reported, but it has to be clarified in the text or in the table.

3. Regarding Supp. Fig. 19, I think the analysis should go beyond the statement "we see that tile representations of breast-cancer relevant annotations cluster into distinct groups". For instance, silhouette scores should be reported as on GTEx. Also, while the results seem to be rather satisfactory from the figure, the clustering is (unsurprisingly) not as "clear" as in GTEx. In particular, some structures are divided into several sub-clusters, while nerves appear to be intertwined with stroma, vessels, and sometimes adipocytes. The authors should elaborate on that.

4. The sentence "we see that areas of carcinoma are well segmented from benign tissue" (l. 528-529) does not refer to any quantitative metric. AUC and/or sensitivity/specificity should be reported to support this statement. Generally speaking, the validation of the feature extractor on TCGA-BRCA through weakly-supervised segmentation lacks precision.

5. It is difficult to conclude anything from the survival analysis carried out on TCGA-BRCA (l. 532-535). The idea that the amount of tumor on the slide is linked to prognosis seems reasonable, but, even if the p-value is significant, the separation between the two subgroups is not extremely convincing (the confidence intervals of both subgroups overlap). Also, what happens to the intermediate patients (those with a tumor percentage between the 25th and the 75th percentile)? If there is no strong message here, I suggest simply removing this part.

6. L. 548, the statement "we see RNAPath is able to predict known marker genes, for example, PLIN1 in adipocytes" should be backed by a metric. Since it is the only true validation of RNAPath on external data (the model is finetuned afterward), this part should be developed more.

7. If CCNE1 and PGR expressions are predictors of the subtype, in a sense the model has access to the subtype information: if the model is able to predict the expression of CCNE1 and PGR, it is able to predict the subtype, and conversely. I do not think it is an issue as such, but I would remove "despite having access to no subtype information" (l. 558-559).

8. Finally, regarding the prediction of local gene expression, the authors say they "do not make any claims that a given tile-level prediction's scale should or could match what the equivalent piece of tissue expression for a given gene would be in reality if we had a means to measure it locally". This seems contradictory with what is written l. 435-439: "As well as bulk level predictions, RNAPath provides tile level (128×128 pixel) expression predictions which can be used to create spatial expression maps of any specific across a histology sample."

Moreover, in the comparison with IHC, the authors refer to it as the "ground truth", but I find the term

misleading. From a Machine Learning perspective, I would expect the “ground truth” to refer to the spatial gene expression (which could be obtained from spatial transcriptomics or double staining or adjacent sections with H&E and IHC) on the very same sample. Here, the predicted gene expression is compared to the IHC marker on completely different samples. While it highlights the fact that the model does associate the expression of the 4 selected genes to relevant histological structures, I insist it is a qualitative validation.

Altogether, I think this part should be rewritten to clarify what exactly the experiments are probing.

Reviewer #3 (Remarks to the Author):

Thanks for addressing my concerns, no further comments

REVIEWER COMMENTS

Reviewer #1 (Remarks to the Author):

Re Q1: I welcome the addition of benchmarking with CTransPath. I have a few questions remaining about the current benchmarking

The performance difference with CTransPath needs to be put in context. CTransPath is trained on tumour WSIs, which have a much lower proportion of normal tissue architectures. It's not surprising that it performs worse on GTEx compared to the authors' model which was trained on GTEx. It would be helpful to use the results to highlight the shortcomings of modern architectures trained only on tumours, which have different histomorphological phenotypes. Therefore, training state-of-the-art architectures on normal tissue WSIs is valuable.
There are methods directly trained on GTEx. Here are two examples including one published in this venue. https://www.nature.com/articles/s41467-021-21727-x and https://journals.plos.org/ploscompbiol/article?id=10.1371/journal.pcbi.1006269

Both are not modern architectures, but more accurately reflect the state-of-the-art in normal tissue.
I suggest comparing classification performance (linear/kNN) on those tiles with annotations to complement silhouette scores.
I respect the authors' decision to keep ImageNet ResNet50 in the benchmarking, and also OK if the authors eventually decide to omit it from further benchmarking.

We thank the reviewer for their question. We are also happy that they welcome the additional benchmarking of our method against another self-supervised learning approach, CTransPath, as suggested by Reviewer 3. We agree with the reviewer about setting the right context for CTransPath and how it was trained; We have therefore updated the manuscript at lines L577-L584 accordingly to reflect this. Indeed, it is often tricky to obtain a fair comparison, with the majority of modern SSL models having been trained on cancer histology, whilst ours is trained on normal histology with incidental findings at autopsy.

The reviewer suggests two papers that have been trained on subsets of GTEx. One is a simple Convolutional Autoencoder (CAE) (Ash *et al.,* 2021), whilst the other paper (Bizeggo *et al.,* 2019) focuses on benchmarking VGG, InceptionV3 and Resnet, three relatively old ImageNet pretrained models. The authors fine-tune these pre-trained models using only 50,000 GTEx image tiles (maximum of 100 tiles per tissue type) - versus our 1.7 million images used for training. They use a final linear projection layer of dimension 1000 for their representations.

Unfortunately for both approaches, the authors provide no model weights on their github repository, making their analyses difficult for us to reproduce - This is compounded by the fact

the last git commit for Ash *et al.,* 2021 was 6 years ago. Furthermore, we feel it is unlikely that a CAE architecture from 2017 trained on GTEx release version 6p, and a Resnet trained on 50k GTEx tiles, would lead to better representations than a ViT-S trained with DINO (our approach) on a later GTEx release (v8) with 34x more data. However, we acknowledge these are the two alternative methods trained on normal histology and have therefore cited Bizzego *et al.,* 2019 along with Ash et al., 2021.

We agree with the reviewer that a nice additional comparison of our model would be to compare the classification performance on tiles with annotations as well as computing the silhouette metric for tissue substructure clustering. Therefore we computed the average kNN accuracy for all annotations from tibial artery, esophagus mucosa and colon, the same tissues used for **Figure 2** & **Supplementary Figure 4**. We see that the kNN classification accuracies using DINO embeddings are higher on average in all cases and DINO has the highest accuracy across 18 of the 21 annotations tested:

**Tissue**	**DINO**	**CTransPath**	**Resnet (ImageNet)**
Tibial Artery	**0.937** ± 0.027	0.897 ± 0.031	0.757 ± 0.115
Esophagus Mucosa	**0.909** ± 0.042	0.888 ± 0.053	0.786 ± 0.086
Colon	**0.956** ± 0.030	0.949 ± 0.022	0.907 ± 0.030

Table for reviewer: Comparison of mean accuracies across all models for annotated tile classification.

Finally, we have included in **Supplementary Table 2** the CTransPath classification accuracies, reproduced below with Resnet50-ImageNet and DINO results for the reviewers convenience. We hope these two sets of results convince the reviewer that our method choice is sound and our downstream inferences are valid.

**Tissue**	**Phenotype**	**Accuracy (tile-level split)**		
		**ImageNet**	**CTransPath**	**DINO**
Artery (Tibial)	Tunica intima	0.640 ± 0.051	0.882 ± 0.026	**0.925 ± 0.023**
	Tunica media	0.867 ± 0.031	0.926 ± 0.031	0.920 ± 0.024
	Tunica adventitia	0.785 ± 0.017	0.866 ± 0.033	**0.900 ± 0.038**
	Calcification	0.707 ± 0.067	0.901 ± 0.022	**0.939 ± 0.014**
	Atherosclerosis	0.692 ± 0.033	0.888 ± 0.039	**0.943 ± 0.019**
	Adipocytes	0.851 ± 0.030	0.956 ± 0.022	**0.964 ± 0.020**
	Nerve	0.873 ± 0.034	0.917 ± 0.031	**0.982 ± 0.015**
	Red blood cells	0.847 ± 0.073	0.873 ± 0.054	**0.951 ± 0.032**
	Blood clot	0.551 ± 0.095	0.864 ± 0.085	**0.908 ± 0.060**
Colon (Transverse)	Mucosa	0.914 ± 0.025	0.955 ± 0.025	**0.979 ± 0.018**
	Submucosa	0.894 ± 0.030	0.926 ± 0.024	**0.960 ± 0.024**
	Mucus	0.952 ± 0.013	0.983 ± 0.014	**0.987 ± 0.012**

	Muscularis	0.904 ± 0.032	0.937 ± 0.024	**0.941 ± 0.019**
	Adipocytes	0.870 ± 0.040	0.942 ± 0.015	0.914 ± 0.021
Esophagus Mucosa	Epithelium	0.788 ± 0.041	0.902 ± 0.032	**0.919 ± 0.038**
	Smooth muscle	0.845 ± 0.028	0.871 ± 0.023	**0.873 ± 0.045**
	Stroma	0.768 ± 0.038	0.815 ± 0.047	**0.851 ± 0.038**
	Inflammation	0.845 ± 0.028	0.880 ± 0.030	**0.937 ± 0.027**
	Erosive esophagitis	0.884 ± 0.051	0.991 ± 0.011	0.973 ± 0.026
	Submucosal gland	0.745 ± 0.047	0.888 ± 0.032	**0.923 ± 0.027**
	Autolysed mucosa	0.624 ± 0.063	0.870 ± 0.039	**0.886 ± 0.027**

Supplementary Table 2: Accuracy of tile classification across tibial artery, transverse colon and esophagus mucosa tissues using embeddings from a ResNet50 trained on ImageNet, CTransPath and DINO.

Re Q2: I thank the authors for clarifying all of the details of cross-validation. I think the key question that should be addressed here is the generality of the encoder, meaning how it performs on data that is completely outside of its training phase. This means I'm suggesting training an encoder from scratch in each training set. I appreciate this would be a lot more computing overhead. I would suggest doing a 5-fold, rather than 10-fold cross-validation.

Unfortunately we cannot justify the expense of the experiment requested. To put it into perspective, ViT-S DINO training on all GTEx WSI took approximately 12 days utilizing 8 GPUs across two HPC nodes. The reviewers request amounts to either 50 days of continuous model training assuming sequential computation, or, 40GPUs utilized in parallel for 5 folds. Our approach of using a single validation set is the same as the original DINO paper (Caron *et al.,* 2021), despite FacebookAI having substantially more compute available than us. As the reviewer wants additional evidence that our encoder shows generality, we would put emphasis on our TCGA breast carcinoma external validation, in which our encoder **was not fine-tuned** on TCGA, but still is able to cluster carcinoma and normal tissue specific substructures, demonstrating strong generalization across datasets - Particularly impressive given our model was trained on normal tissue histology and not tumours, therefore demonstrating the generality of the encoder to out of distribution datasets.

Re Q3-Q6: The authors have addressed these questions.

We thank the reviewer for their suggestions and are happy that we were able to answer all questions listed as the manuscript is much better for it.

Re Q7: The added 5-fold results are very helpful. I'd suggest adding one or two 5-fold results on "typical" sample sizes.

We thank the reviewer for the question and we agree that performing a 5-fold cross validation on a tissue with a “typical” sample size is a useful comparison.

We have added the following to our manuscript (L425-432) as well as updated **Supplementary Figure 13**:

*“We measured the robustness of the model through a 5-fold cross validation on three tissues, respectively with smallest (coronary artery, n = 239), median (breast, n = 456) and largest (skeletal muscle = 797) sample size. The accuracy of predictions grows linearly with the dimension of training set (median r score = 0.181 ± 0.028 for coronary artery, 0.346 ± 0.038 for breast and 0.484 ± 0.021 for skeletal muscle) as well as the model robustness, with the standard deviation of gene correlation coefficients across folds having a negative correlation with sample size (0.187 ± 0.069 for coronary artery, 0.128 ± 0.051 for breast and 0.082 ± 0.033 for skeletal muscle) both in validation and test set (**Supplementary Figure 13**).”*

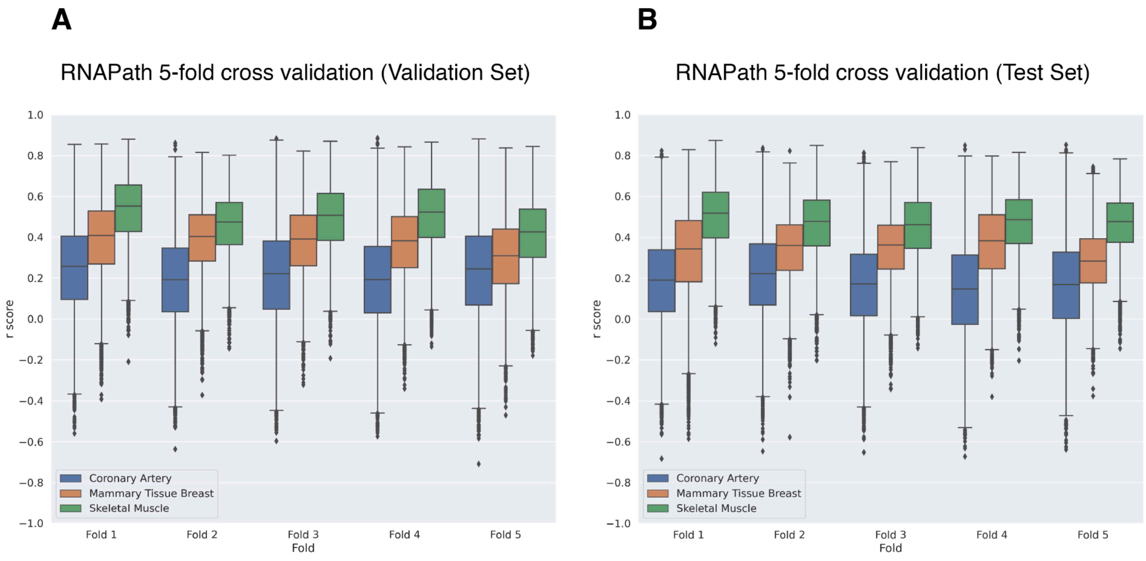

Supplementary Figure 13: RNAPath 5-fold cross validation on validation (A) and test set (B) across the smallest sample size(Coronary Artery), median sample size (Breast) and the largest sample size (Skeletal Muscle).

We thank the reviewer for this suggestion as we feel this completes the analysis, demonstrating that *RNAPath* performance scales with sample size.

Reviewer #2 (Remarks to the Author):

I acknowledge the fact that the authors did provide an effort to improve the manuscript in light of the reviews. They compared their model to another SSL feature extractor (CTransPath) and applied it on external data (TCGA-BRCA). However in my opinion it is

still not sufficient. In particular, the comparison of feature extractors is done only on the GTEx database that was used for training the authors' SSL model, meaning that it cannot be considered fair to other feature extraction methods. This remains perhaps the biggest flaw of the current work. Also, a few statements in the manuscript still do not rely on any proper quantitative evaluation. Overall, I think revision is still required at this stage.

1. While it is true that GTEx is a multi-organ, multi-centric cohort, it does not mean that there cannot be any domain shift between this dataset and others. There is also no guarantee that the model is not "overfitting" the data, even if not trained in a supervised way. The transfer to an external cohort (TCGA-BRCA) is a welcome step to demonstrate the robustness of the model. However, comparison with other extraction methods should also be done on external data. More generally, fair comparison between various feature extractors should ideally be performed on data that was used to train one of them.

We agree with the reviewer that even though GTEx is a multi-organ and multicentric cohort, it is still better to externally validate our approach on new data. This is exactly what we did with the TCGA-BRCA experiments previously reported in our initial response, in which we demonstrate that our DINO representations generalize well to a held out dataset and disease, despite it being an extreme example (normal histology versus carcinoma).

The primary purpose of our research was not to compare different feature extraction methods across different datasets, but to showcase how a self-supervised learning model can be used to derive interesting phenotypes from histology samples for downstream differential expression analysis, incidental pathology discovery, epidemiological comparison and genetic association analysis. We therefore think it would be a suitable, additional paper, to rigorously benchmark multiple different feature extraction methods in normal, cancer and other histological datasets, to determine the best one, but it is both out of scope and would distract from the main messages presented in this manuscript. Importantly, whether we benchmark other SSL methods on TCGA or not, the fundamental findings of our manuscript do not change. Furthermore, benchmarking SSL models is quite a difficult task, as the majority of SSL algorithms to date have been trained on TCGA and not on normal histology WSI. Therefore setting up a fair comparison would likely require training models from scratch to avoid the opposite problem of models being more performant on TCGA. Finally, to add additional clarity, we have expanded the discussion (L691-L698) to clearly state that we do not think our approach is necessarily the optimal approach to extract features from every digital pathology cohort, but rather that SSL approaches also achieve high performance on healthy tissue histology where most work has been focused (and trained) on oncology data. We have added the following lines to our discussion:

"Second, whilst we benchmark our SSL representations, it is entirely possible that a model trained on a specific disease cohort (e.g. Inflammatory Bowel Disease, or a specific tumour)

would outperform our approach. This is not surprising, as each disease has its own characteristic pathology that a model may only observe in that isolated disease context."

Reviewers follow up response via email:

"I agree on the fact that a rigorous benchmark of SSL methods would be outside of the scope of this work. I also agree that a fair comparison with CTransPath seems unfeasible since CTransPath was trained on TCGA. What I suggest is showing at least the comparison with ImageNet on TCGA-BRCA, to confirm that the model still performs better than the most simple baseline outside of its training domain. I leave it up to the authors to decide if a comparison with CTransPath on TCGA-BRCA is relevant. I note that they make a claim (that CTransPath would be only "marginally better") that is currently not supported by evidence. If they decide not to show this comparison, they should formally acknowledge the limitation of the benchmark performed here."

We thank the reviewer for their understanding and clarification. Therefore, in order to have a complete comparison between all methods, we evaluated ImageNet and CTransPath derived representations on the TCGA-BRCA cohort used to externally validate RNAPath. From the clustering of annotated patches (**Supplementary Figure 19**) and the median silhouette scores we see that DINO outperforms ImageNet substantially (0.159 vs 0.084, +89%); however, the silhouette score is marginally higher for SSL-CTransPath than DINO (0.179 vs 0.159), which is to be expected given CTransPath was trained on TCGA. Our DINO representations therefore show impressive generalisation.

The relative difference between DINO and CTransPath silhouette scores on TCGA (+13%) is much smaller than that on GTEx, where DINO yields a patch clustering silhouette score for example in Tibial Artery of +43% and +63% in Esophagus Mucosa.

We clarified these results in lines L577-L584 and updated our supplementary figure 19..

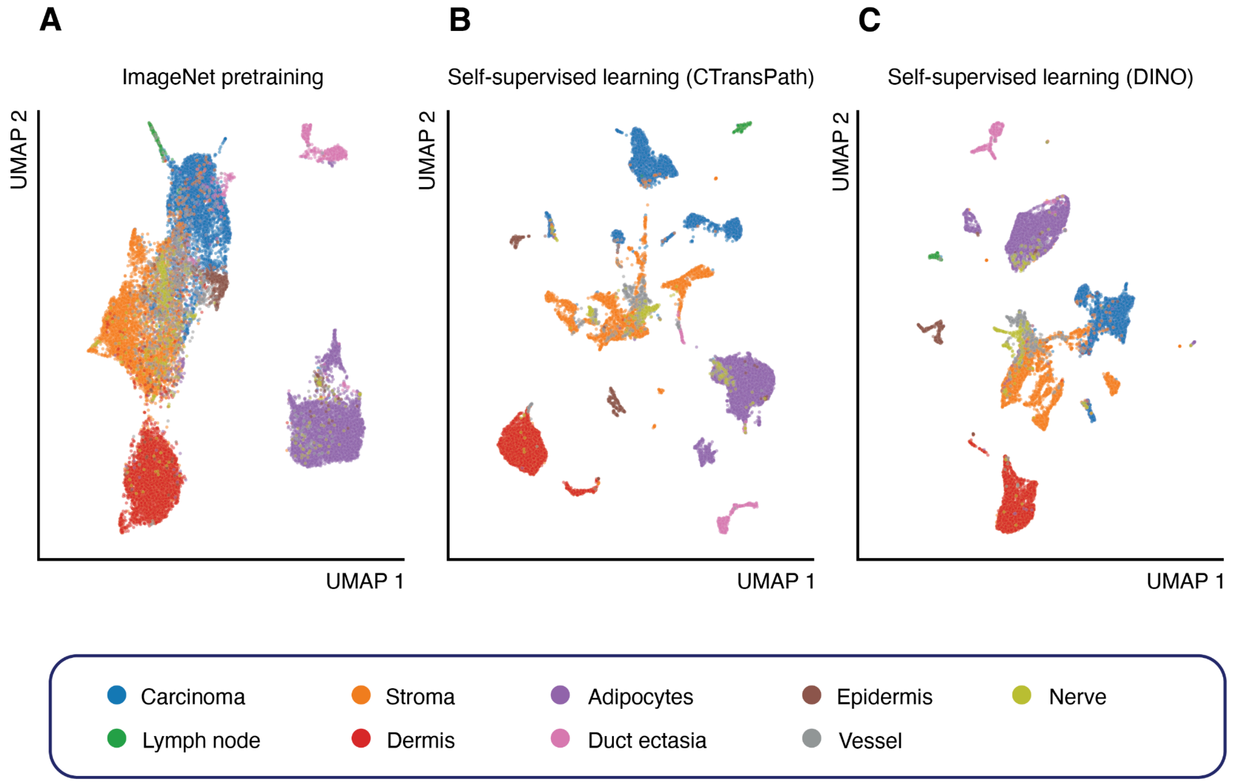

Supplementary Figure 19: UMAP embeddings of BRCA tile features from ResNet50-ImageNet (A), CTransPath (B) and the DINO model trained on GTEx.

2. Regarding self-supervised WSI tissue substructure and pathology segmentation, I thank the authors for clarifying the cross-validation scheme, however the Supplementary Tables 1 and 2 for reviewer raise 2 remarks:

- What does the column Accuracy (slide-level split) mean when there is only one annotated slide?

For the table initially prepared for the first response to the reviewer, slide-level split accuracy when only one annotated slide was considered was (and has to be) tile-level split for that annotation. We wrote in the initial response:

“For these cases [annotations from one WSI], we randomly split the train and test set tiles annotated from a single slide.“

- Reporting the median difference between the 2 methods is a weird choice, since it hides the fact that the accuracy drops dramatically for certain classes, in particular Red blood cells. The authors say this class has little biological utility, but there is no guarantee that the same drop does not happen for other, critical classes, in tissues

other than tibial artery and esophagus mucosa. The average difference between the 2 validation schemes in tibial artery (excluding Blood clot for which there is only one annotated slide) is 0.13 (0.809 for the slide-level split, 0.941 for the tile-wise split).

Therefore, I am not convinced that this matters little, and the authors should report metrics from a slide-wise split - at least for classes for which it is possible in supp. table 1 and when reporting the median accuracy (l.173-175). Metrics from the tile-wise cross-validation can still be reported, but it has to be clarified in the text or in the table.

Below, we present the results for the slide-level split, as asked by the reviewer. As you can see, for many classes, we report similar slide-level split accuracies to our original tile-level split. For some classes, especially those with only 2 WSI annotated, performance is substantially worse in the slide-level analyses. This is expected due to very few annotations having been performed across multiple subjects.

However, and most importantly, all of our downstream analysis presented in the manuscript, conducted on a small subset of these annotations, **have similar tile-level and slide-level split accuracies**. For example, the results of **Figure 4**, focusing on calcification, colonic mucosa and adipocytes, the **mean** difference between tile level and slide-level split accuracy is -1.5%. In **Figure 5**, for the annotations analyzed, the mean accuracy is actually higher for the slide-level split: +1.3%. Finally, in **Figure 6**, for the differential expression analysis presented, the mean difference in accuracy across annotations is negligible, at -0.5%. Therefore, we would like to stress that despite some annotation accuracies being significantly lower using a slide-level split, **none of our primary scientific results change based on these analyses.**

We agree with the reviewer that the slide-level split is the most rigorous approach and therefore we have extended the discussion of our paper to emphasize that users of our model can expect more accurate results proportional to the number of WSI annotated. Additionally, we have clarified in the legend of the table, the difference between tile-level and slide-level splits. For completeness, we have reported the mean (rather than median) tile-level and slide level split in the manuscript. See lines L179-L181.

Tissue	Phenotype	Annotated Tiles	Slides	Accuracy (tile-level split)	Accuracy (slide-level split)
Artery (Tibial)	Tunica intima	1,038	5	0.925 ± 0.023	0.917
	Tunica media	5,365	5	0.920 ± 0.024	0.927
	Tunica adventitia	3,172	5	0.900 ± 0.038	0.948
	Calcification	1,058	4	0.939 ± 0.014	0.906
	Atherosclerosis	1,780	2	0.943 ± 0.019	0.727
	Adipocytes	5,297	7	0.964 ± 0.020	0.950
	Nerve	1,376	2	0.982 ± 0.015	0.870
	Red blood cells	465	2	0.951 ± 0.032	0.229
	Blood clot	228	1	0.908 ± 0.060	-

Tissue	Phenotype	Annotated Tiles	Slides	Accuracy (tile-level split)	Accuracy (slide-level split)
Colon (Transverse)	Mucosa	2,680	2	0.979 ± 0.018	0.967
	Submucosa	4,030	3	0.960 ± 0.024	0.966
	Mucus	1,215	3	0.987 ± 0.012	0.965
	Muscularis	2,752	2	0.941 ± 0.019	0.795
	Adipocytes	953	3	0.914 ± 0.021	0.582
Colon (Sigmoid)	Mucosa	2,692	3	0.945 ± 0.021	0.927
	Submucosa	2,628	3	0.702 ± 0.036	0.329
	Chronic colitis	541	1	0.919 ± 0.029	-
	Muscularis	5,545	5	0.972 ± 0.009	0.984
	Adipocytes	823	2	0.855 ± 0.046	0.642
	Adventitia	1,030	1	0.834 ± 0.031	-
	Autolysed mucosa	196	1	0.816 ± 0.092	-
Esophagus Mucosa	Epithelium	1,530	5	0.919 ± 0.038	0.917
	Smooth muscle	2,963	4	0.873 ± 0.045	0.846
	Stroma	4,328	5	0.851 ± 0.038	0.856
	Inflammation	884	5	0.937 ± 0.027	0.933
	Erosive esophagitis	747	1	0.973 ± 0.026	-
	Submucosal gland	1,822	3	0.923 ± 0.027	0.940
	Congestion	630	1	0.886 ± 0.027	-
Mammary Tissue Breast	Duct	471	3	0.495 ± 0.078	0.316
	Stroma	11,399	8	0.963 ± 0.021	0.969
	Adipocytes	12,820	7	0.976 ± 0.011	0.966
	Lobule	803	3	0.940 ± 0.019	0.965
	Nerve	328	3	0.829 ± 0.039	0.547
	Gynecomastoid hyperplasia	262	3	0.771 ± 0.097	0.554
Skin	Dermis	1,464	2	0.973 ± 0.018	0.874
	Eccrine gland	763	2	0.885 ± 0.038	0.956
	Hair follicle	960	2	0.850 ± 0.034	0.612
	Papillary dermis	218	2	0.288 ± 0.066	0.047
	Adipocytes	2,727	2	0.903 ± 0.028	0.957
	Sebaceous gland	653	2	0.847 ± 0.044	0.898
	Squamous epithelium	470	2	0.819 ± 0.067	0.423
	Solar elastosis	178	2	0.820 ± 0.084	0.576
	Vessel	630	1	0.835 ± 0.049	-

Supplementary Table 1: Accuracy of all the annotated image derived phenotypes across 6 tissues and their cross validation variability.

3. Regarding Supp. Fig. 19, I think the analysis should go beyond the statement “we see that tile representations of breast-cancer relevant annotations cluster into distinct groups”. For instance, silhouette scores should be reported as on GTEx. Also, while the results seem to be rather satisfactory from the figure, the clustering is (unsurprisingly) not as “clear” as in GTEx. In particular, some structures are divided into several sub-clusters, while nerves appear to be intertwined with stroma, vessels, and sometimes adipocytes. The authors should elaborate on that.

We thank the reviewer for their suggestion. We agree that similar clustering quantification as performed on GTEx should also be performed on TCGA. We have now included a silhouette score (0.159) for TCGA-BRCA and updated Lines L576-L580 accordingly.

With respect to the clustering of BRCA-TCGA tiles, it is not surprising that a model trained on normal histology (GTEx) performs worse when applied to a dataset of breast carcinomas. Therefore we would expect a lower silhouette score representing clusters that are less well defined. Additionally, classes like stroma can be both benign and malignant - Malignant stroma is of course never seen in normal tissue histology. Finally, nerves present in TCGA-BRCA are small and often found in tiles with stroma, blood vessels and adipocytes - Their overlapping clustering likely represents a limit in our resolution (128x128 tiles) which we have discussed possible ways of overcoming in the discussion.

We would emphasize that externally validating a normal histology model on a carcinoma dataset represents an extreme (chosen due to data availability) and therefore the fact our representations and RNAPath predictions provide sensible inferences demonstrates the lack of overfitting on GTEx and its generalisation.

4. The sentence “we see that areas of carcinoma are well segmented from benign tissue” (l. 528-529) does not refer to any quantitative metric. AUC and/or sensitivity/specificity should be reported to support this statement. Generally speaking, the validation of the feature extractor on TCGA-BRCA through weakly-supervised segmentation lacks precision.

We thank the reviewer for their comment. We agree that a quantitative metric should be reported to support the above statement about carcinoma area segmentation. Therefore, we annotated regions of carcinoma in ten WSI from the TCGA-BRCA cohort and computed the Intersection over Union (IoU) between the ground truth segmentation and our carcinoma predictions. We see robust segmentation performance, with mean IoU = 0.723 ± 0.065. We have added examples of these segmentations to our supplementary figures, for example:

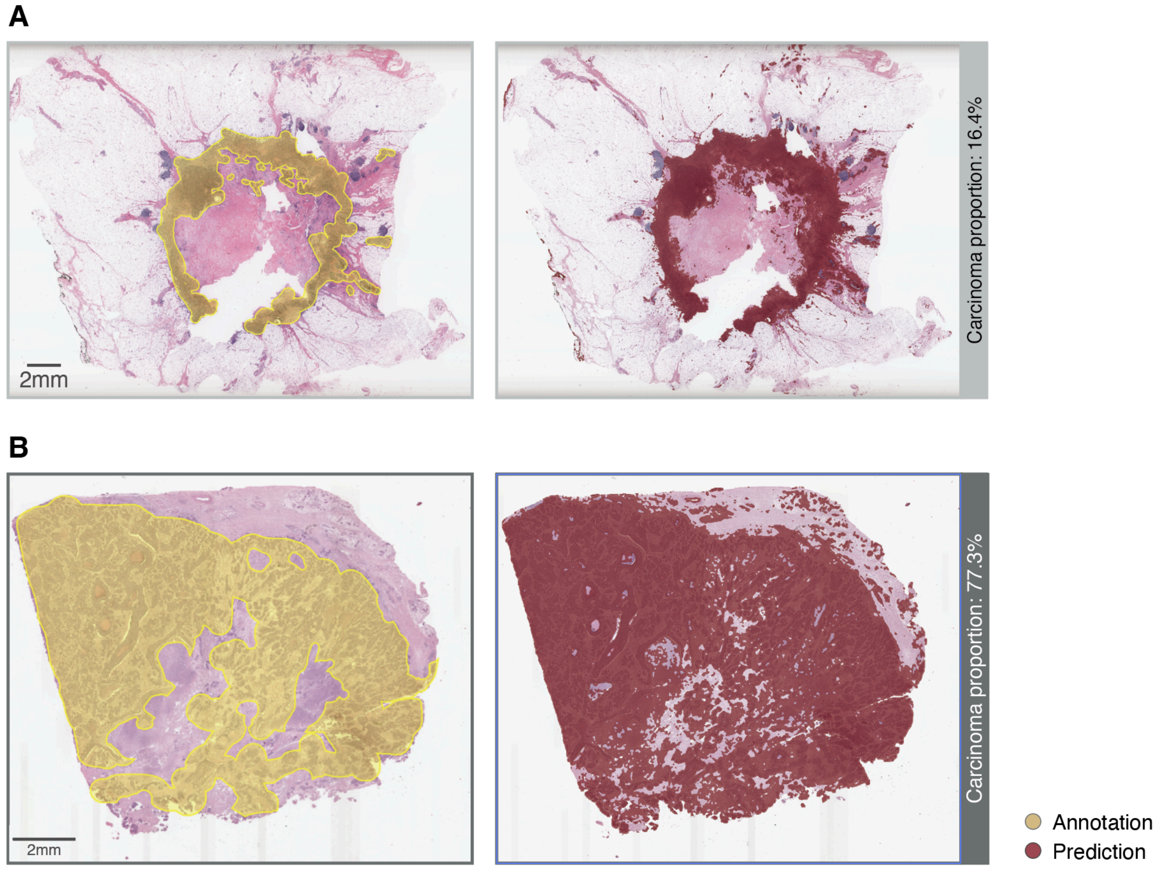

Supplementary Figure 20: Carcinoma area annotation (A, C) and prediction (B, D) for two TCGA-BRCA samples.

We believe that this provides a quantitative metric of how well our self-supervised representations perform in segmenting carcinoma, something they were not trained (or fine-tuned) to do.

5. It is difficult to conclude anything from the survival analysis carried out on TCGA-BRCA (l. 532-535). The idea that the amount of tumor on the slide is linked to prognosis seems reasonable, but, even if the p-value is significant, the separation between the two subgroups is not extremely convincing (the confidence intervals of both subgroups overlap). Also, what happens to the intermediate patients (those with a tumor percentage between the 25th and the 75th percentile)? If there is no strong message here, I suggest simply removing this part.

We thank the reviewer for their suggestion. As our study is largely focused on normal histological variation and TCGA-BRCA only represents an external validation cohort for our approach, we agree with the reviewer that the survival analysis does not add much to the manuscript. We have therefore removed the survival analysis from **Figure 7** and instead

expanded the quantitative evaluation of RNAPath predictions and our carcinoma segmentation performance.

6. L. 548, the statement "we see RNAPath is able to predict known marker genes, for example, PLIN1 in adipocytes" should be backed by a metric. Since it is the only true validation of RNAPath on external data (the model is finetuned afterward), this part should be developed more.

We thank the reviewer for their suggestion. We computed the Pearson correlation coefficient between the gene expression predictions on breast carcinoma samples (TCGA) by the RNAPath model trained on the GTEx breast cohort and the bulk RNA-seq provided by TCGA. We added a quantitative evaluation of the performance of the RNAPath model trained on the GTEx breast cohort when validated on TCGA-BRCA. We developed the sections adding some examples of both good and poor generalization which we have reproduced below:

*"With no fine-tuning of our mammary tissue RNAPath GTEx model, we see RNAPath is able to predict known marker genes, for example, PLIN1 in adipocytes (r score = 0.29, P-value = 2.27×10^{-19}) (**Supplementary Figure 19B**) or breast cancer related genes like AQP1 (r score = 0.32, P-value = 1.58×10^{-23}). However, many other genes that are correctly regressed in the GTEx dataset have a smaller correlation coefficient when evaluated on TCGA-BRCA, like MEOX2 (r score = 0.85 in GTEx, 0.21 in TCGA) or DLK2 (r score = 0.84 in GTEx, 0.16 in TCGA). This could reflect the fact that many relationships between healthy tissue morphology and gene expression learnt by the model trained in the **healthy** breast GTEx cohort are not conserved in breast cancer resection tissue and therefore fine-tuning should be employed to learn disease-specific changes."*

7. If CCNE1 and PGR expressions are predictors of the subtype, in a sense the model has access to the subtype information: if the model is able to predict the expression of CCNE1 and PGR, it is able to predict the subtype, and conversely. I do not think it is an issue as such, but I would remove "despite having access to no subtype information" (l. 558-559).

We thank the reviewer for their comment; as suggested, we removed the sentence "despite having access to no subtype information" from lines L626-L627.

8. Finally, regarding the prediction of local gene expression, the authors say they "do not make any claims that a given tile-level prediction's scale should or could match what the equivalent piece of tissue expression for a given gene would be in reality if we had a means to measure it locally". This seems contradictory with what is written l. 435-439: "As well as bulk level predictions, RNAPath provides tile level (128×128 pixel) expression predictions which can be used to create spatial expression maps of any specific across a histology sample."

Moreover, in the comparison with IHC, the authors refer to it as the “ground truth”, but I find the term misleading. From a Machine Learning perspective, I would expect the “ground truth” to refer to the spatial gene expression (which could be obtained from spatial transcriptomics or double staining or adjacent sections with H&E and IHC) on the very same sample. Here, the predicted gene expression is compared to the IHC marker on completely different samples. While it highlights the fact that the model does associate the expression of the 4 selected genes to relevant histological structures, I insist it is a qualitative validation.

Altogether, I think this part should be rewritten to clarify what exactly the experiments are probing.

We thank the reviewer for their advice on rewriting the section and we have done so to clarify specifically what the experiments are answering. Instead of ground truth, we have used the biologically correct term, positive controls. This is because these genes are known to be specifically expressed in the corresponding tissue substructures mentioned. For example, as *RNAPath* predicts *PLIN1* in adipocytes, we can use the fact that *PLIN1* is well known biologically to be specific to adipocytes. Using a matched tissue section (which we do not have access to from either GTEx or TCGA) would produce the same result, adipocytes would be selectively stained for *PLIN1*.

We have also tried to make it clear that *RNAPath* is predicting the expression of these genes solely from tissue morphology alone. We can assume that genes exhibit variance attributable to both morphology and non-morphological factors. In these cases, *RNAPath* will only explain the variance attributable to morphology, whilst spatial transcriptomics would explain all variance measurable (e.g. intracellular, environment, genetic, morphological etc). Therefore, whilst predicted tile expression correlates to ground truth expression, we would not expect a perfect match between predicted expression and ground truth expression. This is both to be expected, and fine for our use case of highlighting gene-morphology relationships and how we can already use this information for many useful downstream inferences. We have added these important points and caveats to our discussion.

Reviewer #3 (Remarks to the Author):

Thanks for addressing my concerns, no further comments

We are pleased that we were able to address all of the reviewers' concerns and thank them for their suggestions and improvements to our work.

REVIEWER COMMENTS

Reviewer #1 (Remarks to the Author):

The authours have addressed all my comments apart from Q2.

RE Q2: I appreciate the concern over computational cost. I'm afraid I disagree that it is not justified. The issue I see is that the ViT was trained on the entire dataset. The kNN was later evaluated on the same (subset of) data the encoder was trained on. Even if the training was done with the self-supervised method, the encoder could still have picked up associations with labels. The author's cross-validation therefore has a real risk of training and test spillover. I also appreciate the results on TCGA-BRCA. However, it is more of an out-of-distribution task. I believe it doesn't replace the importance of proper cross-validation in the discovery set.

I would accept a small-scale cross-validation on partial data. The variability of tissue architectures captured in encoder representations played a key role in eQTL and expression prediction tasks. Having a measure of the impact of potential technical variability due to training setting would be important evidence.

Reviewer #2 (Remarks to the Author):

I welcome the efforts made by the authors to address all of my concerns. I do not have any further comment.

Reviewer #1 (Remarks to the Author):

The authors have addressed all my comments apart from Q2.

RE Q2: I appreciate the concern over computational cost. I'm afraid I disagree that it is not justified. The issue I see is that the ViT was trained on the entire dataset. The kNN was later evaluated on the same (subset of) data the encoder was trained on. Even if the training was done with the self-supervised method, the encoder could still have picked up associations with labels. The author's cross-validation therefore has a real risk of training and test spillover. I also appreciate the results on TCGA-BRCA. However, it is more of an out-of-distribution task. I believe it doesn't replace the importance of proper cross-validation in the discovery set.

I would accept a small-scale cross-validation on partial data. The variability of tissue architectures captured in encoder representations played a key role in eQTL and expression prediction tasks. Having a measure of the impact of potential technical variability due to training setting would be important evidence.

We understand the reviewers' concern and we thank them for considering the computational challenge their question presents. Therefore, to address their concerns and to make it tractable, we have performed a 3-fold cross validation of our DINO encoder across the major tissue types that make up all of our downstream analyses and use-cases. We have then assessed the impact of these different validation splits on our downstream analyses.

Specifically, we utilised all GTEx samples belonging to 7 well represented tissue types in GTEx (**WSI n = 6319**) (tibial artery, esophagus mucosa, transverse colon, sigmoid colon, breast, sun exposed skin and suprapubic skin). These are the primary tissues that make up the majority of all downstream analysis presented in the manuscript. **Any WSI that had annotations associated with them, were completely held out of both training and validation.** We divided the remaining slides into 3 separate groups and, for each fold, we extracted 500,000 patches. To train the DINO encoders, we utilised 12 GPUs across 3 compute notes, running in parallel for > 1 week.

This analysis enabled us to answer the following questions:

1. If we hold out different validation sets, does the encoder's ability to represent substructures vary significantly?
2. If we completely hold out samples in which ground truth annotations were labelled, do we see a drop in kNN performance?
3. Does *RNAPath* expression prediction variability change depending on the validation fold a DINO model was trained on?

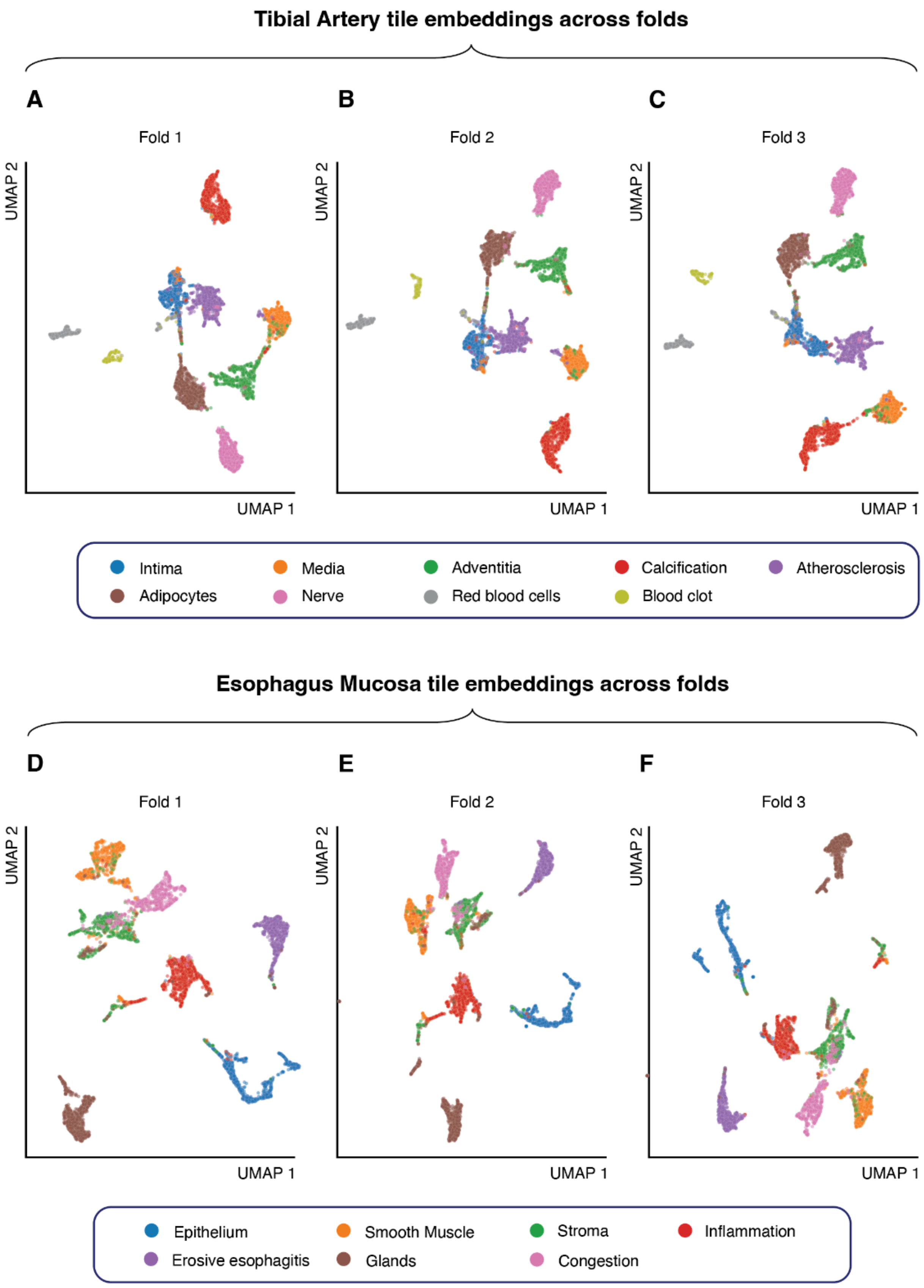
Tibial Artery tile embeddings across folds
A
Fold 1
B
Fold 2
C
Fold 3
UMAP 2
UMAP 1
Intima
Media
Adventitia
Calcification
Atherosclerosis
Adipocytes
Nerve
Red blood cells
Blood clot
Esophagus Mucosa tile embeddings across folds
D
Fold 1
E
Fold 2
F
Fold 3
UMAP 2
UMAP 1
Epithelium
Smooth Muscle
Stroma
Inflammation
Erosive esophagitis
Glands
Congestion

New Supplementary Figure 5: UMAP embeddings of tibial artery tile features from DINO model training fold 1 (A), 2 (B) and 3 (C). UMAP embeddings of esophagus mucosa tile features from DINO model training fold 1 (D), 2 (E) and 3 (F).

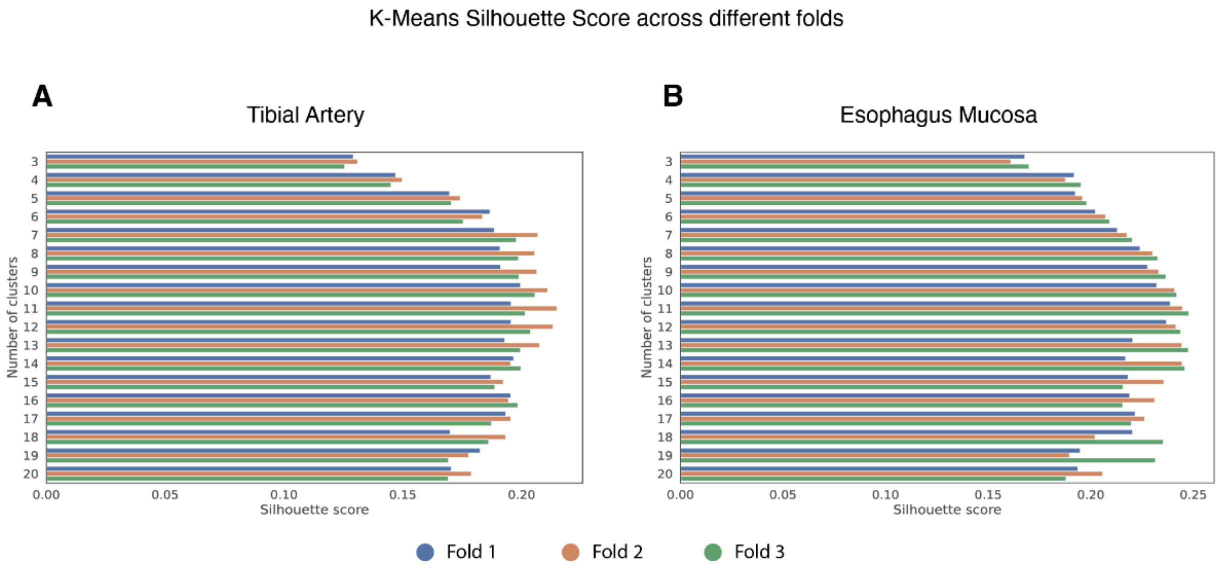

New Supplementary Figure 6: Comparison of silhouette scores across several possible cluster values k=[3,20] for tibial artery (**A**) and esophagus mucosa (**B**). Models are compared by DINO model training fold (1, 2, 3); the plots clearly show little difference in clustering across folds.

By evaluating the models relative to the annotated patches of tibial artery and esophagus mucosa, we see that the embeddings still cluster by substructure/localised pathology, as in the original model and results (**Supplementary Figure 5**). Moreover, clusters are consistent across the 3 folds. This is further confirmed quantitatively by computing silhouette scores (**Supplementary Figure 6**):

- Tibial artery: 0.190, 0.195 and 0.193 for fold 1, 2, 3 respectively (std = 0.003)
- Esophagus mucosa: 0.219, 0.228 and 0.226 for fold 1, 2, 3 respectively (std = 0.005)

Additionally, we evaluated the kNNs ability to accurately predict/classify substructure tiles relative to ground truth annotation labels across folds.

**Tissue**	**Image Derived Phenotype**	**Fold 1**	**Fold 2**	**Fold 3**
	Tunica intima	0.928	0.923	0.920
	Tunica media	0.914	0.917	0.915
	Tunica adventitia	0.890	0.889	0.893

**Tibial Artery**	Calcification	0.939	0.938	0.935
	Atherosclerosis	0.937	0.926	0.927
	Adipocytes	0.972	0.973	0.976
	Nerve	0.949	0.943	0.940
	Red blood cells	0.959	0.963	0.964
	Blood clot	0.785	0.833	0.855
**Esophagus mucosa**	Epithelium	0.920	0.911	0.914
	Smooth muscle	0.867	0.872	0.869
	Stroma	0.874	0.879	0.880
	Inflammation	0.943	0.933	0.942
	Erosive esophagitis	0.973	0.977	0.971
	Submucosal gland	0.906	0.911	0.895
	Congestion	0.875	0.878	0.870

Supplementary Table for reviewer: Accuracy of the annotated image derived phenotypes from tibial artery and esophagus mucosa across DINO training folds.

We performed a 10-fold cross validation of the annotated patches (as in **Supplementary Table 1**). Overall the median accuracy per tissue across folds is consistent, 0.930 ± 0.005 for tibial artery and 0.904 ± 0.007 for esophagus mucosa. There is one exception (blood clot) out of the 16 image derived phenotypes tested, which varies by 6.5% between folds. This is explained by the fact it is the IDP with the fewest ground truth annotations. It is also worth noting this class was not used in any downstream analysis. These results highlight that the change in validation fold has **little to no impact** on the training of our DINO encoder and downstream inferences.

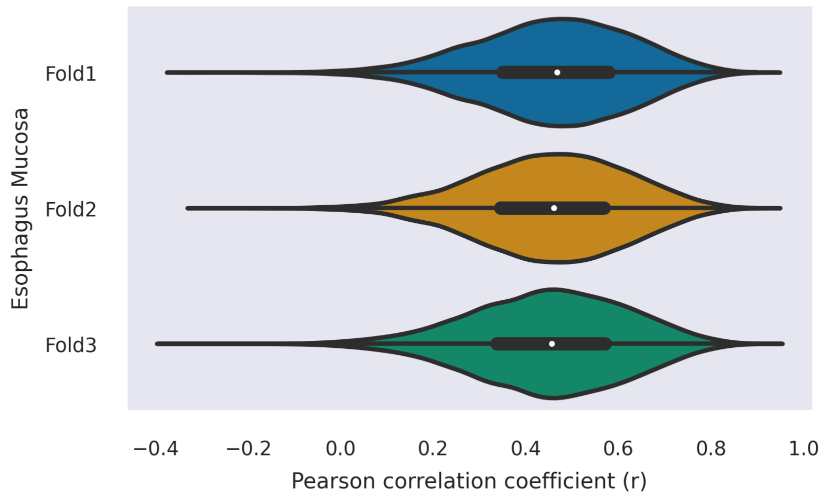

Supplementary Figure for reviewer: *RNAPath* accuracy, measured by computing the Pearson correlation coefficient (r) between expression prediction and bulk RNA-seq, for Esophagus Mucosa across the three self-supervised training folds.

Finally, to see whether there is any difference in DINO encoder representations obtained across folds for downstream gene expression inference, we trained three *RNAPath* models, one per DINO encoder trained using each of the three folds. Mean accuracy of predicted gene expression in esophagus mucosa was 0.461 ± 0.006, demonstrating little to no impact from DINO encoder variability. Furthermore, gene correlation coefficients show little variation across folds (std = 0.027).

Overall, we can see that there was no training/test set spillover due to training DINO on all GTEx samples, nor did we see any impact of validation fold / cross validation training of DINO on our downstream analysis. We hope these experiments satisfy the reviewers' concerns, as we believe these analyses are convincing in demonstrating our model is well trained and our downstream conclusions and analyses are sound.

Reviewer #2 (Remarks to the Author):

I welcome the efforts made by the authors to address all of my concerns. I do not have any further comment.

We thank the reviewer for improving our manuscript and we are pleased to have addressed all of their concerns.

REVIEWERS' COMMENTS

Reviewer #1 (Remarks to the Author):

I thank the authors for the additional cross-validation results. I agree with the authors that they have proved that their approach is robust to splits.

The authors have addressed all of my comments.

Reviewer #1 (Remarks on code availability):

Comprehensive instructions in the GitHub repo. Trained model weights are shared, I don't expect issues with reproducibility here.
